# Defining an evolutionarily conserved role of GW182 in circular RNA degradation

**DOI:** 10.1038/s41421-019-0113-y

**Published:** 2019-09-17

**Authors:** Ruirui Jia, Mei-Sheng Xiao, Zhengguo Li, Ge Shan, Chuan Huang

**Affiliations:** 1 School of Life Sciences, Chongqing University, Chongqing, 401331 China; 2 Center of Plant Functional Genomics, Institute of Advanced Interdisciplinary Studies, Chongqing University, Chongqing, 401331 China; 3 Department of Biochemistry and Biophysics, University of Pennsylvania Perelman School of Medicine, Philadelphia, 19104 PA USA; 4 School of Life Sciences, University of Science and Technology of China, Hefei, 230027 Anhui China

**Keywords:** RNA decay, Long non-coding RNAs, RNA decay, Long non-coding RNAs

Dear Editor,

Circular RNAs (circRNAs) are covalently closed RNA molecules derived from thousands of protein-coding genes via “backsplicing”. In many cases, the “backsplicing” step can be trigged by the flanking complementary intronic repeat elements that efficiently bring the intervening splicing sites into close proximity^1^. Most circRNAs are cytoplasmic, and nuclear export of mature circRNAs is regulated in a length-dependent manner^2,3^. While the functions of circRNAs remain largely unknown, recent reports have revealed that some circRNAs can control gene expression by affecting transcription, acting as splicing regulators and mircoRNA sponges^1^. It is also becoming evident that circRNAs are associated with several diseases such as cancer and brain disorders^1^. Due to the lack of a defined 5′ or 3′ end, circRNAs are naturally more stable than their parental linear RNAs as they are not targets for the exosome or exonuclease. This was exemplified by the circRNAs derived from *Drosophila* dati and laccase2 gene or our previously described expression plasmids (Fig. 1a; Supplementary Fig. S1). Nevertheless, how circRNAs are degraded or what factors contribute to a surveillance pathway is largely unclear.

**Fig. 1:**
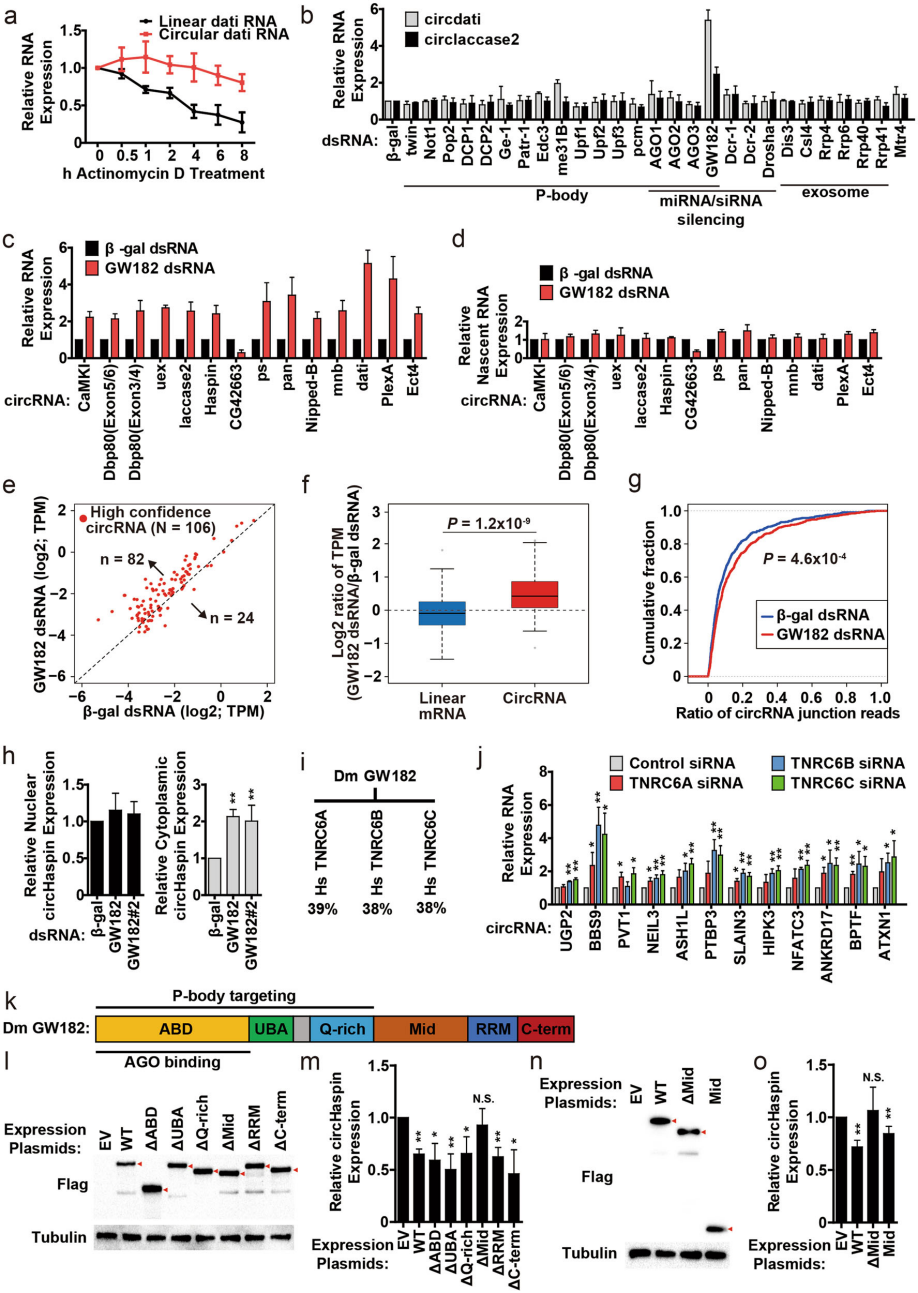
An evolutionarily conserved role of GW182 in circular RNA degradation. **a** To measure RNA half-lives, *Drosophila* DL1 cells were treated with actinomycin D for the indicated amounts of time. qRT-PCR was then used to quantify expression of endogenous circRNA and its parental linear RNA. Data were normalized to the RNA levels observed at 0 h actinomycin D treatment sample. *n* = 3. **b** qRT-PCR quantification of endogenous circdati and circlaccase2 in RNA purified from DL1 cells that were treated with the indicated dsRNAs for 3 days. **c** DL1 cells were treated with β-gal dsRNA (as a control) or GW182 dsRNA for 3 days. qRT-PCR was then performed to measure expression of the indicated steady-state circRNAs. **d** After dsRNA treatment, 250 µM 4sU was added to the cells to label nascent transcripts for 5 min prior to RNA isolation. qRT-PCR was then employed to measure levels of nascent circRNAs. Data throughout (**b**–**d**) were normalized to the β-gal dsRNA sample. *n* = 3. **e** The abundance of high confidence circRNAs (TPM ≥ 0.1) in *Drosophila* S2 cells was measured by RNA-seq upon GW182 depletion. Each dot represents one circRNA. **f** Boxplot shows that levels of the high confidence circRNAs significantly increased in GW182-depleted cells while their parental mRNAs abundance decreased slightly. **g** Cumulative distribution functions of circRNA junction read ratio for each backsplicing site in β-gal dsRNA or GW182 dsRNA sample. Statistical significances of data throughout (**f**, **g**) were computed using the Mann–Whitney *U* test. *n* = 3. **h** *Drosophila* S2 cells were treated with β-gal dsRNA or two independent GW182 dsRNAs for 3 days. qRT-PCR quantification was then used to measure levels of endogenous circRNAs in RNA purified from nuclear (Nuc) or cytoplasmic (Cyto) fractions. *n* = 4. **i** The similarity (similar amino acid properties) between *Drosophila* GW182 and its human homologs. **j** Human HeLa cells were treated for 48 h with a control siRNA or specific siRNAs to knockdown TNRC6A, TNRC6B or TNRC6C. qRT-PCR was then performed to measure levels of the indicated human circRNAs. Data were normalized to the control siRNA sample. *n* = 3. **k** Schematic representation of *Drosophila* GW182. Expression plasmids of GW182 mutants were generated. **l**–**o** Overexpression of GW182 mutants to identify the key domain of GW182 in circRNA degradation. **l** and **n** western blotting was used to examine expression of the Flag-tagged GW182 proteins. α-Tubulin was used as a loading control. Representative blots are shown. *n* = 3. **m** and **o** qRT-PCR quantification of *Drosophila* endogenous circRNAs in RNA purified from S2 cells that were transfected with wild type (WT) or the indicated mutants of GW182 plasmids for 48 h. **m** *n* = 3. **o** *n* = 4. Data were normalized to the empty vector (EV) sample. All data are shown as mean ± SD. ^∗∗^*P* < 0.01; ^∗^*P* < 0.05

To reveal the factors which are required for degradation of circRNAs, we employed a focused RNAi screening in *Drosophila* DL1 or S2 cells targeting 31 genes with known roles in RNA metabolism, and then assessed the levels of steady-state circRNAs by qRT-PCR (Fig. 1b; Supplementary Figs. 2–3. In line with previous study, knockdown of RNA decay factors Pop2 (also known as CAF1), Not1, and DCP2 led to significant accumulation of Vha68-1 mRNA^4^ (Supplementary Fig. S2b), but had no effect on the levels of steady-state circRNAs (Fig. 1b). We instead found that depletion of GW182 resulted in accumulation of both steady-state circdati and circlaccase2 transcripts (Fig. 1b). GW182 is a key component of P-body and RNAi machine as it facilitates the assembly of P-body and acts as a molecular scaffold bringing together RNA-induced silencing complexes and various mRNA decay enzymes^5^. However, depletion of other P-body components or RNAi machine factors did not have effect on steady-state circRNA levels, indicating that P-body or RNAi machine does not affect circdati or circlaccase2 degradation (Fig. 1b).

To examine whether GW182 exerts a general or limited role in controlling circRNA levels, we tested the levels of 12 additional circRNAs that were of varying length and exon counts in GW182-depleted DL1 cells (Fig. 1c; Supplementary Table S1). The levels of most steady-state circRNAs were significantly increased upon GW182 depletion. Importantly, the role of GW182 appears to be robust in affecting circRNA stability because (i) the levels of most nascent circRNAs were not affected upon GW182 depletion, suggesting that circRNA biogenesis is largely unaffected by GW182 (Fig. 1d; Supplementary Fig. S4a, b), (ii) circRNA accumulation was also observed in GW182-depleted *Drosophila* S2 cells genome widely (Fig. 1e–g; Supplementary Figs. S4–S6), (iii) the enriched circRNAs were also verified using a secondary GW182 dsRNA (Supplementary Fig. S4c, d), (iv) overexpression of GW182 decreased the levels of steady-state circRNAs (Supplementary Fig. S4e), (v) depletion of GW182 had no effect on nuclear circRNA levels, while cytoplasmic circRNAs accumulated (Fig. 1h; Supplementary Fig. S7), and (vi) GW182 depletion had little effect on degradation of circRNAs’ parental mRNAs (Fig. 1f, g; Supplementary Figs. S4d, S6c–e). Taken together, these data suggested that GW182 is involved in degradation of many circRNAs (Fig. 1b–h).

Considering that GW182 is evolutionarily conserved from *Drosophila* to human, we next tested whether human homologs of *Drosophila* GW182 similarly control degradation of human circRNAs. Humans encode three homologs of GW182—TNRC6A, TNRC6B, and TNRC6C, which are 38–39% similar to *Drosophila* GW182 (Fig. 1i; Supplementary Fig. S8a; Supplementary Protein Information). To examine the role of human homologs of GW182 in circRNA degradation, TNRC6A, TNRC6B, or TNRC6C was depleted in HEK293 or HeLa cells by siRNAs. The levels of 12 steady-state human circRNAs of varying length and exon counts and plasmid-derived circRNAs were then tested by qRT-PCR or Northern blots (Fig. 1j; Supplementary Table S2, Figs. 8–9. Likewise, depletion of the three human homologs resulted in significant accumulation of human circRNAs, indicating a conserved role of GW182 in circRNA degradation.

GW182 proteins consist of an Ago-binding domain (ABD), a ubiquitin-associated domain (UBA), a glutamine-rich domain (Q-rich), a middle region (Mid), an RNA-recognition motif (RRM), and a C- terminal region (C-term) (Fig. 1k; Supplementary Figs. S8a, S10a). To identify which domain of GW182 may contribute to circRNA degradation, we transfected *Drosophila* S2 cells with a series of GW182-overexpression plasmids that harbored various domain deletions (Fig. 1l, m; Supplementary Fig. S10). GW182 mutants without ABD, UBA, Q-rich, RRM, or C-term domain were still sufficient to accelerate circRNA degradation, but mutant without Mid had no effect on circRNA levels. As expected, overexpression of Mid domain alone was also able to decrease circRNA levels, indicating that Mid domain of GW182 might be important to circRNA degradation (Fig. 1n, o; Supplementary Fig. S11). Since ABD domain of GW182 mediates the interaction with Ago proteins in RNAi pathway, and ABD, UBA, and Q-rich domains play an important role in localization of GW182 to P-body^6^ (Supplementary Fig. S12), it indicated that GW182 might regulate degradation of at least a subset of circRNAs in Ago-slicer or P-body independent manner, a result that is consistent with our RNAi screening data (Fig. 1b).

Taken together, our study demonstrates a novel regulatory role of *Drosophila* GW182 and its human homologs (TNRC6A, TNRC6B, and TNRC6C) in degradation of circRNAs. A previous study reported that a near perfect miR-671 target site of CDR1as/ciRS-7 can trigger cleavage of this circRNA in an Ago2-slicer-dependent manner^7^, but it is important to note that many circRNAs do not contain potential microRNA target sites that induce Ago-2 cleaving^8^, and few circRNAs exhibit properties expected of miRNA sponges^9^. GW182 is usually thought to act as a key factor in P-body or RNAi silencing pathway. However, depletion of other core components of P-body or RNAi machine did not change the levels of mature circRNAs (Fig. 1b), and GW182 mutants without Ago-binding ability or P-body-localization signals could still accelerate circRNA degradation (Fig. 1k–m), suggesting that P-body and RNAi machine might be dispensable in circRNA degradation pathway. On the other hand, Mid domain seems to be involved in circRNA degradation (Fig. 1k–o). Previous studies demonstrated that Mid domain could serve as a molecular scaffold for the recruitment of various RNA decay factors^5^, suggesting that GW182 might mediate the interactions between circRNAs and circRNA decay factors through its Mid domain. Although GW182 and its mutants have little effect on nuclear export of circRNAs (Fig. 1h; Supplementary Figs. S7, S11), it could also be possible that GW182 might function in subcellular localization (e.g., certain specific decay granules) of circRNAs in the cytoplasm.

Although circRNAs have variable lengths, sequences, and structures, we first provided evidence that these transcripts are fed into specific degradation pathway by evolutionarily conserved factor-GW182. We believe that our work is a significant step forward in understanding of circRNA degradation and GW182, and represent an advance to lead further studies.

## Supplementary information

Supplementary RNA-seq data.

Supplementary Information.

## Supplementary information

**Supplementary Information** accompanies the paper at (10.1038/s41421-019-0113-y).

## References

[1] Li X, Yang L, Chen LL (2018). The biogenesis, functions, and challenges of circular RNAs. Mol. Cell.

[2] Huang C, Liang D, Tatomer DC, Wilusz JE (2018). A length-dependent evolutionarily conserved pathway controls nuclear export of circular RNAs. Genes Dev..

[3] Li Z, Kearse MG, Huang C (2019). The nuclear export of circular RNAs is primarily defined by their length. RNA Biol..

[4] Behm-Ansmant I (2006). mRNA degradation by miRNAs and GW182 requires both CCR4:NOT deadenylase and DCP1:DCP2 decapping complexes. Genes Dev..

[5] Niaz S, Hussain MU (2018). Role of GW182 protein in the cell. Int. J. Biochem. Cell Biol..

[6] Eulalio A, Helms S, Fritzsch C, Fauser M, Izaurralde E (2009). A C-terminal silencing domain in GW182 is essential for miRNA function. RNA.

[7] Hansen TB (2011). miRNA-dependent gene silencing involving Ago2-mediated cleavage of a circular antisense RNA. EMBO J..

[8] Jeck WR, Sharpless NE (2014). Detecting and characterizing circular RNAs. Nat. Biotechnol..

[9] Guo JU, Agarwal V, Guo H, Bartel DP (2014). Expanded identification and characterization of mammalian circular RNAs. Genome Biol..

