# Supplementary material for: Defining an evolutionarily conserved role of GW182 in circular RNA degradation
Source: Cell Discov. 2019 Sep 17;5:45. doi: 10.1038/s41421-019-0113-y (PMC6796862; doi:10.1038/s41421-019-0113-y)
Supplement: Supplementary file 2 — Supplementary Information. [file 41421_2019_113_MOESM2_ESM.pdf]

# Supplementary Information

## Defining an evolutionarily conserved role of GW182 in circular RNA degradation

Ruirui Jia, Mei-Sheng Xiao, Zhengguo Li, Ge Shan, Chuan Huang

**Supplementary Methods**

**Supplementary Figure S1-S12.**

**Supplementary Figure Legends**

**Supplementary Table S1-S5.**

**Supplementary Protein Information**

**Supplementary Plasmid Information**

**Supplementary RNA sequencing Data**

# Supplementary Methods

## *Drosophila* cell culture, RNAi, and transfections

*Drosophila* DL1 and S2 cells were cultured at 25°C with Schneider's *Drosophila* medium (Sigma, S9895) plus 10% fetal bovine serum (HyClone, SH30910.03) and 1% (v/v) penicillin-streptomycin (Thermo Fisher Scientific, 15140122). dsRNAs from the DRSC (*Drosophila* RNAi Screening Center) were generated by in vitro transcription (MEGAscript kit, Thermo Fisher Scientific, AM1334) of PCR templates containing the T7 promoter sequence on both ends (Supplementary information, Table S3). To measure RNA half-lives, DL1 cells were treated with actinomycin D (1 μg/mL; Sigma, A4262) for the indicated amounts of time. For RNAi experiments in DL1 cells, 0.5 million of cells in 12-well dishes were bathed with 2 μg of dsRNA for 3 days. For RNAi experiments in S2 cells, 1.5 million of cells in 12-well dishes were bathed with 8 μg of dsRNA for 3 days. RNA was isolated using Trizol (Thermo Fisher Scientific, 15596018) according to the manufacturer's instructions.

To generate plasmids for transfection into *Drosophila* DL1 and S2 cells, the indicated sequences were inserted into a pMK33/pMtHy-based vector between the metallothionein promoter (pMT) and the SV40 polyadenylation signal as described in Supplementary Plasmid Information. For circRNA expression plasmids transfection, 2 million of *Drosophila* DL1 cells in 6-well plate were transfected with 2 μg plasmid using Effectene transfection reagent (QIAGEN, 301425). A final concentration of 500 μM copper sulfate (MACKLIN, C805782) was added for 3 h to induce expression followed by washing the cells twice with media containing 500 mM bathocuproine disulphonate (BCS; Sigma, B1125-500MG). For GW182 expression plasmids transfection, 0.5 million of *Drosophila* S2 cells in 6-well plate were transfected with 1 μg plasmid using Effectene transfection reagent (QIAGEN, 301425). A final concentration of 500 μM copper sulfate was

added for 48 h to induce expression.

## Human cell culture and RNAi

Human HeLa and HEK293 cells were cultured at 37 ℃ and 5% $CO_2$ with MEM (GIBCO, C11095500BT) plus 10% fetal bovine serum and 1% (v/v) penicillin-streptomycin. RNAi was performed with Lipofectamine RNAiMAX reagent (Thermo Fisher Scientific, 13778-030) according to the manufacturer's instructions as described previously [2, 10]. For plasmid and siRNA co-transfection, HeLa cells in 6-well plate were transfected with 2 μg plasmid (pcDNA3.1(+) HIPK3 sense, Addgene #60634) and 50 pmol siRNA using Lipofectamine 2000 transfection reagent (Thermo Fisher Scientific, 11668019). Detailed information for siRNAs used is provided in Supplementary Table S3.

## Nuclear and cytoplasmic fractionation

Cellular fractionation was performed as previously described [2]. In brief, *Drosophila* S2 cells were washed twice with PBS and resuspended with slow pipetting in 1 mL lysis buffer B (10 mM Tris-HCl pH 8, 140 mM NaCl, 1.5 mM MgCl2, 0.5% IGEPAL CA-630, 1 mM dithiothreitol, and 80 U/mL RNase inhibitor (Thermo Fisher Scientific, 10777019)). Nuclei were collected by centrifugation at 1,000g for 3 min at 4 ℃ and the supernatant was saved as the cytoplasmic fraction. Nuclei were resuspended in 1 mL lysis buffer B and 100 μL of detergent (3.3% (w/v) sodium deoxycholate, 6.6% (v/v) Tween 40) was added. Samples were slowly vortexed for 10 sec and incubated on ice for 5 min. Nuclei were then collected by centrifugation at 1,000g for 3 min and washed with 1 mL of lysis Buffer B. The final pellet (nuclear fraction) was resuspended in 1 mL Trizol. Efficient fractionation of nuclear and cytoplasmic RNAs was verified by testing levels of

U6 snRNA and 18S rRNA.

**RNA-seq library preparation and data analysis**

*Drosophila* S2 cells were treated with either β-gal dsRNA or GW182 dsRNA for 3 days and then total RNA was extracted by using Trizol (Thermo Fisher Scientific, 15596018) according to the manufacturer's instructions. RNA-seq libraries were prepared by using TruSeq Stranded Total RNA Library Prep Gold kit (Illumina 20020598) following the manufacturer's instructions and were subsequently sequenced for 150 nt from both ends on Illumina HiSeq 2500 platform.

Sequencing reads were filtered by using fastp [11] to remove low quality bases and adaptor sequences from both ends of reads. The remaining reads were mapped to the *Drosophila* genome (BDGP6.22, downloaded from Ensembl) using TopHat2 (version 2.0.14) with default parameters [12]. For each library, gene expression levels were estimated by using Cufflinks version 2.2.1 [13] based on Ensembl annotation (BDGP6.22, version 96). In order to compare the gene expression levels between β-gal dsRNA and GW182 dsRNA samples, the FPKM (Fragments Per Kilobase per Million mapped reads) of each gene was converted to TPM (Transcripts Per Million mapped reads) [14]. As most circRNAs were derived from protein coding genes, only protein coding genes were retained for further analysis.

To identify circRNAs with CIRI2 (version 2.0.6) [15], RNA-seq reads were first mapped to *Drosophila* genome (BDGP6.22, downloaded from Ensembl) with bwa [16] and then the alignments were inputted to CIRI2 with default parameters. At least two junction reads in one of the libraries was required for a circRNA to be retained for further analysis. Expression levels of circRNAs were estimated by TPM (Transcripts mapped to back-splicing junctions Per Million uniquely mapped fragments). High confidence circRNAs were determined as those with TPM $\geq 0.1$ in at least two

samples. Only circRNAs derived from exon regions of individual protein coding gene according to Ensemble annotation (BDGP6.22, version 96) were retained for further analysis.

**qRT-PCR**

Complementary DNA for qRT-PCR was synthesized using PrimeScript RT Master Mix (Takara, RR036A) according to the manufacturer's instructions. qPCR was then performed in triplicate using FastSYBR Mixture (CWBIO, CW0955M). All qPCR primer sequences are provided in Supplementary Table S4 and S5.

**Metabolic labeling of nascent RNA with 4sU and nascent RNA purification**

Metabolic labeling of nascent RNA with 4sU and nascent RNA purification was performed as described previously with modifications [2, 17, 18]. *Drosophila* DL1 cells, treated with the indicated dsRNA for 3 days, were incubated with 250 μM 4sU (Sigma, T4509) for 5 min to label newly transcribed RNA. 20 μg 4sU labeled RNA was incubated in 500 μL biotinylation buffer (10 mM Tris pH 7.4, 1 mM EDTA) with 10 μg/mL MTSEA biotin-XX (Biotium, 90066; dissolved in dimethylformamide) at room temperature for 1.5 h. To verify that there was not significant variation across the biotinylation reactions, 2 ng of synthetic RNA was included in each reaction (Supplementary information, Table S5). Unbound MTSEA biotin was removed by equal volume Chloroform:Isoamyl alcohol (Sigma, C0549-1PT) extraction, and RNA was precipitated at 12,000g for 30 min at 4 ℃ with 1:10 volume of 5 M NaCl and an equal volume of isopropanol. The RNA pellet was washed with 80% ethanol and resuspended in 100 μL DEPC treated water. 4sU-labeled and unlabeled RNA was separated using Dynabeads™ MyOne™ Streptavidin T1 beads (Thermo Fisher Scientific, 65602). Biotinylated RNA was incubated with 100 μL

Streptavidin beads in hybridization buffer (5 mM Tris pH 7.5, 0.5 mM EDTA, 1 M NaCl) at room temperature for 1.5 h. Beads were washed 5 times with high salt washing buffer (100 mM Tris pH 7.4, 10 mM EDTA, 1 M NaCl, 0.1% Tween 20). Nascent RNA was eluted with 100 μL 0.1 M dithiotheitol (DTT) twice, and purified using RNeasy MinElute Cleanup Kit (QIAGEN, 74204).

**Northern Blotting**

Northern Blots using DIG Northern Starter Kit (Roche, 12039672910) were performed as previously described [19, 20]. Digoxin-labeled RNA probe was prepared with the corresponding PCR products as templates for T7 transcription according to the manufacturer's instructions. Blots were viewed with a Bio-Rad ChemiDoc Imaging System.

HIPK3 RNA probe sequence:

*GGTTACGGTGCTGAAAGCATTCATAAGCTCGTACAAAGTTATATTCATCAGCATTTTCAGTAC*
*TGAGCCTTGCTAATATGCTCACTTCTATTTGACCTTGACGGGCATAAGAAGGATGATTCTTCA*
*AAATTTTGATTGCTACAATTTCATTTGTCCCTCTTTTCCAGCATTTAACTACCTGGCCAAACGT*
*GCCTCGACCAAGAAAATCAAGGACTTCGTAAGTATTTTTCATGGAGCATAAGACTTCATGCT*
*GTACTAACTGATAGTCACCTTCTCC*

**Immunofluorescence staining**

Immunofluorescence staining was performed as previously described [19, 20]. Briefly, Flag_GW182 (wild type or mutants) and V5_DCP2 were co-overexpressed in *Drosophila* S2 cells that were seeded on concanavalin A-coated coverslips. Coverslips were then incubated with antibodies against Flag (Sigma, F1804, 1:100 dilution) and V5 (Proteintech, 14440-1-AP, 1:100 dilution) for 12 h at 4 ℃ followed by incubating with fluorescence secondary antibodies (Abcam, ab150077,

1:100 dilution; ab150116, 1:100 dilution). Fluorescence signals were gathered with Leica microsystems (Leica, DMIL LED Fluo).

**Western Blotting**

Whole-cell protein extracts were prepared using RIPA buffer and separated on NuPAGE 4-12% Bis-Tris gel (Thermo Fisher Scientific, NP0329BOX). The gel was then transferred to Polyvinylidene fluoride membranes (Bio-Rad, 1620177). Membranes were processed following the ECL western blotting protocol (Thermo Fisher Scientific, EI9051), as described previously [2, 19, 20]. These antibodies were used in western blots: Monoclonal Anti-FLAG® M2 antibody (Sigma, F1804, 1:1,000 dilution) and Monoclonal Anti-α-Tubulin antibody (Sigma, T6074, 1:10,000 dilution). Blots were viewed with a Bio-Rad ChemiDoc Imaging System.

**Statistical analyses**

Statistical significance for comparisons of means was assessed by Student's *t*-test for both qRT-PCR and the percentage of circRNA junction reads in total uniquely mapped fragments. Statistical details and error bars are defined in each figure legend: $P < 0.01$ ( ** ) and $P < 0.05$ ( * ). Mann-Whitney U test was used to compare the statistical difference of gene expression fold change and the ratio of circRNA junction reads in total splicing reads.

**Data availability**

The RNA-seq data generated by this study have been deposited in the NCBI GEO database under the accession number GSE132100.

**References**


10. Chekulaeva M, Mathys H, Zipprich JT *et al. Nat Struct Mol Biol 2011*; **18**:1218-1226.

11. Chen S, Zhou Y, Chen Y, Gu J. *Bioinformatics 2018*; **34**:i884-i890.

12. Kim D, Pertea G, Trapnell C, Pimentel H, Kelley R, Salzberg SL. *Genome biology 2013*; **14**:R36.

13. Trapnell C, Roberts A, Goff L *et al. Nat Protoc* 2012; **7**:562-578.

14. Wagner GP, Kin K, Lynch VJ. *Theory Biosci 2012*; **131**:281-285.

15. Gao Y, Wang J, Zhao F. *Genome biology 2015*; **16**:4.

16. Li H, Durbin R. *Bioinformatics 2009*; **25**:1754-1760.

17. Russo J, Heck AM, Wilusz J, Wilusz CJ. *Methods 2017*; **120**:39-48.

18. Zhang Y, Xue W, Li X *et al. Cell Rep* 2016; **15**:611-624.

19. Huang C, Wang X, Liu X, Cao S, Shan G. *Cell Discov 2015*; **1**:15029.

20. Li Z, Huang C, Bao C et al. *Nat Struct Mol Biol 2015*; **22**:256-264.

# Supplementary Figure S1

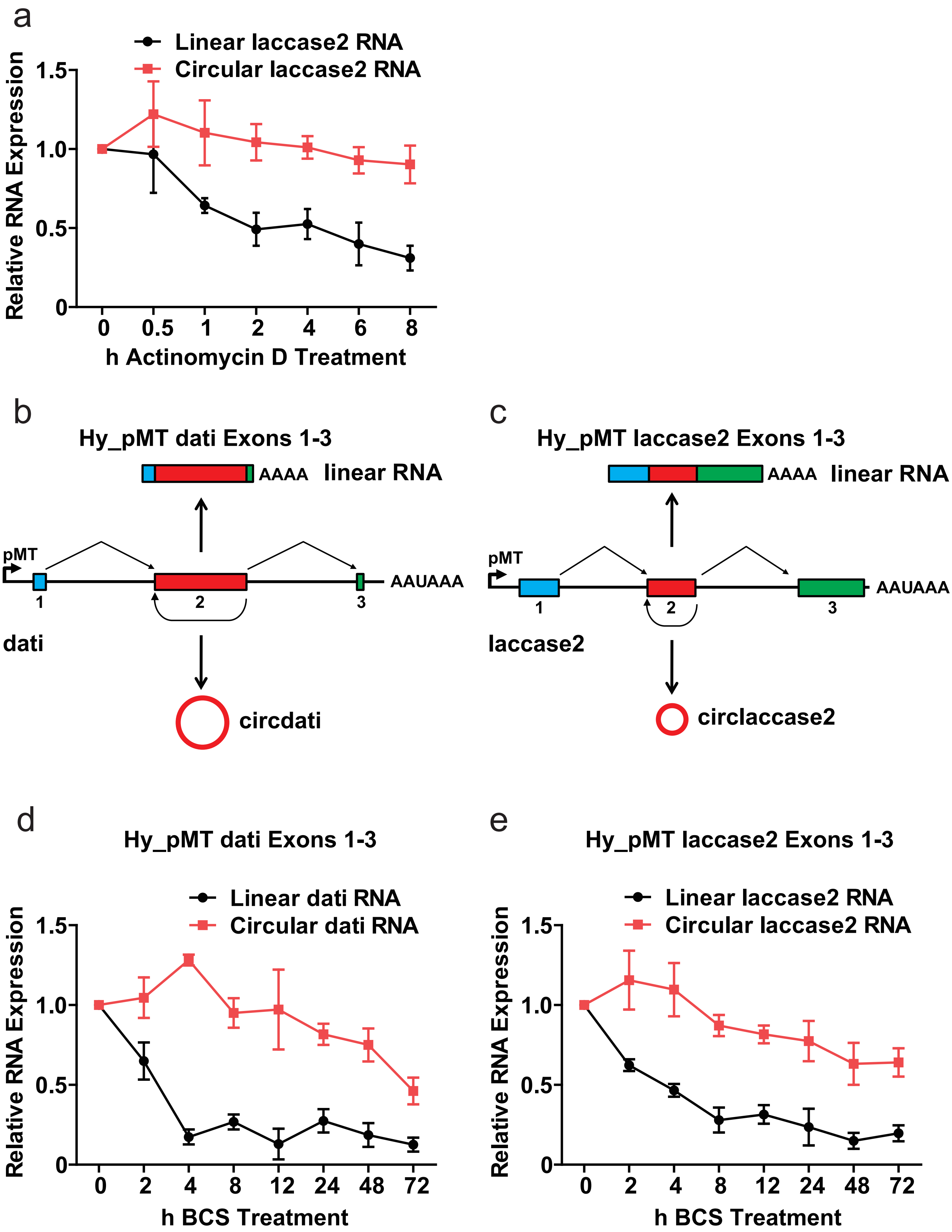

# Supplementary Figure S2

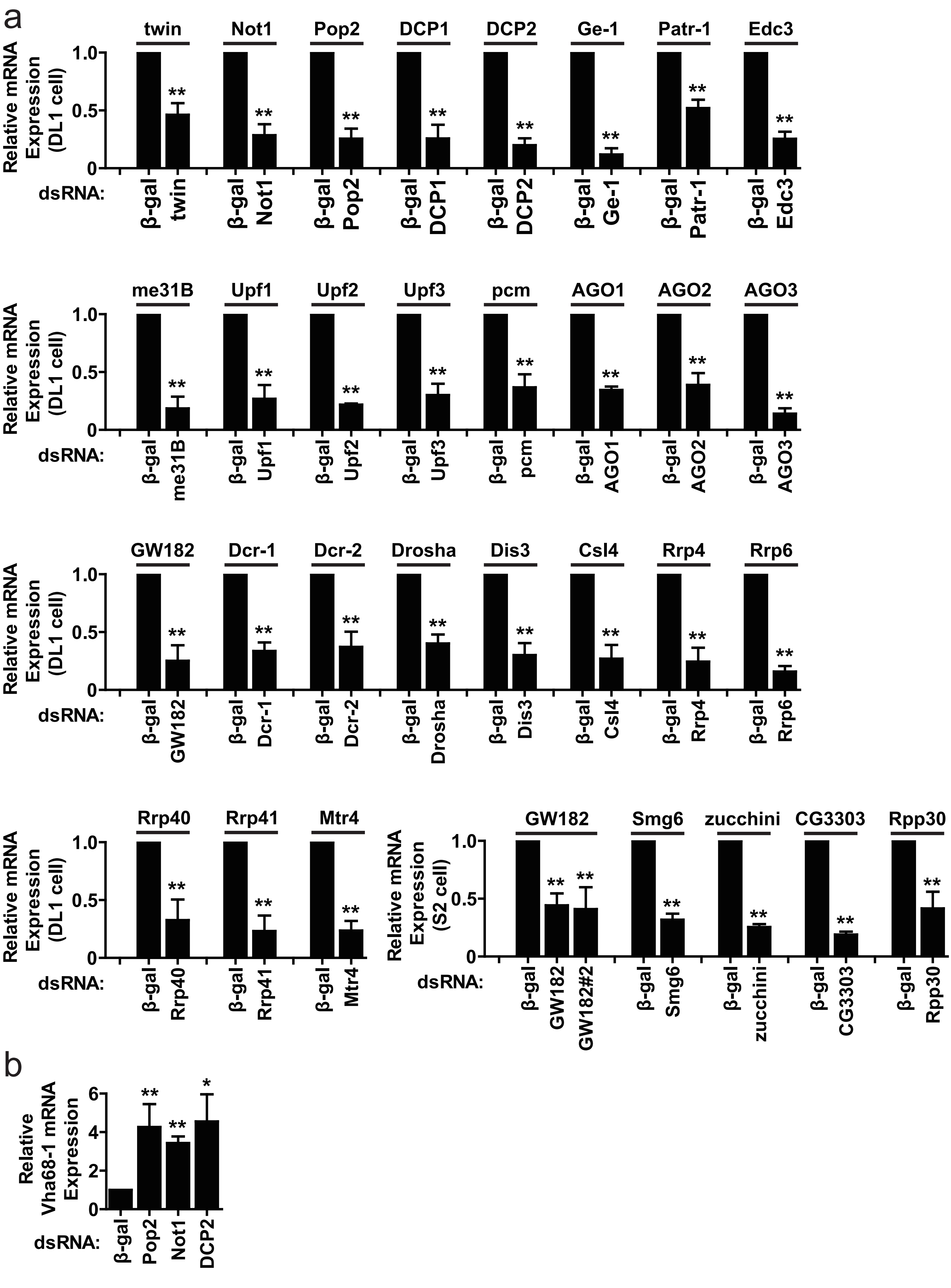

# Supplementary Figure S3

a

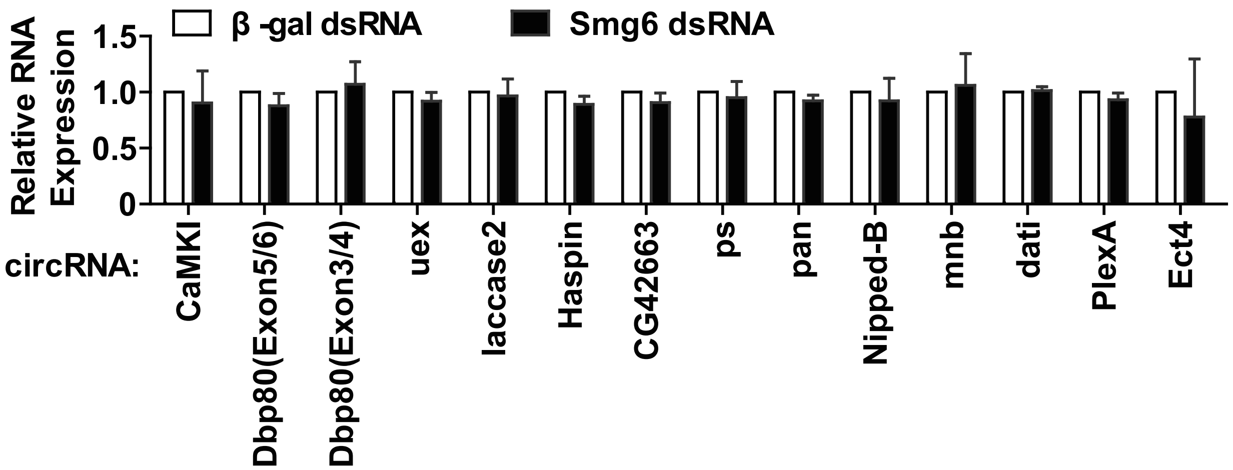


b

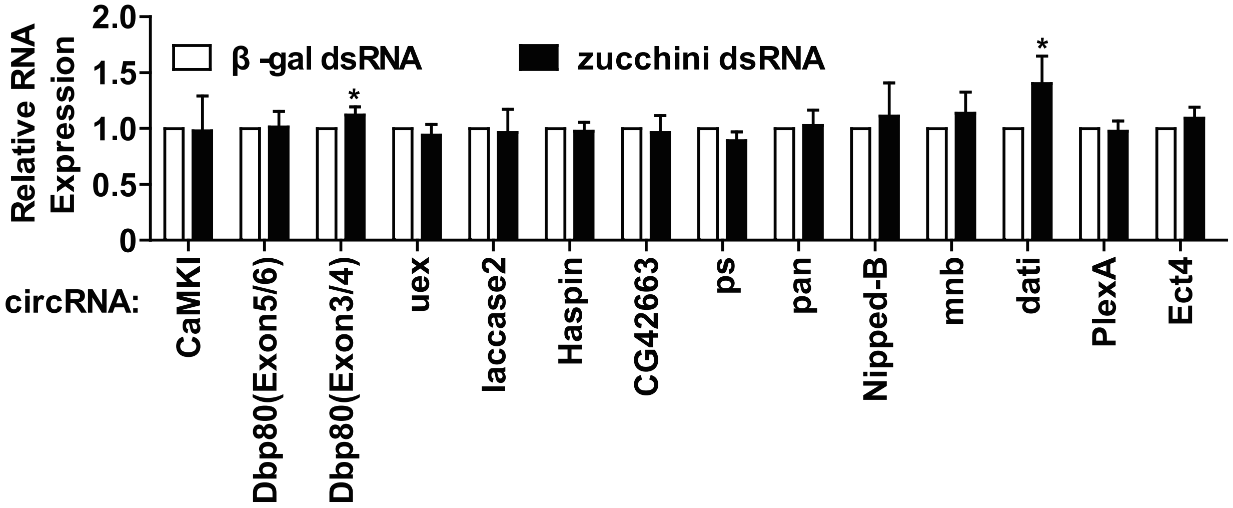


c

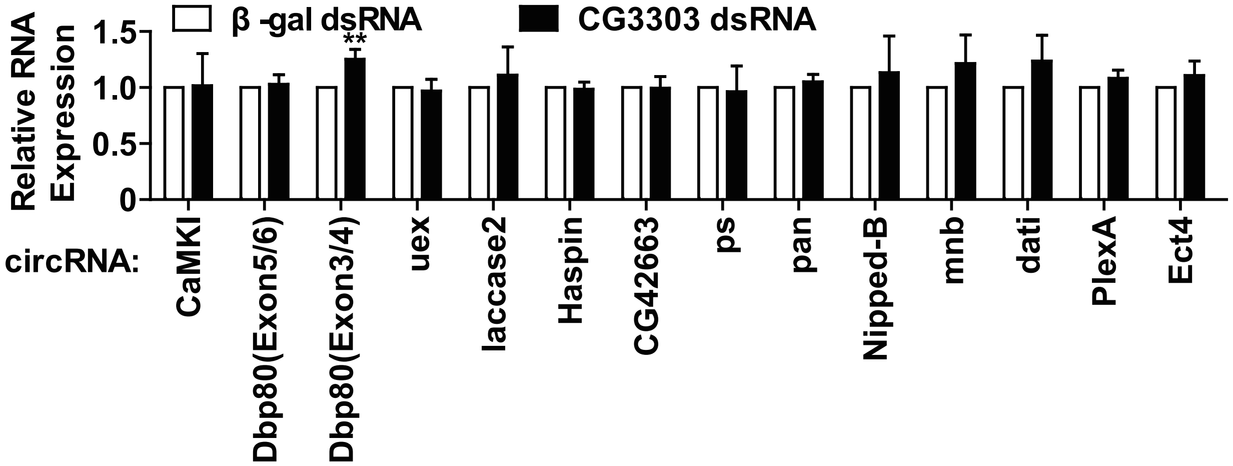


d

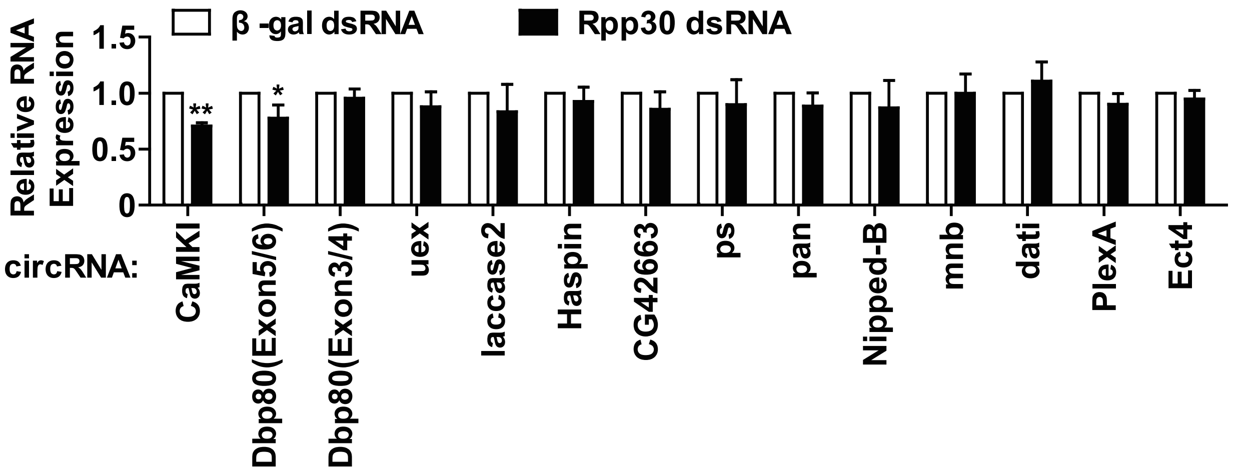

# Supplementary Figure S4

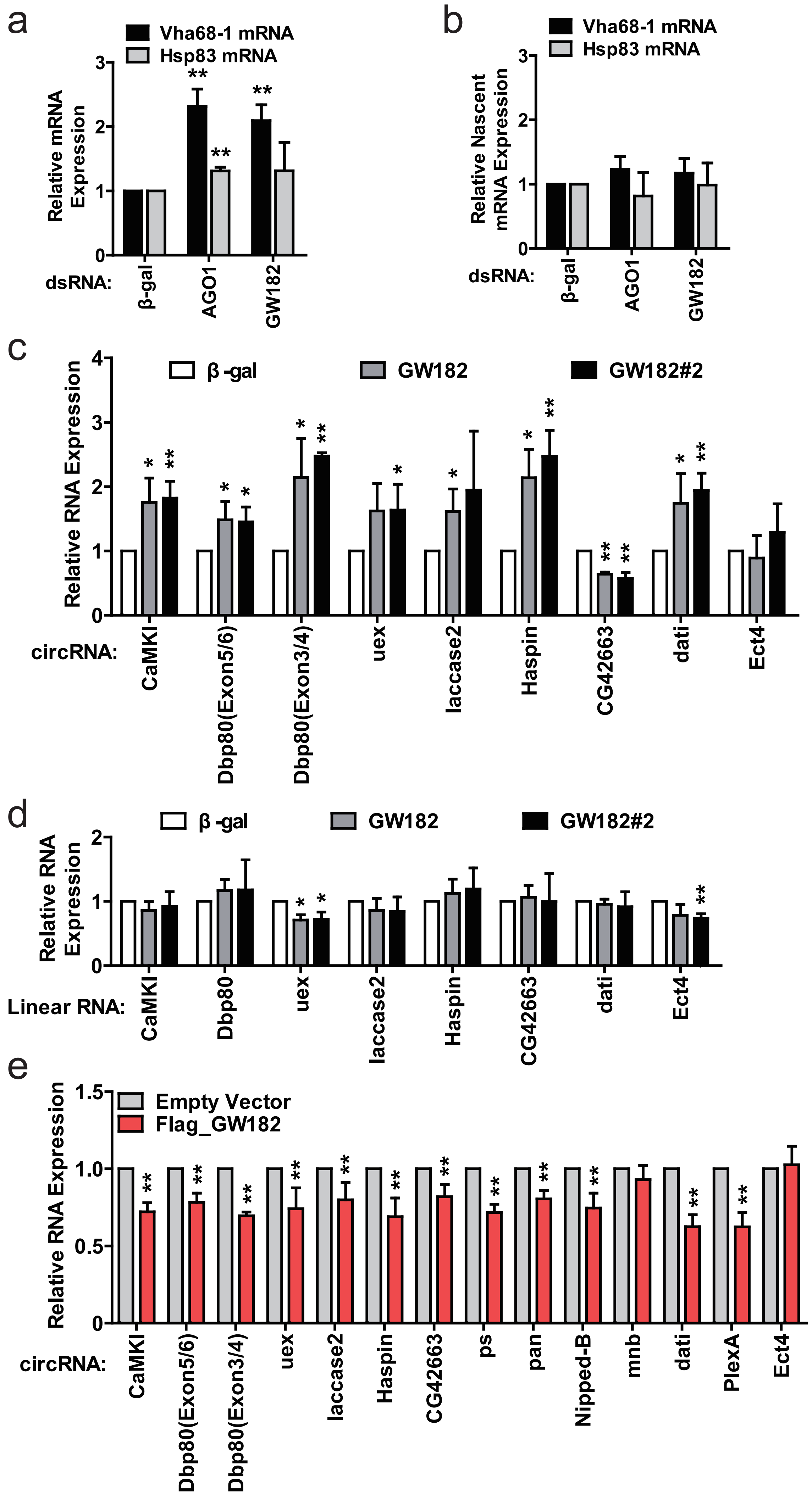

# Supplementary Figure S5

a

| | Total read pairs | Read 1 uniquely mapped | Read 2 uniquely mapped | Uniquely mapped read pairs | CircRNA junction reads |
|---|---|---|---|---|---|
| β-gal dsRNA rep 1 | 38,434,151 | 23,172,205 | 21,817,433 | 17,838,368 | 15,868 |
| β-gal dsRNA rep 1 | 49,582,654 | 30,153,563 | 27,858,670 | 22,556,390 | 18,530 |
| β-gal dsRNA rep 1 | 45,739,977 | 28,373,691 | 25,813,290 | 20,850,787 | 18,522 |
| GW182 dsRNA rep 1 | 46,738,100 | 29,535,791 | 26,883,575 | 20,890,708 | 23,331 |
| GW182 dsRNA rep 2 | 40,587,867 | 24,732,868 | 22,710,250 | 17,928,313 | 19,181 |
| GW182 dsRNA rep 3 | 41,962,039 | 24,960,191 | 23,979,344 | 18,033,569 | 18,574 |

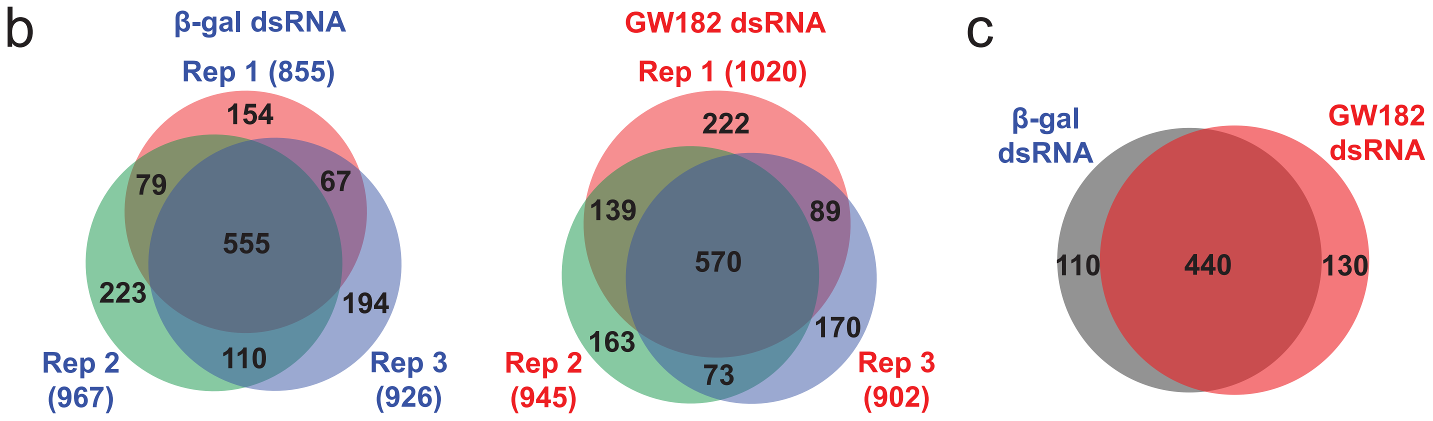

# Supplementary Figure S6

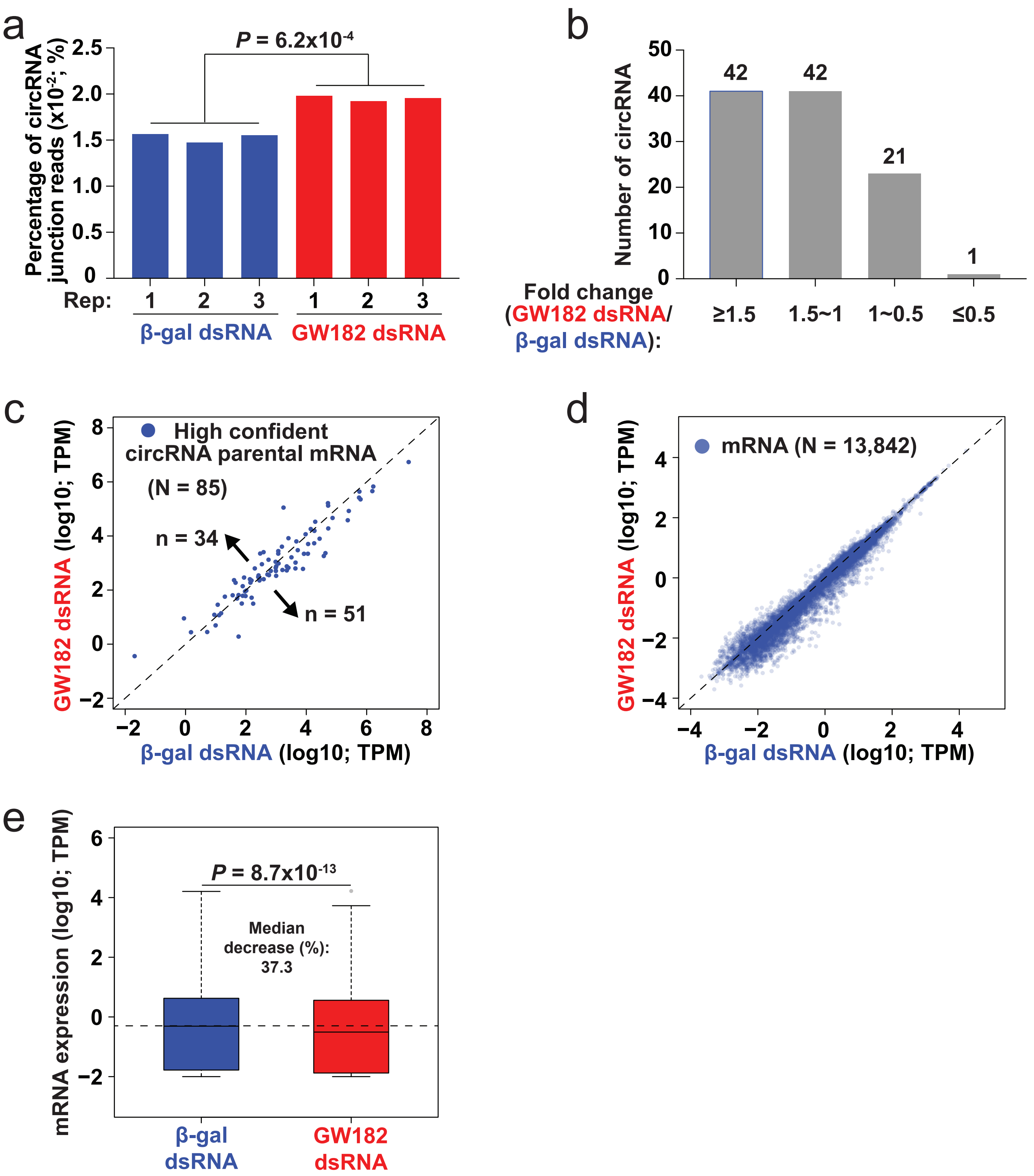

Supplementary Figure S7

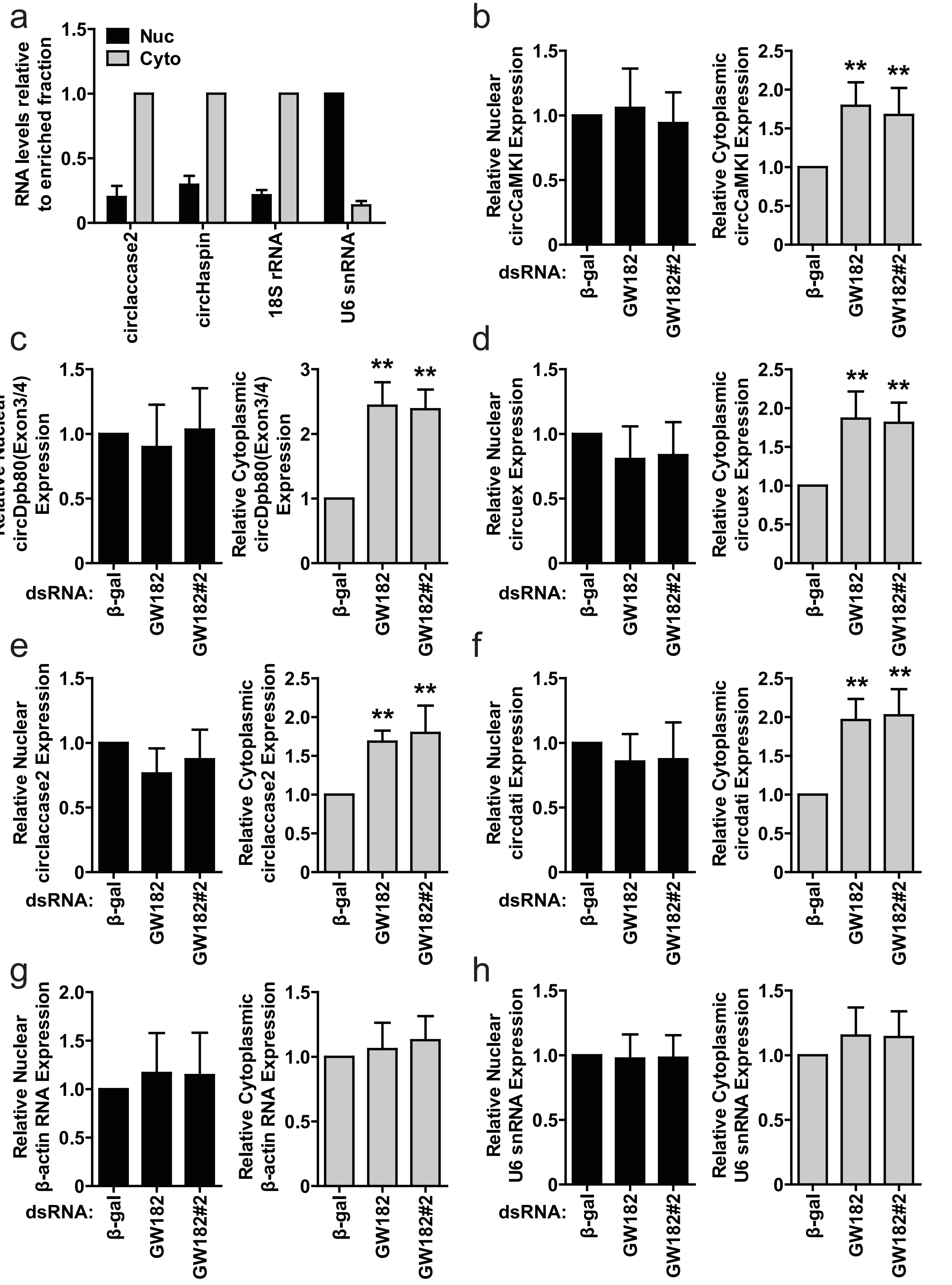

# Supplementary Figure S8

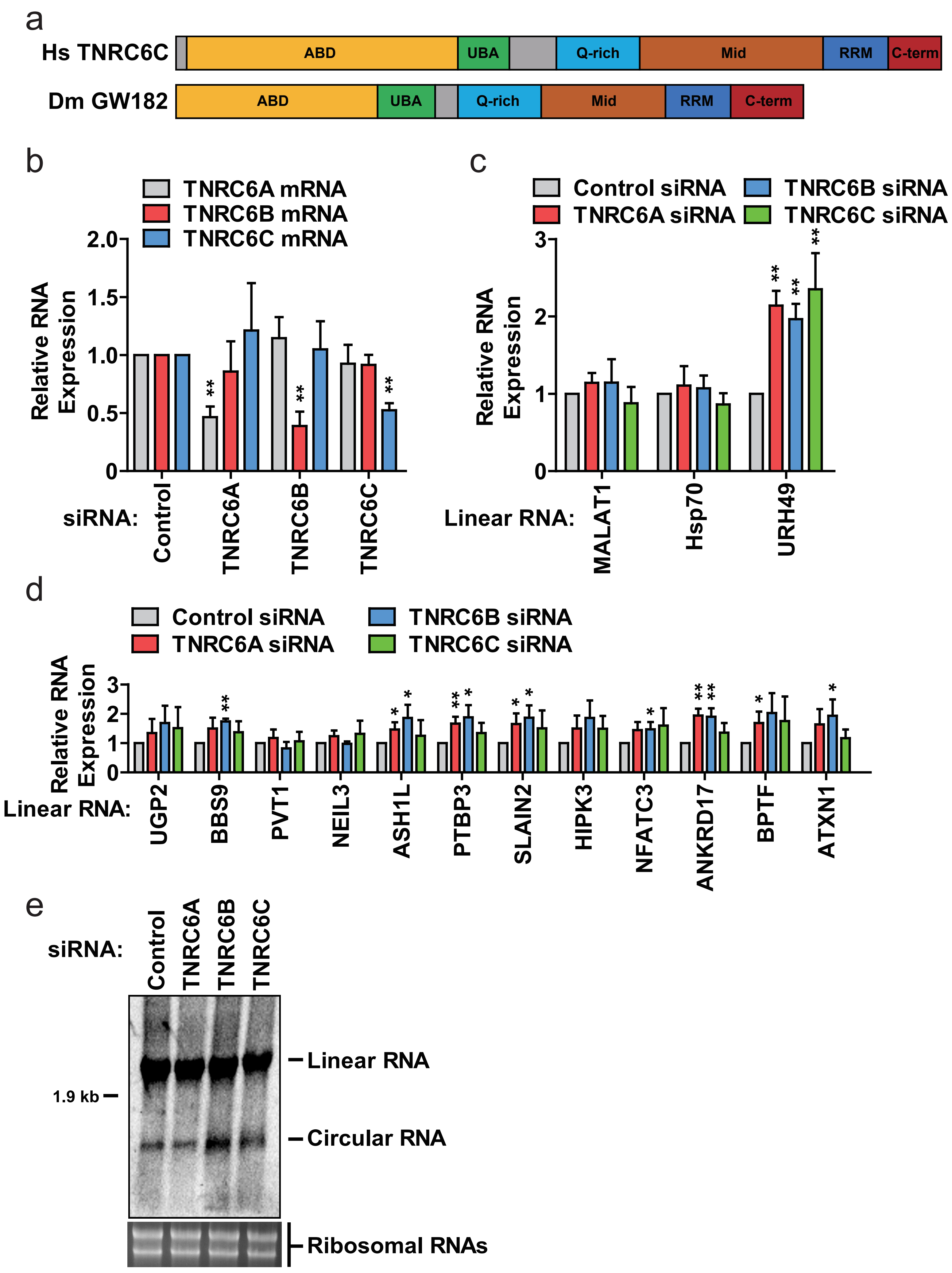

# Supplementary Figure S9

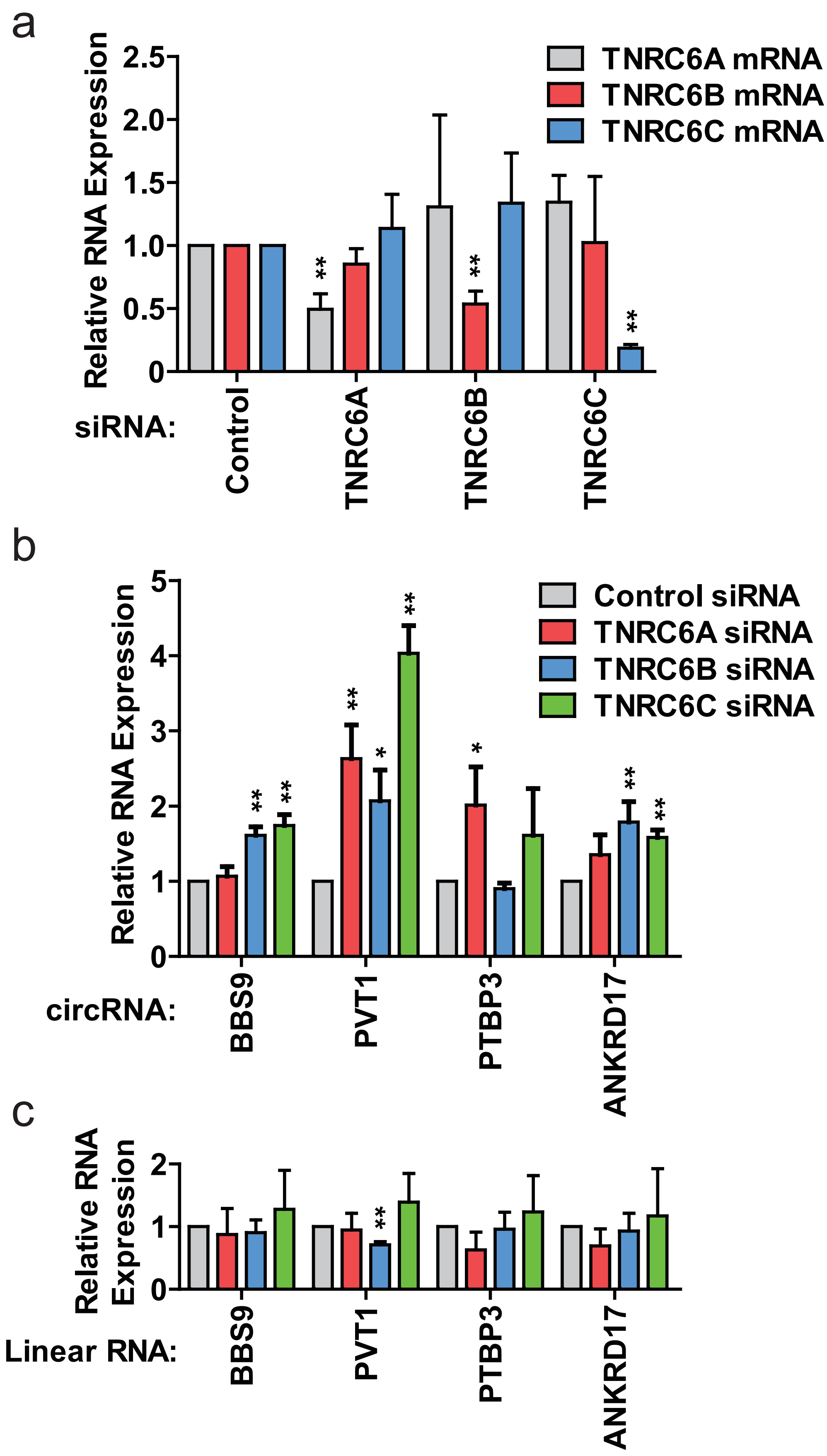

# Supplementary Figure S10

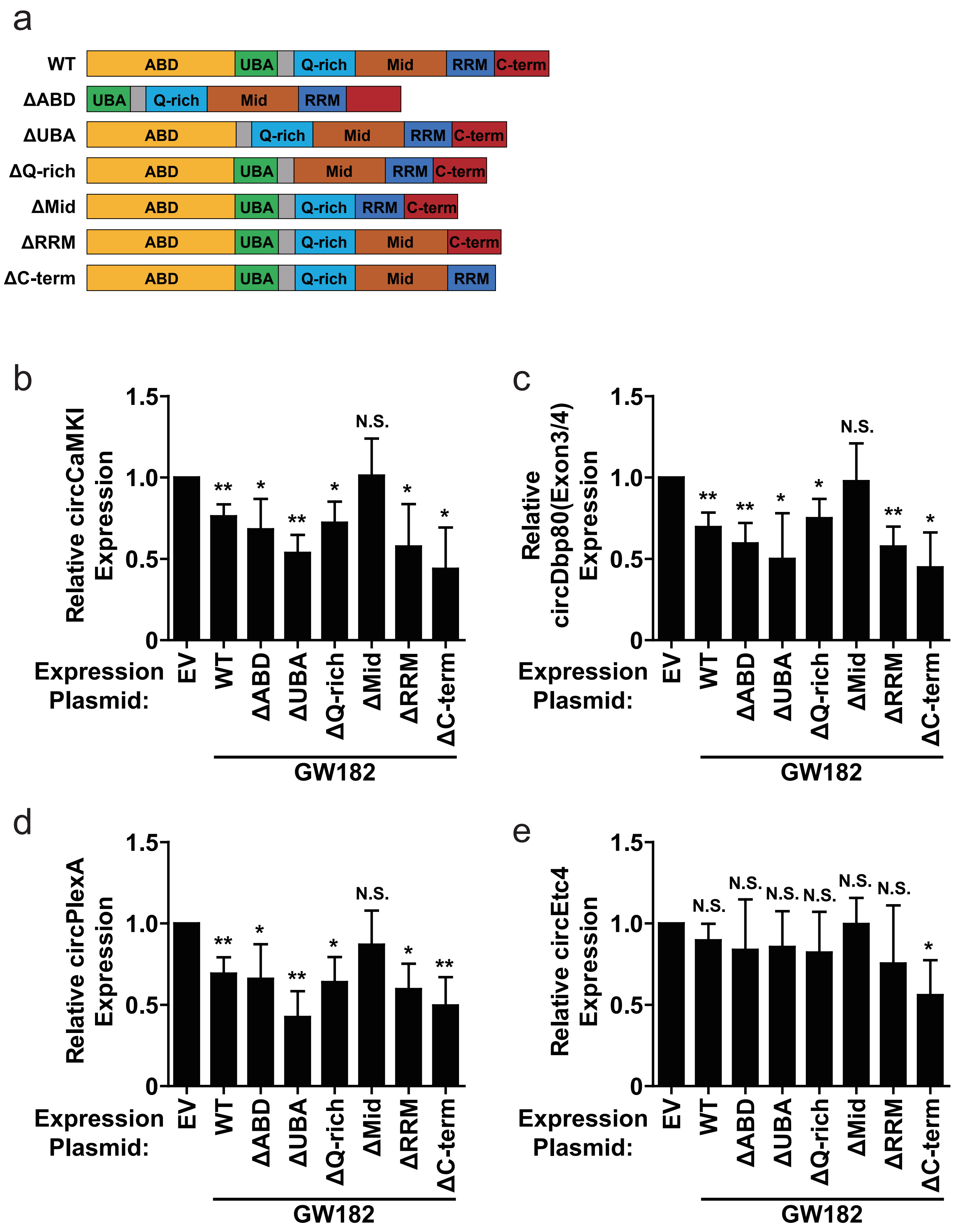

# Supplementary Figure S11

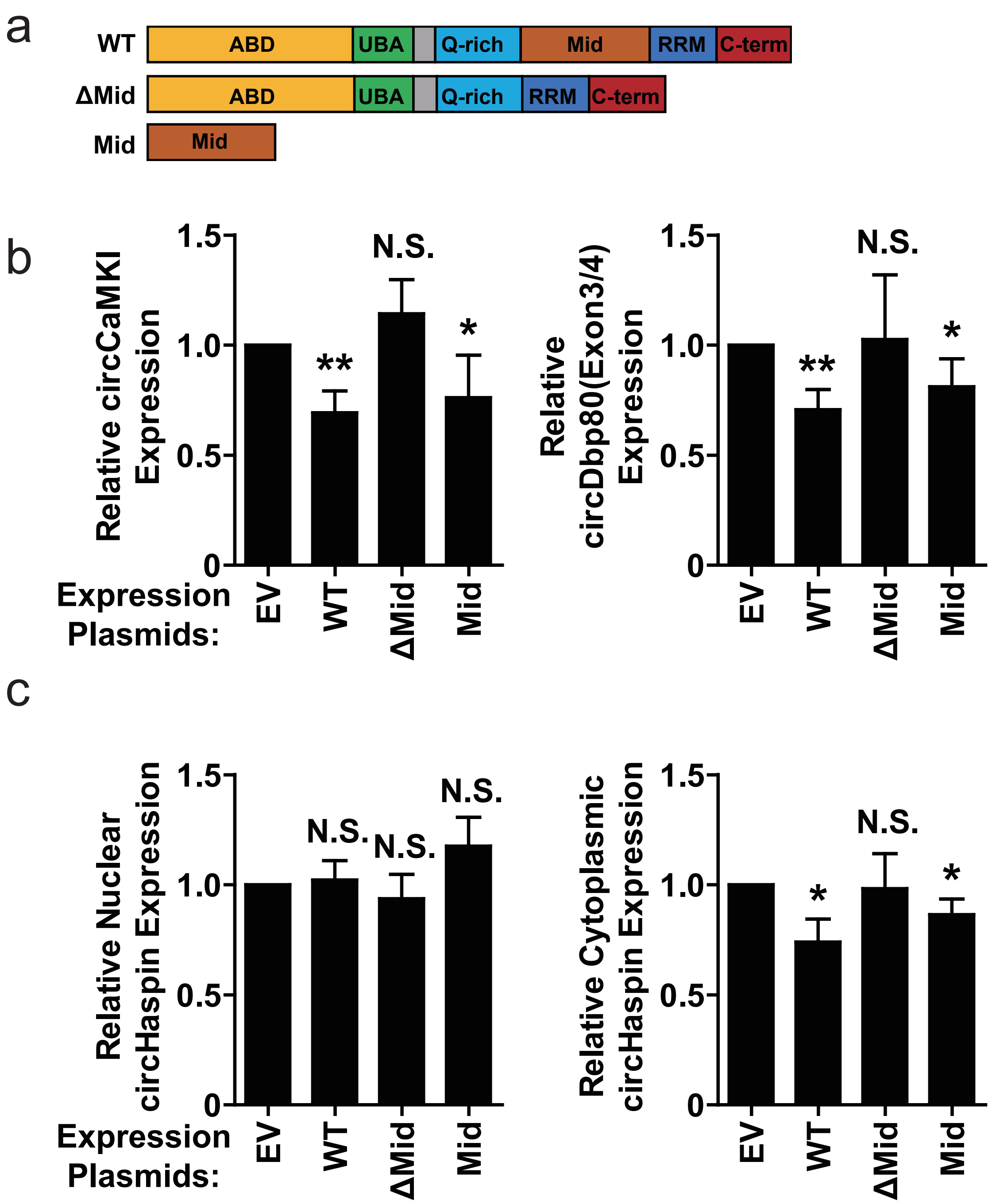

# Supplementary Figure S12

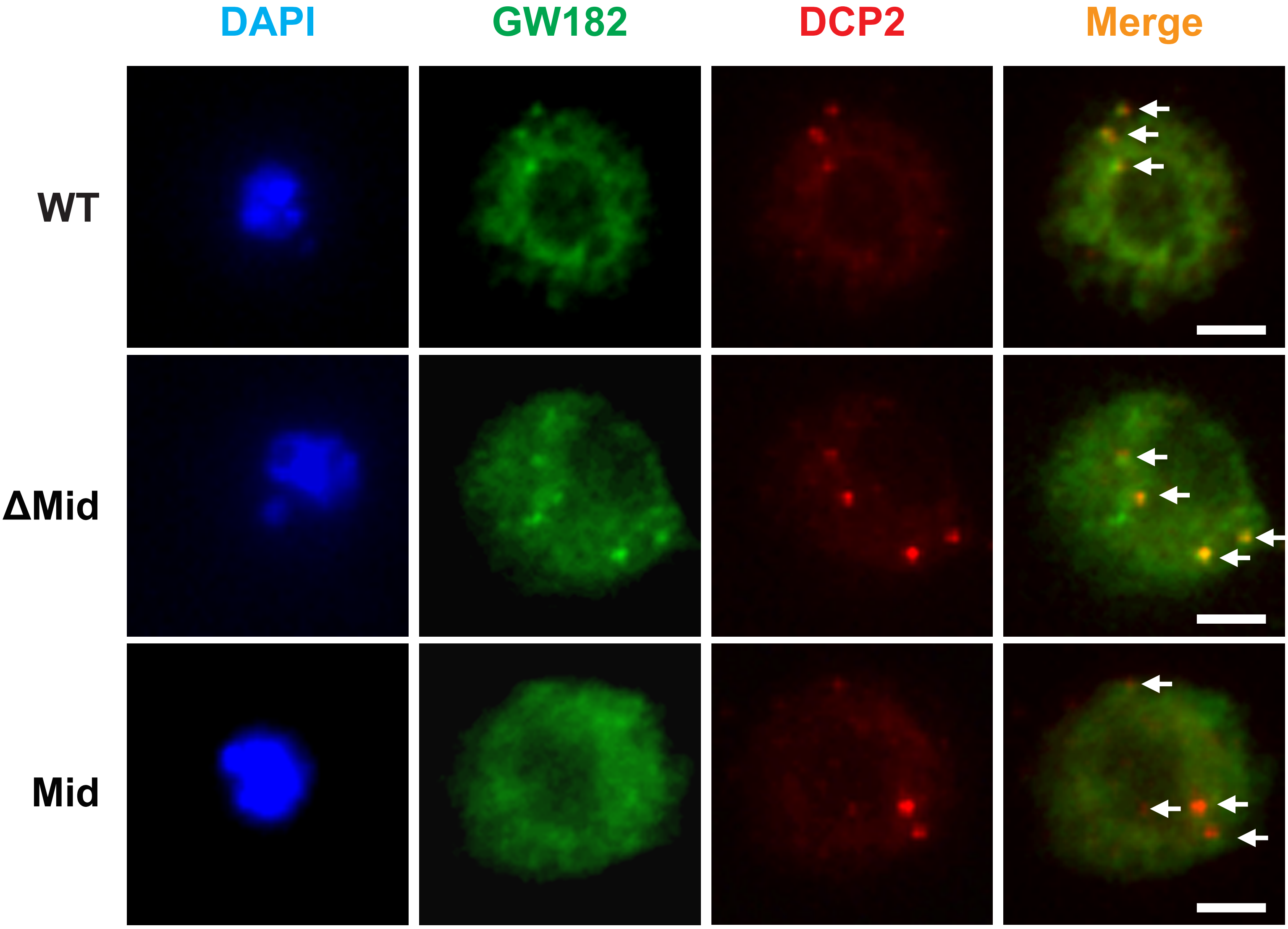

## Supplementry Figure Legends

**Figure S1. *Drosophila* circRNAs have long half-lives.**

(**a**) To measure endogenous RNA half-lives, *Drosophila* DL1 cells were treated with actinomycin D for the indicated amounts of time. qRT-PCR was then performed to quantify expression of endogenous circRNA and its parental linear RNA. Data were normalized to the RNA levels observed at 0 h actinomycin D treatment sample and are shown as mean ± SD. n = 3. (**b, c**) Schematic of "Hy_pMT dati Exons 1-3" and "Hy_pMT laccase2 Exons 1-3" reporter plasmids, which can generate linear RNAs as well as circRNAs. Exons 1, 2, and 3 with shortened intervening introns were cloned downstream from the copper-inducible metallothionein A promoter (pMT), as previously described (Liang et al. 2017, Huang et al. 2018). (**d**, **e**) To measure plasmid-derived RNA half-lives, *Drosophila* DL1 cells, transfected with "Hy_pMT dati Exons 1-3" or "Hy_pMT laccase2 Exons 1-3" reporter, were induced by $CuSO_4$ for 3 h followed by chelation of the metal with bathocuproine disulfonate (BCS) for the indicated amounts of time. qRT-PCR was then performed to quantify expression of plasmid-derived circRNAs and their parental linear RNAs. Data were normalized to the RNA levels observed at 0 h BCS treatment sample and are shown as mean ± SD. n = 3.

**Figure S2. Depletion of RNA decay factors to identify regulators of linear and circular RNA degradation.**

(**a**) *Drosophila* DL1 and S2 cells were treated with the indicated dsRNAs, and qRT-PCR was then used to verify that each dsRNA efficiently depleted its target gene. (**b**)

qRT-PCR quantification of linear Vha68-1 mRNA from DL1 cells treated with the indicated dsRNAs. Data throughout the figure were normalized to the β-gal dsRNA sample and are shown as mean ± SD. n = 3. (**) $P < 0.01$; (*) $P < 0.05$.

**Figure S3. Depletion of potential endonucleases does not affect the degradation of most tested circRNAs.**

(**a-d**) *Drosophila* S2 cells were treated with the indicated dsRNAs to knock down potential endonucleases (Smg6, zucchini, CG3303 or Rpp30). qRT-PCR quantification of endogenous circRNAs was then performed. Data throughout the figure were normalized to the β-gal dsRNA sample and are shown as mean ± SD. n = 3. (**) $P < 0.01$; (*) $P < 0.05$.

**Figure S4. GW182 selectively regulates degradation of circRNAs but with limited effect on their parental linear RNAs in *Drosophila* cells.**

(**a**, **b**) DL1 cells were treated with β-gal dsRNA (as a control), AGO1 dsRNA or GW182 dsRNA for 3 days (related to **Figure 1c, d**). (**a**) qRT-PCR was then used to measure expression of endogenous steady-state Vha68-1 and Hsp83 mRNA. (**b**) To measure the nascent transcript levels, cells were treated with 250 μM 4sU for 5 min prior to RNA isolation. Then the nascent RNAs were separated by streptavidin beads and subjected to qRT-PCR to measure the nascent Vha68-1 and Hsp83 mRNA levels. (**c**, **d**) S2 cells were treated with β-gal dsRNA (as a control) or two independent and non-overlapping GW182 dsRNAs for 3 days. qRT-PCR was then used to measure expression of the

indicated steady-state circRNAs (**c**) or their parental mRNAs (**d**). Data throughout (**a**-**d**) were normalized to the β-gal dsRNA sample. Although GW182 might act as a circCG42663 biogenesis regulator, and facilitate the production of circCG42663, the levels of most steady-state circRNAs significantly accumulated upon GW182 depletion. (**e**) qRT-PCR quantification of steady-state circRNAs in RNA purified from GW182-overexpressed S2 cells. Data were normalized to the empty vector (EV) sample. All data are shown as mean ± SD. n = 3. (∗∗) $P < 0.01$; (∗) $P < 0.05$.

**Figure S5. Statistics of RNA-seq data and circRNA identification.**

(**a**) Sequencing reads were filtered and mapped to the *Drosophila* genome (BDGP6.22, Ensembl) using TopHat2 and the mapping statistics are provided. (**b**) CIRI2 was used to predict circRNAs in each of the RNA-seq libraries. At least 2 junction reads were required for a circRNA to be identified. The Venn Diagram shows the number of circRNAs predicted in each replicate and their overlapping among three biological replicates. (**c**) Overlapping of shared circRNAs that were identified in both β-gal dsRNA and GW182 dsRNA treated samples.

**Figure S6. RNA-seq data revealed that GW182 depletion leads to accumulation of circRNAs, but not their parental linear mRNAs.**

(**a**) Comparing to β-gal dsRNA treated samples (red), the percentage of circRNA junction reads from total uniquely mapped fragments significantly increased upon GW182 depletion (blue). Unpaired Student's *t*-test was used to determine the statistical

significance. (**b**) The high confidence circRNAs were separated into four groups according to the circRNA TPM ratio (GW182 dsRNA/β-gal dsRNA). The number of circRNAs was marked on top of each bar. (**c**) The parental mRNA abundance of high confidence circRNAs (related to **Figure 1e**) was compared between β-gal controls and GW182 depleted samples. Each blue dot represents one mRNA. (**d**) Expression levels of all protein coding genes of β-gal controls and GW182 depleted samples were compared. Each blue dot represents one mRNA. (**e**) The boxplot shows that expression of all protein coding gene in GW182-depleted cells was significantly lower than that in control cells. Mann-Whitney U test was used to calculate the statistical significance.

**Figure S7. GW182 depletion has no effect on nuclear export of circular and linear RNAs, and GW182 accelerates degradation of circRNAs in the cytoplasm.**

(**a**) qRT-PCR quantification of endogenous *Drosophila* circRNAs in RNA purified from S2 nuclear (Nuc) or cytoplasmic (Cyto) fractions. U6 snRNA and 18S rRNA served as markers for the nuclear and cytoplasmic fractions, respectively. The enriched fraction is set to 1. (**b-h**) *Drosophila* S2 cells were treated with β-gal dsRNA (as a control) or two independent GW182 dsRNAs for 3 days. qRT-PCR was then performed to measure levels of the indicated circRNAs, β-actin RNA or U6 snRNA in S2 nuclear (Nuc) or cytoplasmic (Cyto) fractions. Data throughout the figure were normalized to the β-gal dsRNA sample and are shown as mean ± SD. n = 4. (**) $P < 0.01$; (*) $P < 0.05$.

**Figure S8. Effect of TNRC6A, TNRC6B or TNRC6C depletion on degradation of**

**endogenous linear RNAs and plasmid-derived circHIPK3 in human HeLa cells.**

(**a**) Domain structure of *Drosophila* GW182 and its human homolog (TNRC6C). Detailed sequence alignment is provided in Supplementary Protein Information. (**b**, **c**) Human HeLa cells were treated with a control siRNA or specific siRNAs to knock down TNRC6A, TNRC6B or TNRC6C for 2 days. qRT-PCR was then performed to assess the knock down efficiency (**b**) and measure levels of endogenous MALAT1, Hsp70, URH49 RNAs (**c**) or parental mRNAs of circRNAs (related to **Figure 1j**) (**d**). (**e**) Reporter plasmid that produce linear and circular RNA of HIPK3 (previously described in (Liang et al. 2014)) was transfected into HeLa cells treated with the indicated siRNAs. 30 µg of each sample was run on Northern Blots to examine the levels of plasmid-derived transcripts. Data throughout the figure were normalized to the control siRNA sample and are shown as mean ± SD. n = 3. (∗∗) $P < 0.01$; (∗) $P < 0.05$.

**Figure S9. Effect of TNRC6A, TNRC6B or TNRC6C depletion on degradation of circRNAs and their parental mRNAs in human HEK293 cells.**

(**a**) Human HEK293 cells were treated for 2 days with a control siRNA or specific siRNAs to knock down TNRC6A, TNRC6B or TNRC6C. (**b**-**c**) qRT-PCR was then performed to measure levels of the indicated human circRNAs (**b**) or their parental mRNAs (**c**) in HEK293 cells. Data throughout the figure were normalized to the control siRNA sample and are shown as mean ± SD. n = 3. (∗∗) $P < 0.01$; (∗) $P < 0.05$.

**Figure S10. Overexpression of GW182 mutants to identify the key domains of GW182 in circRNA degradation.**

(**a**) Domain structure of GW182 wild-type and mutants. GW182 mutants with deletions of ABD, UBA, Q-rich, Mid, RRM or C-term were generated. (**b**-**e**) qRT-PCR quantification of *Drosophila* endogenous circRNAs in RNA purified from S2 cells that were transfected with wild-type (WT) and the indicated mutants of GW182 for 2 days. Data were normalized to the empty vector (EV) sample and are shown as mean ± SD. n = 3. (∗∗) $P < 0.01$; (∗) $P < 0.05$.

**Figure S11. Overexpression of Mid domain alone decreases circRNA levels.**

(**a**) Domain structure of GW182 wild-type and mutants. (**b**) qRT-PCR quantification of *Drosophila* endogenous circRNAs in RNA purified from S2 cells that were transfected with wild-type (WT) and the indicated mutants of GW182 for 2 days. n = 4. (**c**) After GW182 expression plasmids transfection, qRT-PCR quantification of endogenous circHaspin was performed using RNA purified from S2 nuclear (Nuc) or cytoplasmic (Cyto) fractions. n = 3. Data throughout the figure were normalized to the empty vector (EV) sample and are shown as mean ± SD. (∗∗) $P < 0.01$; (∗) $P < 0.05$.

**Figure S12. Localization of GW182 and its Mid domain mutants.**

Wild-type (WT) or the indicated mutants of Flag-tagged GW182 were co-expressed with V5-tagged DCP2 (as a P-body marker) in *Drosophila* S2 cells for 2 days. Immunostainings for Flag-tagged GW182 (WT/ ΔMid/ Mid; Green) and V5-tagged

DCP2 (Red) were then performed to show the localization of GW182 and its mutants. Representative images are shown. White arrows indicate P-body. Scale bars, 5 μm.

# Supplementary Table S1

## *Drosophila* circRNA

| circRNA | genomic length | spliced length | exon count |
|---|---|---|---|
| CaMKI | 586 | 259 | 3 |
| Dbp80(Exon5/6) | 353 | 293 | 2 |
| Dbp80(Exon3/4) | 390 | 328 | 2 |
| uex | 334 | 334 | 1 |
| laccase2 | 490 | 490 | 1 |
| Haspin | 557 | 500 | 2 |
| CG42663 | 702 | 702 | 1 |
| ps | 811 | 811 | 1 |
| pan | 14846 | 846 | 7 |
| Nipped-B | 962 | 907 | 2 |
| mnb | 1096 | 1096 | 1 |
| dati | 1120 | 1120 | 1 |
| PlexA | 1439 | 1439 | 1 |
| Ect4 | 1542 | 1478 | 2 |

# Supplementary Table S2

## Human circRNA

| circRNA | genomic length | spliced length | exon count |
|---|---|---|---|
| UGP2 | 1631 | 236 | 2 |
| BBS9 | 4479 | 326 | 2 |
| PVT1 | 411 | 411 | 1 |
| NEIL3 | 7370 | 596 | 2 |
| ASH1L | 742 | 742 | 1 |
| PTBP3 | 46988 | 853 | 6 |
| SLAIN2 | 13936 | 971 | 5 |
| HIPK3 | 1099 | 1099 | 1 |
| NFATC3 | 4624 | 1298 | 2 |
| ANKRD17 | 7052 | 1832 | 2 |
| BPTF | 30550 | 2026 | 8 |
| ATXN1 | 2077 | 2077 | 1 |

# Supplementary Table S3

## dsRNA used in this paper

| dsRNA target | Forward Primer | Reverse Primer | DRSC |
|---|---|---|---|
| Dm_β-gal | TAATACGACTCACTATAGGG CTGGCGTAATAGCGAAGAGG | TAATACGACTCACTATAGGG CATTAAAGCGAGTGGCAACA | ------ |
| Dm_GW182(GW182) | TAATACGACTCACTATAGGG AATCCAAGTAATCCTATAAGCAG | TAATACGACTCACTATAGGG ATTGCTTGCTTTGCTTAATGA | DRSC17135 |
| Dm_GW182(GW182#2) | TAATACGACTCACTATAGGG CCCATAACCAGTTGCAAGGT | TAATACGACTCACTATAGGG ACATTGCTATTTCCAACGCC | DRSC38395 |
| Dm_twin | TAATACGACTCACTATAGGG TGTTGCTGAACAACAACTTTC | TAATACGACTCACTATAGGG CACACACATACTCAGGGATTT | DRSC15259 |
| Dm_Not1 | TAATACGACTCACTATAGGG GCGGTAGCGGATCTCG | TAATACGACTCACTATAGGG ATTATCTCGCTTAAATGGTCATA | DRSC06787 |
| Dm_Pop2 | TAATACGACTCACTATAGGG TCCAGCACTTGAATCGAAGA | TAATACGACTCACTATAGGG GACCGTGTAGGTTTCGGCTA | DRSC27971 |
| Dm_DCP1 | TAATACGACTCACTATAGGG GCGCCGTTAAATTCACAAAT | TAATACGACTCACTATAGGG TTCCCTGATCCTTGGACTTG | DRSC29844 |
| Dm_DCP2 | TAATACGACTCACTATAGGG AGACCGGGTTCGATATCAC | TAATACGACTCACTATAGGG TCCCGCTCCCGTTCCA | DRSC10597 |
| Dm_Ge-1 | TAATACGACTCACTATAGGG GAGTGGCAATACGGAGGAGA | TAATACGACTCACTATAGGG GTGAACTCCACTAGGCCCAA | DRSC28827 |
| Dm_Pat1 | TAATACGACTCACTATAGGG CCCAACGCACCAGAAATAC | TAATACGACTCACTATAGGG ACGATTCATCGTTTGGTATAAC | DRSC17750 |
| Dm_Edc3 | TAATACGACTCACTATAGGG GTTCGACAAGCAGGCAATTT | TAATACGACTCACTATAGGG CTCATATTTTTGCCGCCATC | DRSC23994 |
| Dm_me31B | TAATACGACTCACTATAGGG CACTCCCGGACGAATATTAG | TAATACGACTCACTATAGGG AGATTGCGACAGAGTCCTT | DRSC03569 |
| Dm_Upf1 | TAATACGACTCACTATAGGG GGAGAAGCCAGGCATTGA | TAATACGACTCACTATAGGG GACCGTGGCCCAACAGG | DRSC19667 |
| Dm_Upf2 | TAATACGACTCACTATAGGG CCACAGCAACTTCTCGATCA | TAATACGACTCACTATAGGG CTGTCTCAAATCGGGCAAAT | DRSC27785 |
| Dm_Upf3 | TAATACGACTCACTATAGGG CGGGACAAGAAAAAGGATGA | TAATACGACTCACTATAGGG CCTGTAAACATCAAGCGGGT | DRSC27316 |
| Dm_AGO1 | TAATACGACTCACTATAGGG ACCTGAAGATCACCTTCCCC | TAATACGACTCACTATAGGG CTGGATGATCTCCTGTCGGT | DRSC39159 |
| Dm_AGO2 | TAATACGACTCACTATAGGG AGGATGGAGCAACTCAGGT | TAATACGACTCACTATAGGG ATTCTAAACTGAGGAATAATCACA | DRSC10847 |
| Dm_AGO3 | TAATACGACTCACTATAGGG CTCGGTGGAACCAAGACATT | TAATACGACTCACTATAGGG CGTCGCAGCATAGCATTAAA | DRSC42716 |
| Dm_pcm | TAATACGACTCACTATAGGG TTCGGTACATGAAGACGCAG | TAATACGACTCACTATAGGG GCTCCTTGAAGTGATCCAGC | DRSC37724 |
| Dm_Dis3 | TAATACGACTCACTATAGGG ATCATCGTAACGATTGACACA | TAATACGACTCACTATAGGG CTTCATTGTCCACTTCCCAC | DRSC16034 |
| Dm_Csl4 | TAATACGACTCACTATAGGG CAGGTGGTTAGTGTGCATAA | TAATACGACTCACTATAGGG TTTTAGTTCCACAAATGAATTTAAG | DRSC22664 |
| Dm_Mtr3 | TAATACGACTCACTATAGGG CTATGTGAATTTTGCGGCCT | TAATACGACTCACTATAGGG CAGAGCAGCTCCTCAACCTT | DRSC26956 |
| Dm_Rrp4 | TAATACGACTCACTATAGGG TTTGTTTACCTCGCTTTGGC | TAATACGACTCACTATAGGG CATAGCGACTCTTAAGCGGG | DRSC39222 |
| Dm_Rrp6 | TAATACGACTCACTATAGGG TTATCGTTGTATATCGTCAACAT | TAATACGACTCACTATAGGG GCATCTCCCTTGGAAGACT | DRSC16223 |
| Dm_Rrp40 | TAATACGACTCACTATAGGG CAGCCTCCATATCGTATCTC | TAATACGACTCACTATAGGG CGAGTTGACGCAGACCA | DRSC00074 |
| Dm_Rrp41 | TAATACGACTCACTATAGGG TACATGGAGCAGGGAAACAC | TAATACGACTCACTATAGGG CAAGTGATCGATGTGGAACC | DRSC02491 |
| Dm_zucchini | TAATACGACTCACTATAGGG AGCAAGCGAGAGAAGGC | TAATACGACTCACTATAGGG CCAAGAGCCGTCCAGTTT | DRSC02160 |
| Dm_CG3303 | TAATACGACTCACTATAGGG GAAGATAAACGGCGAGCATC | TAATACGACTCACTATAGGG ACCTGAAGTGGTCTTCCCCT | DRSC26161 |
| Dm_Rpp30 | TAATACGACTCACTATAGGG ATGGGGACTTGATAACTTTTGA | TAATACGACTCACTATAGGG ATTAGCAACGTCGTAAGGTC | DRSC00320 |
| Dm_Smg6 | TAATACGACTCACTATAGGG GGACGCTTTCAAGCAACTTC | TAATACGACTCACTATAGGG CTCTGATAGTACCTGGCCGC | DRSC27703 |

## siRNA used in this paper

| siRNA target | siRNA sequence |
|---|---|
| Control | UUCUCCGAACGUGUCACGUUU |
| hu_TNRC6A | GCCUAAUCUCCGUGCUCAAUU |
| hu_TNRC6B | GGCCUUGUAUUGCCAGCAAUU |
| hu_TNRC6C | GCAUUAAGUGCUAAACAAAUU |

# Supplementary Table S4

## qRT-PCR primers for *Drosophila* genes

| Gene Name | Forward Primer | Reverse Primer |
|---|---|---|
| circDbp80(5/6)(qPCR) | AGCCACCCAAGGTCATCATG | AATTGTCCCATTCGCGCAGC |
| circDbp80(3/4)(qPCR) | GCCATGCTTAGCCGAGTCAAC | GCTTTGTGTTCACCAGCCCC |
| circlaccase2(qPCR) | GCCTCCGAGAATTTGCTACTATCA | ACATGTTGCTGCCAGAAGGAC |
| circHaspin(qPCR) | CGATGTCTATCGAATGATGCGGA | CCGGATGGTCATTTTCAGATCCC |
| circCaMKI(qPCR) | GGGTCCTACACAGAAAAGGACG | CATTTGCCATCGAAATGATTTGCA |
| circCG42663(qPCR) | AGAAGCCAAAATGTTCGCGG | ATTTGCGCTCCACGTTGACA |
| circps(qPCR) | TCCGATCCGGAAAATGAACAA | GACAGTTCGTGTACGTCGCT |
| circNipped-B(qPCR) | AAAGGTTTGGCTTTCAGGAAGT | TCAAAATGTCGCCTAAGATACTGT |
| circmnb(qPCR) | GAGGATACGAATAGCGGCGG | AATTGTCGCCACGCTTTGTT |
| circdati(qPCR) | GGTGCCAACTGTGCGAAGTT | CGACGCCCGACATCAAATACAAT |
| circpan(qPCR) | AAACAAGAATGCGGTGTTCAGG | CTCGAAAAACTTCTTTGCACTGCA |
| circPlexA(qPCR) | ATTTCCTTTGCGTTGGTGTCATT | CCCAGCATGCCATGTGTTCTA |
| circEtc4(qPCR) | TTCAGCCTCAGTCCTTCGAG | TTTTCGTGACTTTGCGTGGG |
| circuex(qPCR) | ATTCGCAATGGTTCGCCGTG | GCAACGGATTTTCAGCACTTTACT |
| β-actin(qPCR) | CTCCGTCCACCATGAAGATT | TTCGAGATCCACATCTGCTG |
| rp49(qPCR) | TACAGGCCCAAGATCGTGAAG | GACGCACTCTGTTGTCGATACC |
| 18S(qPCR) | GTGCTGAAGCTTATGTAGCCT | TGGGACAAACCAACAGGTACG |
| U6(qPCR) | GTTCTTGCTTCGGCAGAACATATACT | TGTGGAACGCTTCACGATTTTGC |
| U7(qPCR) | GAAATTTGTCTTGGTGGG | AACGGGAACACTCAATG |
| Hsp70(qPCR) | CAAGAACCTCAAGGGTGAGC | GCCGGTTGTCAAAGTCCTC |
| Hsp83(qPCR) | CATTCCAGGCTGAGATTGCTC | ATCGGAAGCGTTCGAGATCA |
| GW182(qPCR) | TCACCGACTGATTTACCGCC | TGTCCAAAGCTCCGAAGTGG |
| twin/CCR4(qPCR) | TGAACTGAGCCAGGCAATCA | CAGCTATGTCCTCCTGCGAC |
| Not1(qPCR) | ACCAAAGCAACCGGAAGGAA | CGCACGTACAGCGAAAACAG |
| Pop2(qPCR) | ACAGAGCCAGTGACCCAGAA | TCGCGGGCATTACAGCTAAA |
| DCP1(qPCR) | CAGCCACTGCTGAACTCGAC | GGAACAGCCGTTCAGGTAGG |
| DCP2(qPCR) | GATCAATAACGCCGTCGCTG | GACGCGAGCAACGCTAGATT |
| Ge-1(qPCR) | GGTGTTGTCTTGCCTGGACT | GCAGCCTTCTCCTTCGCTAT |
| Pat1(qPCR) | GGATCGCTCAACGGAGTTCA | TCGAGTTCCTCCTGTGTTGC |
| Edc3(qPCR) | TGCGTGGTGCGTAGTTAGTT | GTTCTGCCCTGGCTAATGGT |
| me31B(qPCR) | CTGATGTGACCGATACGCGA | CTTCCTGAATTGGCGAAGGG |
| Upf1(qPCR) | CGCTTACCTGGTGCAGTACA | AGATCGCACGCAAGACATGA |
| Upf2(qPCR) | ACACCTCCGATTTGTTCCCC | GATGATCAATCGCCGCCTTG |
| Upf3(qPCR) | CACAAGGGCGTCGAGTACAT | GGTTCGCTCTCGATGGTGTT |
| AGO1(qPCR) | GGACATTCGCGACATCAACG | GCACACACGATACTTGCGAC |
| AGO2(qPCR) | CAGAGCCAAGGCCAATACCA | AGTTGATGCCTACTTGCCCG |
| AGO3(qPCR) | TCGGAGGCTCATTATGGACG | CAGCAACAGAATTCCCCCGA |
| GW182(qPCR) | TCACCGACTGATTTACCGCC | TGTCCAAAGCTCCGAAGTGG |
| pcm(qPCR) | TGTGCTAAAACCACGCAAGC | TATCGCCTTGACGAGATGCG |
| Dis3(qPCR) | ACAGGAGCTCCGCCATTTAC | TTGGCGGTCTCATCTGGTTC |
| Csl4(qPCR) | GAGACTGTAGTCTGCCTGCC | AATTCCAGACTTGGAGGCGT |
| Mtr3(qPCR) | CGTAAGTGGAAATGCCGCAC | TTCAGCTGGACATACGGGTG |
| Rrp4(qPCR) | GCCAAGTGCAAGCTGATGAT | GTCGTAGATGGCGTTCTGCT |
| Rrp6(qPCR) | AGGAGTGCAACGACGTACTG | GGGCTCTATGGATGTGGAGC |
| Rrp40(qPCR) | ATAGTTATGCCCGGTGAGCG | CGGGTTCCTTGTGTCGAAGT |
| Rrp41(qPCR) | CGAGACTGCTATGGCGGGTT | TTTCGCAAAGTCAGAGGCACT |
| Smg6(qPCR) | GATAATTGGGACGCTGCCGA | TCAGCGTTTTCCGTCTCCAA |
| zucchini(qPCR) | ACCAAACAGTTCCTCAGTATGC | GGCACTCCTTTGTGTGTGAAG |
| CG3303(qPCR) | GCAAAAGGCTAACCTCCAAGC | GTCCTGACTGACATCCTCGTC |
| Rpp30(qPCR) | TGAGGACGAGAAAAGCACCG | AATAGCGTGCTCAGTTCCGT |
| linearCaMKI(qPCR) | TGGAGCGTGGACTTCTAACG | ACTGCGATGCTGCATTTGTG |
| linearDbp80(qPCR) | AGGAATCACTTGAAAACATAAAGCA | GACCAACGCTTATGCAACCG |
| linearuex (qPCR) | TGTGAAGGTCCTCCAACTCAG | ACGAAGCCATCCAAGACGAC |
| linearlaccase2(qPCR) | ACGCAACGAATACGGTTTGG | CACACTTGAATGCTTGGCCC |
| linearHaspin(qPCR) | ACTGGTCATCGTTTTCACCCA | TCCTGTGAACTTTCGTATTGATGC |
| linearCG42663(qPCR) | TGCTCGATAGTATGCCACCAC | TTCCCGCCTCCGATTCAAC |
| linearEct4(qPCR) | GAGTGCACAAGGGAAAACCC | TGGTCGATGTGACAACCATTG |
| lineardati(qPCR) | CGACGCCCGACATCAAATACA | AACATTCTTCGAGGGTGCGT |

# Supplementary Table S5

## qRT-PCR primers for human genes

| Gene name+A2:C37 | Forward Primer | Reverse Primer |
|---|---|---|
| huHsp70(qPCR) | ATGTCGGTGGTGGGCATAGA | CACAGCGACGTAGCAGCTCT |
| huMALAT1(qPCR) | GTCATAACCAGCCTGGCAGT | GCTTATTCCCCAATGGAGGT |
| hu18S(qPCR) | CTCAACACGGGAAACCTCAC | CGCTCCACCAACTAAGAACG |
| huUAP56(qPCR) | GACAGCAGCTGGGGGAGATG | CTCATGCTGGACTTCTGACG |
| huURH49(qPCR) | GCCCCAGGCTCCTCAAGAGA | CTCATGCTGGACCTCAGAAG |
| huTNRC6A(qPCR) | TGGAAGCTAAAGCTACCAAAGACG | TTCAGTGGCCTTCTTTTGAGCAG |
| huTNRC6B(qPCR) | AGGAGCAAGAAAGGGAAGAACAGTT | CCGTTGGTTGGCTTAAACTTGGT |
| huTNRC6C(qPCR) | GCTACAGGGAGTGCCCAGG | CTGCTCCAAGGGCACTCTGA |
| huGAPDH(qPCR) | CTTCATTGACCTCAACTACATGG | CTCGCTCCTGGAAGATGGTGAT |
| hucircMYH11(qPCR) | GCGAGGTGAACGCACTCAA | GCTGGGACTCCTCCTCTGC |
| hucircUGP2(qPCR) | CTTCTGTGGATTGGGGAAAA | TGTGATGATGCTGTGGTGAG |
| hucircBBS9(qPCR) | CGCCGGCTACTAGCAAGATT | CTTGAAGGCGAAGAATAAGCTC |
| hucircC3(qPCR) | GGAAGTCCCTGAAGGTCGTG | TTTCCACCTGCTCGTTTCG |
| hucircPVT1(qPCR) | TTCAGCACTCTGGACGGACTT | TATGGCATGGGCAGGGTAG |
| hucircNEIL3(qPCR) | TGCATTCTCCGAGTTGTGGG | CACGGGTACTTCATTAAGTGGCTA |
| hucircASH1L(qPCR) | TTTCTTTAATTCTCTTGGACCC | ACCCTCATCACCAGCCTTG |
| hucircPTBP3(qPCR) | CCTTGAACCCCCTATGGCTG | TCCCATTAGCATACACACCTG |
| hucircSLAIN2(qPCR) | TCAAGTGCCAAACGGAGGAA | ATCCAAAACTTGCCTGCACC |
| hucircHIPK3(qPCR) | TATGTTGGTGGATCCTGTTCGGCA | TGGTGGGTAGACCAAGACTTGTGA |
| hucircNFATC3(qPCR) | AACTCATCATCGAGCCCATT | TGGTAAGCAAAGTGGTGTGG |
| hucircANKRD17(qPCR) | CAACATTTTTCCCCGCTTAG | CAATTTCTTTGTCTGGATCCTTG |
| hucircBPTF(qPCR) | TCCAAGTGACTCCCCATTTT | GTACCTGCATCTGGGGTGAC |
| hucircATXN1(qPCR) | GATTGAAGACAGCCATAGCC | CTGATAAACGGAAAGTCACATT |
| hulinearASH1L(qPCR) | TTCTTCTTTGGGGGACGTGG | CCCAGTCCTTCTGAAACCCC |
| hulinearHIPK3(qPCR) | TGGAGACTGGGGGAAGATGA | CACACTAACTGGCTGAGGGG |
| hulinearUGP2(qPCR) | GGTGGTGAAACTCAATGGTGG | TGCTGAACAGTCAGATCCAGA |
| hulinearBBS9(qPCR) | GCATTCCGCGAGTTATCCAA | TCAATAGTAATTTTGTGGCTTGCAG |
| hulinearNEIL3(qPCR) | AAACAGAAAGGCCGGATGCT | GGGTGGAGACCACTGTCAAA |
| hulinearPTBP3(qPCR) | GCCCGGCTTTTTAAAGCGAT | CACTGTCCAAACATCAAGCAGT |
| hulinearSLAIN2(qPCR) | CAGAGGCCAATATCTGGGCAA | AGCCTGAATTCACCATTTTCTTGT |
| hulinearNFATC3(qPCR) | GCAGGTATTTTGAAACTCCGCA | TGGGATGTGTACACGAAACAC |
| hulinearANKRD17(qPCR) | ATGGCTGCTTTTTGCTGCTC | TCCAGCTGCAGCGATGAAA |
| hulinearBPTF(qPCR) | AGCAGAAGCCGACAGTGATT | AACTGAGCCACTTATGGGGG |
| hulinearATXN1(qPCR) | GAGAGCGAACACCCACGCT | CACCCGAGTTGTCCATAGTCATGAA |
| hulinearPVT1(qPCR) | TCCTGTTACACCTGGGATTTAGG | AGCTTTAGGTCACGTAAGGACA |

## For Metabolic labeling of nascent RNA

**Synthetic RNA sequence:**

5’-AUUUAGGUGACACUAUAGGAUCCUCUAGAGUCGACCUUCUCCCUAUAGUGAGUCGUAUUAGCA[4-S-U]CAG-3’

| Name | Forward Primer | Reverse Primer |
|---|---|---|
| Synthetic RNA(qPCR) | ATTTAGGTGACACTATAGGATCCTCTAG | GCTAATACGACTCACTATAGGGAGAAG |

**Supplementary Protein Information**

Protein sequence alignment
(Dm Gw182, Hs TNRC6A, Hs TNRC6B, Hs TNRC6C)

```
GW182     1 MREALFSQ-------------------------------DGWGCQHVNQDTNWEVPSSPE
TNRC6A    1 MRELEAKATKDVERNLSRDLVQEEEQLMEEKKKKKDDKKKKEAAQKKATEQKIKVPEQIK
TNRC6B    1 MREKEQER---------------EEQLMEDKKRKKEDKKKKEATQK-VTEQKTKVPEVTK
TNRC6C    1 MATGSAQG------NFT-------------------------------------------

GW182    30 PANKD-----APGPPMWKPSINNGTDLWESNLRNGGQ-----------------------
TNRC6A   61 PSVSQPQPANSNNGTSTA--TSTNNNAKRATANNQQPQQQQQQQQPQQQQPQQQPQPQPQ
TNRC6B   45 PSLSQPTAASP-IGSSPSPPVNGGNNAKRVAVPNGQP-----------------------
TNRC6C   12 ---GHTK----------K--TN--------------------------------------

GW182    62 -------PAAQ-QVPK--------------------------------------------
TNRC6A  119 QQQPQQQPQALPRYPREVPPRFRH-QEHKQLLKRGQHFPVIAANLGSAVKVLNSQSESSA
TNRC6B   81 -------PSAARYMPREVPPRFRCQQDHKVLLKRGQPPPPSCMLLGGGAGPPPCTAPGAN
TNRC6C   19 ------------------------------------------------------------

GW182    70 ------------------------------------------------------------
TNRC6A  178 LTNQQPQ-NNGE-VQNSKNQSD-INHST-SGSHYENSQRGPVSSTS----DSSTNCKNAV
TNRC6B  134 --PNNAQVT-GALLQSESGTAPDSTLGGAAASNYANSTWGSGASSNNGTSPNPIHIWDKV
TNRC6C   19 --GNNGT-N-GALVQSPSNQSA-LGAGGANSNGSAARVWGVATGSS--------------

GW182    70 -------PSWGHTPSSNLG---------------------------GTWGEDDDGADS--
TNRC6A  230 VSDLSEKEAWPSAPGSDPELASEC-MDADSASSSESERNITIMASGNTGGEKDGLRNSTG
TNRC6B  191 IVDGSDMEEWPCIASKDTESSSENTTDNNSASNPGSEK----------------------
TNRC6C   60 -----------------SG-----------------------------------------

GW182    94 ----SSVWTGGAVSNAGSGAAV--------------------------------------
TNRC6A  289 LGSQNKFVVGSSSNNVGHGSST-----------GPWGFSHGAIISTCQVSVDAPESKSES
TNRC6B  229 -----STLPGSTTSNKGKGSQCQSASSGNECNLGVWKSDPK----A--KSVQSSNSTTEN
TNRC6C   62 ---------------------------------------------LAHCSVSGGDGKMDT

GW182   112 ------------------------------------------------------------
TNRC6A  338 SN---NRMNAWGTVSSSSN--GGLNPSTLNSASNHGAWPVLENNGLALKGPVGSGSSGIN
TNRC6B  278 N----NGLGNWRNVSGQDRIGPGSGFSNFNPNSNPSAWPALVQEGTSRKGALETDNSNSS
TNRC6C   77 MIGDGRSQNCWGA--SNSN--AGIN-LNLNPNANPAAWPVLGHEGTVATGNP----SSIC

GW182   112 ------------------------------------------------------------
TNRC6A  393 IQCSTIGQMPNNQSINSKVSGGSTHGTWGSL--QETCESEVSG-TQKVSFSGQPQNITTE
TNRC6B  334 AQVSTVGQTSREQQSK----------------------MENAGVNFVVSGREQAQIHNT-
TNRC6C  128 SPVSAIGQNMGNQNGNPT----GTLGAWGNLLPQESTEPQTST-SQNVSFSAQPQNLNT-

GW182   112 ------------------------------------------------------------
TNRC6A  450 MTGPNNT-TNFMTSSLPNS-GSVQNNELPSSNTGAWRVSTMNHPQMQAPSGMNGT-SLSH
TNRC6B  371 -DGPKNGNTNSLNLSSPNP---MENKGMPF-GMGLG-NTS------------RSTDAPSQ
TNRC6C  182 -DGPNNT--NPMNSS-PNPINAMQTNGLPNWGMAVG-MGAIIPPHLQGLPGANGS-SVSQ
```

```
GW182   112 ---------------------------------------------------------GVN
TNRC6A  507 LSNGESKSG--GSYGTTWGAYG-SNYSGDKCSGPNGQANGDT----VNATLMQPGVNGPM
TNRC6B  413 -STGDRKTGSVGSWGAARGPSGTDTVSGQSNSGNN-GNNGKEREDSWKGASVQK-STGSK
TNRC6C  236 VSGGS-AE---GISNSVWGLSPGNPATGNSNSGFS-QGNGDT----VNSALSAK-QNGSS

GW182   115 Q--AGVNVGPGGVVSSGGPQWGQGVVGVGLGSTGGNGSSNITGSSGVATGSSGNSSNAGN
TNRC6A  560 GTNFQVNTNKGGGVWESGAA-NSQSTSWGSGN-GANSG--G----------------SRR
TNRC6B  470 NDSWDNNNRSTGGSWNFGPQ-DSNDNKWGEGN-KMTSG--V----------------SQG
TNRC6C  286 S--AVQKEGSGGNAWDSGPPAGPGILAWGRGS-GNNGVGNI----------------HSG

GW182   173 GWGDPREIRPL-------------------------------------------------
TNRC6A  600 GWGTPAQNTGTNLPSVEWNKLPSNQHSNDSANGNGKTFTNGWKSTEEEDQGSATSQTNEQ
TNRC6B  510 EWKQPTGSDE--LKIGEWSG-PNQPNSST----------GAWDNQK--GHPLPENQGNAQ
TNRC6C  327 AWGHPSRSTSNGV-NGEWGK-PPNQHSNSDINGKGS---TGWESPSVTSQNPTVQPGGEH

GW182   184 -------------GVGGSMDIRNVEHRGGN-GSGATSSDPRDIRMI-----DPR------
TNRC6A  660 SSVWAKTGG--TVESDGSTESTGRLE-----EKGTGESQSRDRRKI-----DQHTLLQSI
TNRC6B  555 APCWGRSSS--STGSEVGGQSTGSNH-----KAGSSDSHNSGRRSYRPTHPDCQAVLQTL
TNRC6C  382 MNSWAKAASSGTTASEGSSDGSGNHNEGSTGREGTGEGRRRDKGII-----DQG-HIQ--

GW182   219 -----------------DP--------IRGDPRGISGRLNGTSEMWGHHPQMSHNQLQG
TNRC6A  708 VNRTDLDPRVLSNSGWGQTPIKQNTAWDTETSPRGERKTDNGT-EAWGSSATQTFNSGAC
TNRC6B  608 LSRTDLDPRVLSNTGWGQTQIKQDTVWDIEEVPRPEGKSDKGT-EGWESAATQTKNSGGW
TNRC6C  434 LPRNDLDPRVLSNTGWGQTPVKQNTAWEFEESPRSERKNDNGT-EAWGCAATQASNSGGK

GW182   253 INKMVGQSVATAS-----------------------------------------------
TNRC6A  767 IDKTSPNGNDTSSVSGWGDPKPAL-----RWGDSK--GSNCQGGWEDDSAATGMV-----
TNRC6B  667 GDAPSQS---NQMKSGWGELSAST-----EWKDPK-----NTGGWNDYKNN---------
TNRC6C  493 NDGSIMNSTNTSSVSGWVNAPPAAVPANTGWGDSNNKAPSGPGVWGDSISSTAVSTAAAA

GW182   266 ------------------------------------------------------------
TNRC6A  815 -KSNQWGNC---KEEKAAWNDSQK-NKQGWGDGQKSSQGWSVSASDNWGETSR-NNHWGE
TNRC6B  705 -NSSNWGGGRPDEKTPSSWNENPS-KDQGWGGGRQPNQGWSSGKN-GWGEEV-------D
TNRC6C  553 KSGHAWSGAANQEDKSPTWGEPPKPKSQHWGDGQRSNPAWSAGGG-DWADSSSVLGHLGD

GW182   266 ------------------------TSVGTSGSGIGP--GGP---------GPS-TVSGNI
TNRC6A  869 ANKKSSSGGSDSDRSVSGWNELGKTSSFTWGNNINP--NNSSGWDESSKPTPS-QGWGDP
TNRC6B  755 -QTKNSNWESSASKPVSGWGEGGQNEIGTWGNGGNASLASKGGWEDCKR-SPAWNETGRQ
TNRC6C  612 GKKNGSGWDADSNRSGSGWNDTTRSGNSGWGNSTNTKANPGTNWGETLKPGPQ-QNWASK

GW182   290 ------------------------------PTQWGPA-----Q---PV-SVGVSGPKD--
TNRC6A  926 PKSNQSLGWGD---SSKPVSSPDWNKQQDIVGSWGIPP----ATGKPPGTGWLGGPIPAP
TNRC6B  813 PNS-----WNKQHQQQQPPQQPP-PPQPEASGSWGGPPPPPPGNVRPSNSSWSSGPQPAT
TNRC6C  671 PQ-------------------------DNNVSNWGGA-----ASVKQTGTGWIGGPVPVK

GW182   309 -MS-KQISGWEEPSPPPQRRSIPNYDDGTSLWGQQTRVPAASGHWKDMTDSIGRSSHLMR
TNRC6A  979 AKE-EEPTGWEEPSPESIRRKM-EIDDGTSAWGDPSKYNYKNVNMWNKNVPNGNSRSDQQ
TNRC6B  867 PKD-EEPSGWEEPSPQSISRKM-DIDDGTSAWGDPNSYNYKNVNLWDKNSQGGPAPREPN
TNRC6C  701 QKDSSEATGWEEPSPPSIRRKM-EIDDGTSAWGDPSNYNNKTVNMWDRNNPVIQSSTTTN
```

```
GW182   367 GQSQTGGIGIAGVGNSNVPVGANPSNPISSVVGPQARIPSVG---GVQHKPDGGAMWVHS
TNRC6A 1037 AQVHQL--------------------------LTPASAISNK------------------
TNRC6B  925 LPTP--------------------------------------------------------
TNRC6C  760 TTTTTTTTTSNT---------------THRVETPPPHQAGTQLNRS-PLLGPVSSGWG--

GW182   424 GNVGGRNNVAAVTTWGDDTHSVNVGAPSSGSVSSNNWVDDKSNSTLAQN---SWSDPAPV
TNRC6A 1053 --------------------------EASSGSGWGEPWGEPSTPATTVDNGTSAWGKPIDS
TNRC6B  929 ----------------------------MTSKSASVWSKSTPPA--PDNGTSAWGEPNES
TNRC6C  802 ----------------E--------MPNVHSKTENSWGEPSSPSTLVDNGTAAWGKPPSS

GW182   481 GVSWGNKQ------------------SKPPSNSASSGWSTAAGVVDGVDL---------
TNRC6A 1088 GPSWGEPIAAASSTSTWGSSSVGPQALSKSGPKSMQDGWCGDDMPLPGNRPTGWEEEEDV
TNRC6B  959 SPGWGEMDDTGASTTGWGNTPANAPNAMKPNSKSMQDGWGESDGPVTGARHPSWEEEED-
TNRC6C  838 GSGWGDHPAEPPVAFGRAGAPVAASALCKPASKSMQEGWGSGG-DEMNLSTSQWEDEE--

GW182   513 -GSEWNTHGG--------------------IIGKSQQQ--QKLAGLNVGMVNVINAEIIK
TNRC6A 1148 EIGMWNSNSSQELNSSLNWPPYTKKMSSKGLSGKKRRRERGMMKGGNK------QEEAWI
TNRC6B 1018 -GGVWNTTGSQGSASSHN-------SASWGQGGKKQMK--CSLKGGN--------NDSWM
TNRC6C  895 -GDVWNNAASQESTSSC---------SSWGNAPKKGLQ--KGMKTSGK------QDEAWI

GW182   550 QSKQYRILVENGFKKEDVERALVIANMNIEEAADMLRANSSLSMDGWRRHDESLGSYADH
TNRC6A 1202 -NPFVKQFSNISFSRDSPEENVQSNKMDLSGG--MLQDK-RME------IDKHSLNIGDY
TNRC6B 1060 -NPLAKQFSNMGLLSQT--EDNPSSKMDLSVG--SLSDK-KFD------VDKRAMNLGDF
TNRC6C  937 MSRLIKQLTDMGFPREPAEEALKSNNMNLDQAMSALLEK-KVD------VDKRGLGVTDH

GW182   610 NSSTSSGGFAGRYPVNSGQPS-MSFPHNNL--------MNNMGGTAVTGGNNNTNMTALQ
TNRC6A 1252 NRTVGKG--PGSRPQISKESS-ME--RNPY--------FDKDGIVA----DESQNMQFMS
TNRC6B 1108 NDIMRKD-RSGFRPPNSKDMGTTD--SGPYFEKLTLPFSNQDGCLG----DEAPCSPFSP
TNRC6C  990 NGMAAKP--LGCRPPISKESS-VD--RPTF--------LDKDGGLV----EEPTPSPFLP

GW182   661 VQKYLNQGQHGVAVGPQAVGNSSAVSVGFGQ-NTSNAAV--AGAASVNIAANT-NNQPSG
TNRC6A 1295 SQ-SMKLPPSNSALPNQALGSIA----GLGMQNLNSVRQ--NGNPSMFGVGNT-AAQPRG
TNRC6B 1161 SP-SYKLSPSGSTLPNVSLGAIG---TGLNPQNFA-ARQ--GGSHGLFG--NS-TAQSRG
TNRC6C 1033 SP-SLKLPLSHSALPSQALGGIA---SGLGMQNLNSSRQIPSGNLGMFG--NSGAAQART

GW182   717 QQI------R--------------------MLGQQIQLAIHSGFISSQILT--QPLTQT
TNRC6A 1347 MQQP---PAQPLSSSQPNLRAQVPPPLLSPQVPVSLLKYAPNNGGLNPL-------FGPQ
TNRC6B 1211 LHT----PVQPLNSS-PSLRAQVPPQFISPQVSASMLKQFPN-SGLSPGLFNVGPQLSPQ
TNRC6C 1087 MQQPPQPPVQPLNSSQPSLRAQVP-QFLSPQVQAQLLQFAAKNIGLNPALLT--SPINPQ

GW182   748 TLNLLNQLLS--------NIKHL---QAAQQSLTRGGNVN-------PMAVNVAISKYKQ
TNRC6A 1397 QVAMLNQLSQLNQLSQISQLQRL---LAQQQRAQSQRSVPSGNRPQ-QDQQGRPLS-VQQ
TNRC6B 1265 QIAMLSQLPQIPQFQL--ACQLLLQQQQQQQLLQNQRKISQAVRQQQEQQLARMVSALQQ
TNRC6C 1144 HMTMLNQLYQL---QL--AYQRL---QIQQQMLQAQRNVSGSMRQQ-EQQVARTITNLQQ

GW182   790 QIQNLQNQINAQQAVYVKQQNMQPTSQQQQPQQQQLPSVHLS----NS---GNDYLRGHD
TNRC6A 1452 QMMQQSRQL--DPNLLVKQQTP--PSQQQPLHQP-AMKSFLDNVMPHT----TPELQK-G
TNRC6B 1323 QQQQQQRQ-------------P--GMKHSPSHPV-GPKPHLDNMVPNALNVGLPDLQTKG
TNRC6C 1195 QIQQHQRQL--AQALLVKQPPPPPPPPHLSLHPS-AGKSAMDSFPSHPQTPGLPDLQTKE
```

```
GW182   843 ------------AINNLQSNFSELNINK--PSGYQGASNQQSRLNQWKLPVLDKEINSDST
TNRC6A 1502 -PSPINAFSNFPI-GLNSNLNVN-MDM-------NSIKEPQSRLRKWTTVD---SIS-VNT
TNRC6B 1367 ------------PIPGYGSGFSSGGMDY--GMVGGKEAGTESRFKQWTSMM--EGLPSVAT
TNRC6C 1252 QQSSPNTFAPYPLAGLNPNMNVNSMDMTGGLSVKDPSQSQSRLPQWTHPNSMDNLPSAAS

GW182   890 ---EFSRAPGATKQNLTA-------NTSNINSLGLQNDST--WSTGRSIGDGWPDPSSDN
TNRC6A 1549 SLDQNSSKHGAISSGFRL--------EESPFVPYDFMNSSTSPASPPGSIGDGWPRAKSPN
TNRC6B 1412 -QEANMHKNGAIVA------PGKTRGGSPYNQFDIIPGDTL-GGHTGPAGDSWLPAKSPP
TNRC6C 1312 PLEQNPSKHGAIPGGLSIGPPGKSSIDDSYGRYDLIQNSESPASPPVAVPHSWSRAKSDS

GW182   938 ENKDWSVAQPTSAATAYTDLVQEFEPGKPWKGSQIKSIEDDPSITPGSVARSPLSINSTP
TNRC6A 1602 -------------GSSSVNWPPEFRPGEPWKGYPNIDPETDPYVTPGSVINNL-SIN-TV
TNRC6B 1464 TNKI-------GSKSSNASWPPEFQPGVPWKGIQNIDPESDPYVTPGSVLGGT-ATS-PI
TNRC6C 1372 D-KI--------SNGSSINWPPEFHPGVPWKGLQNIDPENDPDVTPGSVPTGP-TINTTI

GW182   998 KDADIF--ANTGKNSPTDLPPLSLSSSTWSF-NPNQNY------------------PSH
TNRC6A 1647 REVDHL--RDRNSGSSSSLNTTLPSTSAWSSIRA-SNYNVPLSST--AQSTSARNSDSKL
TNRC6B 1515 VDTDHQLLRDNTTGSNSSLNTSLPSPGAWPY-SA-SDN--SFT-N--VHSTSAKFPDYKS
TNRC6C 1422 QDVNRYLLKSGGSSPPSSQNATLPSSSAWPL-SA-SGYSSSFSSIASAPSVAGKLSDIKS

GW182  1036 SWSDNSQ---QCTATSELWTSP---LNKSSSRGPPPGLTANSNKSANSNASTPTTITGGA
TNRC6A 1702 TWSPGSV--TNTSLAHELWKVPLPPKNITAPSRPPPGLTGQKPPLSTWD-NSPLRI---G
TNRC6B 1568 TWSPDPIGHNPTHLSNKMWKNHISSRNTTPLPRPPPGLTNPKP-SSPWS-STAPRS---V
TNRC6C 1480 TWSSGPTSHTQASLSHELWKVP---RNSTAPTRPPPGLTNPKP-SSTWG-ASPL------

GW182  1090 NGWLQPRSGGVQTTNTNWTGGNTTWGSSWLLLKNLTAQIDGPTLRTLCMQHGPLVSFHPY
TNRC6A 1756 GGWGNS--DARYTPGSSWGESSSGRITNWLVLKNLTPQIDGSTLRTLCMQHGPLITFHLN
TNRC6B 1623 RGWGTQ--DSRLASASTWSDGGSVRPSYWLVLHNLTPQIDGSTLRTICMQHGPLLTFHLN
TNRC6C 1529 -GWTS-----SYSSGSAWSTDTSGRTSSWLVLRNLTPQIDGSTLRTLCLQHGPLITFHLN

GW182  1150 LNQGIALCKYTTREEANKAQMALNNCVLANTTIFAESPSENEVQSIM-QHLPQTP-----
TNRC6A 1814 LPHGNALVRYSSKEEVVKAQKSLHMCVLGNTTILAEFASEEEISRFFAQSQSLTPS----
TNRC6B 1681 LTQGTALIRYSTKQEAAKAQTALHMCVLGNTTILAEFATDDEVSRFLAQAQPPTPAATPS
TNRC6C 1583 LTQGNAVVRYSSKEEAAKAQKSLHMCVLGNTTILAEFAGEEEVNRFLAQGQALPPT----

GW182  1204 ----SSTSSSGTSGGNVGGVGTS------ANNANSGSAACLSGNNSGNGNGSASGAGSGN
TNRC6A 1870 ---PGWQS-LGSSQSRLGSLDCSHS-FSSRTDLNHWNGAGLSGTNCGDLHGTSL-------
TNRC6B 1741 APAAGWQS-LETGQNQSDPVGPALNLFGGSTGLGQWSSSA-GGSSGADLAGASL-------
TNRC6C 1639 ---SSWQSSSASSQPRLSAAGSSHG-L-VRSDAGHWNAPCLGGKGSSEL----L-------

GW182  1254 NGNSSCNNSA-AGGGSSSNNTITTVANSNLVGSSGSVSNSSGVTANSSTVSVVSCTASGN
TNRC6A 1919 ---------WG-TPHYSTSLWGPPSSSDPRGISSPSPI------------NAFL----SV-
TNRC6B 1793 ---------WG-PPNYSSSLWGVPTVEDPHRMGSPAPL---------------L----PG-
TNRC6C 1684 ---------WGGVPQYSSSLWGPPSADDSRVIGSPTPL------------TTLL----PG-

GW182  1313 SINGAGTANSSGSKSSANNLASGQSSASNLTNSTNSTWRQTSQNQALQSQSRPSGREADF
TNRC6A 1953 -------------------DHLGGG----GE-----SM-----------------------
TNRC6B 1824 -------------------DLLGGG----SD-----SI-----------------------
TNRC6C 1719 -------------------DLLSG------E-----SL-----------------------
```

```
GW182  1373 DYISLVYSIVDD
TNRC6A     ------------
TNRC6B     ------------
TNRC6C     ------------
```

## Supplementary Plasmid Information

All expression plasmids of *Drosophila* were generated from the **Hy_ pMT EGFP SV40 pA Sense** plasmid (https://www.addgene.org/69911/), which is a modified form of the pMK33/pMtHy plasmid. Briefly, expression of the EGFP ORF (marked in green) that terminates in the SV40 polyadenylation signal (marked in pink) is driven by the metallothionein promoter (marked in blue). The copia transposon LTR promoter drives HygroR (marked in red) that terminates with an SV40 late poly(A) signal (marked in gray). An Amp selectable marker is also present. The full plasmid sequence is as follows:

GATCAATTCGTTGCAGGACAGGATGTGGTGCCCGATGTGACTAGCTCTTTGCTGCAGGCCGTCCTATCCTCTGGTTCCGAT
AAGAGACCCAGAACTCCGGCCCCCCACCGCCCACCGCCACCCCCATACATATGTGGTACGCAAGTAAGAGTGCCTGCGCAT
GCCCCATGTGCCCCACCAAGAGCTTTGCATCCCATACAAGTCCCCAAAGTGGAGAACCGAACCAATTCTTCGCGGGCAGAA
CAAAAGCTTCTGCACACGTCTCCACTCGAATTTGGAGCCGGCCGGCGTGTGCAAAAGAGGTGAATCGAACGAAAGACCCGT
GTGTAAAGCCGCGTTTCCAAAATGTATAAAACCGAGAGCATCTGGCCAATGTGCATCAGTTGTGGTCAGCAGCAAAATCAA
GTGAATCATCTCAGTGCAACTAAAGGGGGGATCTCGAGGTCGACGGTATCGATAAGCTTGATATCACCATGGTGAGCAAGG
GCGAGGAGCTGTTCACCGGGGTGGTGCCCATCCTGGTCGAGCTGGACGGCGACGTAAACGGCCACAAGTTCAGCGTGTCCG
GCGAGGGCGAGGGCGATGCCACCTACGGCAAGCTGACCCTGAAGTTCATCTGCACCACCGGCAAGCTGCCCGTGCCCTGGC
CCACCCTCGTGACCACCCTGACCTACGGCGTGCAGTGCTTCAGCCGCTACCCCGACCACATGAAGCAGCACGACTTCTTCA
AGTCCGCCATGCCCGAAGGCTACGTCCAGGAGCGCACCATCTTCTTCAAGGACGACGGCAACTACAAGACCCGCGCCGAGG
TGAAGTTCGAGGGCGACACCCTGGTGAACCGCATCGAGCTGAAGGGCATCGACTTCAAGGAGGACGGCAACATCCTGGGGC
ACAAGCTGGAGTACAACTACAACAGCCACAACGTCTATATCATGGCCGACAAGCAGAAGAACGGCATCAAGGTGAACTTCA
AGATCCGCCACAACATCGAGGACGGCAGCGTGCAGCTCGCCGACCACTACCAGCAGAACACCCCCATCGGCGACGGCCCCG
TGCTGCTGCCCGACAACCACTACCTGAGCACCCAGTCCGCCCTGAGCAAAGACCCCAACGAGAAGCGCGATCACATGGTCC
TGCTGGAGTTCGTGACCGCCGCCGGGATCACTCTCGGCATGGACGAGCTGTACAAGTAAGCGGCCGCAACTTGTTTATTGC
AGCTTATAATGGTTACAAATAAAGCAATAGCATCACAAATTTCACAAATAAAGCATTTTTTTCACTGCATTCTAGTTGTGG
TTTGTCCAAACTCATCAATGTATCTTAACTAGTGGATCCACTAGGGGCCGCCGACGCGAGGCTGGATGGCCTTCCCCATTA
TGATTCTTCTCGCTTCCGGCGGCATCGGGATGCCCGCGTTGCAGGCCATGCTGTCCAGGCAGGTAGATGACGACCATCAGG
GACAGCTTCAAGGATCGCTCGCGGCTCTTACCAGCCTAACTTCGATCATTGGACCGCTGATCGTCACGGCGATTTATGCCG
CCTCGGCGAGCACATGGAACGGGTTGGCATGGATTGTAGGCGCCGCCCTATACCTTGTCTGCCTCCCCGCGTTGCGTCGCG
GTGCATGGAGCCGGGCCACCTCGACCTGAATGGAAGCCGGCGGCACCTCGCTAACGGATTCACCACTCCAAGAATTGGAGC
CAATCAATTCTTGCGGAGAACTGTGAATGCGCAAACCAACCCTTGGCAGAACATATCCATCGCGTCCGCCATCTCCAGCAG
CCGCACGCGGCGCATCTCGGGCAGCGTTGGGTCCTGGCCACGGGTGCGCATGATCGTGCTCCTGTCGTTGAGGACCCGGCT
AGGCTGGCGGGGTTGCCTTACTGGTTAGCAGAATGAATCACCGATACGCGAGCGAACGTGAAGCGACTGCTGCTGCAAAAC
GTCTGCGACCTGAGCAACAACATGAATGGTCTTCGGTTTCCGTGTTTCGTAAAGTCTGGAAACGCGGAAGTCAGCGCTCTT

CCGCTTCCTCGCTCACTGACTCGCTGCGCTCGGTCGTTCGGCTGCGGCGAGCGGTATCAGCTCACTCAAAGGCGGTAATAC

GGTTATCCACAGAATCAGGGGATAACGCAGGAAAGAACATGTGAGCAAAAGGCCAGCAAAAGGCCAGGAACCGTAAAAAGG

CCGCGTTGCTGGCGTTTTTCCATAGGCTCCGCCCCCCTGACGAGCATCACAAAAATCGACGCTCAAGTCAGAGGTGGCGAA

ACCCGACAGGACTATAAAGATACCAGGCGTTTCCCCCTGGAAGCTCCCTCGTGCGCTCTCCTGTTCCGACCCTGCCGCTTA

CCGGATACCTGTCCGCCTTTCTCCCTTCGGGAAGCGTGGCGCTTTCTCATAGCTCACGCTGTAGGTATCTCAGTTCGGTGT

AGGTCGTTCGCTCCAAGCTGGGCTGTGTGCACGAACCCCCCGTTCAGCCCGACCGCTGCGCCTTATCCGGTAACTATCGTC

TTGAGTCCAACCCGGTAAGACACGACTTATCGCCACTGGCAGCAGCCACTGGTAACAGGATTAGCAGAGCGAGGTATGTAG

GCGGTGCTACAGAGTTCTTGAAGTGGTGGCCTAACTACGGCTACACTAGAAGGACAGTATTTGGTATCTGCGCTCTGCTGA

AGCCAGTTACCTTCGGAAAAAGAGTTGGTAGCTCTTGATCCGGCAAACAAACCACCGCTGGTAGCGGTGGTTTTTTTGTTT

GCAAGCAGCAGATTACGCGCAGAAAAAAAGGATCTCAAGAAGATCCTTTGATCTTTTCTACGGGGTCTGACGCTCAGTGGA

ACGAAAACTCACGTTAAGGGATTTTGGTCATGAGATTATCAAAAAGGATCTTCACCTAGATCCTTTTAAATTAAAAATGAA

GTTTTAAATCAATCTAAAGTATATATGAGTAAACTTGGTCTGACAGTTACCAATGCTTAATCAGTGAGGCACCTATCTCAG

CGATCTGTCTATTTCGTTCATCCATAGTTGCCTGACTCCCCGTCGTGTAGATAACTACGATACGGGAGGGCTTACCATCTG

GCCCCAGTGCTGCAATGATACCGCGAGACCCACGCTCACCGGCTCCAGATTTATCAGCAATAAACCAGCCAGCCGGAAGGG

CCGAGCGCAGAAGTGGTCCTGCAACTTTATCCGCCTCCATCCAGTCTATTAATTGTTGCCGGGAAGCTAGAGTAAGTAGTT

CGCCAGTTAATAGTTTGCGCAACGTTGTTGCCATTGCTGCAGGCATCGTGGTGTCACGCTCGTCGTTTGGTATGGCTTCAT

TCAGCTCCGGTTCCCAACGATCAAGGCGAGTTACATGATCCCCCATGTTGTGCAAAAAAGCGGTTAGCTCCTTCGGTCCTC

CGATCGTTGTCAGAAGTAAGTTGGCCGCAGTGTTATCACTCATGGTTATGGCAGCACTGCATAATTCTCTTACTGTCATGC

CATCCGTAAGATGCTTTTCTGTGACTGGTGAGTACTCAACCAAGTCATTCTGAGAATAGTGTATGCGGCGACCGAGTTGCT

CTTGCCCGGCGTCAACACGGGATAATACCGCGCCACATAGCAGAACTTTAAAAGTGCTCATCATTGGAAAACGTTCTTCGG

GGCGAAAACTCTCAAGGATCTTACCGCTGTTGAGATCCAGTTCGATGTAACCCACTCGTGCACCCAACTGATCTTCAGCAT

CTTTTACTTTCACCAGCGTTTCTGGGTGAGCAAAAACAGGAAGGCAAAATGCCGCAAAAAAGGGAATAAGGGCGACACGGA

AATGTTGAATACTCATACTCTTCCTTTTTCAATATTATTGAAGCATTTATCAGGGTTATTGTCTCATGAGCGGATACATAT

TTGAATGTATTTAGAAAAATAAACAAATAGGGGTTCCGCGCACATTTCCCCGAAAAGTGCCACCTGACGTCTAAGAAACCA

TTATTATCATGACATTAACCTATAAAAATAGGCGTATCACGAGGCCCTTTCGTCTTCAAGAATTCACATTTGTACGAATTT

TTTTTTTATCAAAAGTTCGAGTTTTTCACCAATTTCCTCATCAACCGAGCAAGGCAAACGGCTTTGAATAATATGGTGTTA

TATATACATATATCAAATCGCTGCTGACTGCGTGATTGATGGCCCCAAGATTACATATTATCGAATCAGGATTCAGAAGGA

GATCAATGTCAAATGCGGACAGGAACATGAAAGACGCCTGTTATGCGCAATTAAAAATTTGGGTTTAATTGCTGTGGAAAC

TGTTGTTGGCGGCATCTTAAGTTCCTGTTTAACAACATCAACTACTTATGTACGTAGAAGCGTTTAAGCCATTTGCATACA

GATGAGAACTGGCTTTTGTGCTAATCAGTCAAGATGACTCCGATGATGATGACTCATTACCTGACCAGTTTTCGCTGCTTT

CTTTTCAACAACTACTTGTATATGTATTGTATCCAATAGCAATACATTGAATTTCCATGGTCTAGTCACGTATTATCATTT

AATTGACACCAAGTCGTGTTATTGTTGAGCTATCGAGTTCAGCTCAAACATTTCTTATTCCCATGAATAAGCCGGCAAAAA

TATGCAATCTATGAAAGTTAATATAAGCAAACCTTACTTTGACTCAATACCAATGCACTTTGTGTCGATAGGTTCACGCAA

TTGAGGCGATTATTCCGATAACCCAAGCGATTGACTGTTCCCGTTTCGATTCCAATTGAAATTTGGAAATGTACAATAGTT

TTGCTATATGCTGTCAAGTACGCTCTTATCTTCTCTGGGTTTTCTTCAGAGTTTCGAAACGCTTCTTCTTTTTTTTGTTTT
TTTTTTTTTGGAATCTCGTATTTTGGAAGGGGCTCCCCTCTGGAATTTGTTACACTGTCGTTATCATTGCGAACAAGCGGC
CCGAAGCTATCAGCGACTTTAACATTTACAATGCACTTTTTTACGACCAATTAAATGTACATTTTCCTTTCTTCGCCCGTT
GATAAGCGAACGCGATGTGGCGCAGGCAATGTGTTGCTCTTGCGACACAAACGCAATCAAAATGGATTCAATTTCGCTTTT
TCCCAGTGAAACGAAGAACGAACCGACCATCATGATATGCTCCTCTGCATGTTGCGTATTGAATCAATGACAATTTCAATT
AAGCCGCCCGTTCGTCATGCGTTTTCGTGCGCTTCGAAATGCTGATAACGCTGCTGTCCTCCAACTGCTTTGCATGTGGAC
ACAATTCCATTTATTTAATTCTTTTATTTGGATCGGTTAAATTAAAAAGCGCCTTGTTACGCATTTAACGTTGTTTCCGGT
GCGTGGTGGTTTCATGCTTCTGGGAACGGCAAATGGGTTTAGGATTGGGAACCCCTCATCATCTGTTGGAATATACTATTC
AACCTACAAAAGTAACGTTAAACAACACTACTTTATATTTGATATGAATGGCCACACCTTTTATGCCATAAAACATATTGT
AAGAGAATACCACTCTTTTTATTCCTTCTTTCCTTCTTGTACGTTTTTTGCTGTAAGTAGGTCGTGGTGCTGGTGTTGCAG
TTGAAATAACTTAAAATATAAATCATAAAACTCAAACATAAACTTGACTATTTATTTATTTATTAAGAAAGGAAATATAAA
TTATAAATTACAACAGGTTATGGACCTGCAGCCAAGCTTGGCGCTCGTCCGGGGGCAATGAGATATGAAAAAGCCTGAACT
CACCGCGACGTCTGTCGAGAAGTTTCTGATCGAAAAGTTCGACAGCGTCTCCGACCTGATGCAGCTCTCGGAGGGCGAAGA
ATCTCGTGCTTTCAGCTTCGATGTAGGAGGGCGTGGATATGTCCTGCGGGTAAATAGCTGCGCCGATGGTTTCTACAAAGA
TCGTTATGTTTATCGGCACTTTGCATCGGCCGCGCTCCCGATTCCGGAAGTGCTTGACATTGGGGAATTCAGCGAGAGCCT
GACCTATTGCATCTCCCGCCGTGCACAGGGTGTCACGTTGCAAGACCTGCCTGAAACCGAACTGCCCGCTGTTCTGCAGCC
GGTCGCGGAGGCCATGGATGCGATCGCTGCGGCCGATCTTAGCCAGACGAGCGGGTTCGGCCCATTCGGACCGCAAGGAAT
CGGTCAATACACTACATGGCGTGATTTCATATGCGCGATTGCTGATCCCCATGTGTATCACTGGCAAACTGTGATGGACGA
CACCGTCAGTGCGTCCGTCGCGCAGGCTCTCGATGAGCTGATGCTTTGGGCCGAGGACTGCCCCGAAGTCCGGCACCTCGT
GCACGCGGATTTCGGCTCCAACAATGTCCTGACGGACAATGGCCGCATAACAGCGGTCATTGACTGGAGCGAGGCGATGTT
CGGGGATTCCCAATACGAGGTCGCCAACATCTTCTTCTGGAGGCCGTGGTTGGCTTGTATGGAGCAGCAGACGCGCTACTT
CGAGCGGAGGCATCCGGAGCTTGCAGGATCGCCGCGGCTCCGGGCGTATATGCTCCGCATTGGTCTTGACCAACTCTATCA
GAGCTTGGTTGACGGCAATTTCGATGATGCAGCTTGGGCGCAGGGTCGATGCGACGCAATCGTCCGATCCGGAGCCGGGAC
TGTCGGGCGTACACAAATCGCCCGCAGAAGCGCGGCCGTCTGGACCGATGGCTGTGTAGAAGTACTCGCCGATAGTGGAAA
CCGACGCCCCAGCACTCGTCCGAGGGCAAAGGAATAGAGTAGATGCCGACCGAACAAGAGCTGATTTCGAGAACGCCTCAG
CCAGCAACTCGCGCGAGCCTAGCAAGGCAAATGCGAGAGAACGGCCTTACGCTTGGTGGCACAGTTCTCGTCCACAGTTCG
CTAAGCTCGCTCGGCTGGGTCGCGGGAGGGCCGGTCGCAGTGATTCAGGCCCTTCTGGATTGTGTTGGTCCCCAGGGCACG
ATTGTCATGCCCACGCACTCGGGTGATCTGACTGATCCCGCAGATTGGAGATCGCCGCCCGTGCCTGCCGATTGGGTGCAG
ATCTTTGTGAAGGAACCTTACTTCTGTGGTGTGACATAATTGGACAAACTACCTACAGAGATTTAAAGCTCTAAGGTAAAT
ATAAAATTTTTAAGTGTATAATGTGTTAAACTACTGATTCTAATTGTTTGTGTATTTTAGATTCCAACCTATGGAACTGAT
GAATGGGAGCAGTGGTGGAATGCCTTTAATGAGGAAAACCTGTTTTGCTCAGAAGAAATGCCATCTAGTGATGATGAGGCT
ACTGCTGACTCTCAACATTCTACTCCTCCAAAAAAGAAGAGAAAGGTAGAAGACCCCAAGGACTTTCCTTCAGAATTGCTA
AGTTTTTTGAGTCATGCTGTGTTTAGTAATAGAACTCTTGCTTGCTTTGCTATTTACACCACAAAGGAAAAAGCTGCACTG
CTATACAAGAAAATTATGGAAAAATATTCTGTAACCTTTATAAGTAGGCATAACAGTTATAATCATAACATACTGTTTTTT

CTTACTCCACACAGGCATAGAGTGTCTGCTATTAATAACTATGCTCAAAAATTGTGTACCTTTAGCTTTTTAATTTGTAAA

GGGGTTAATAAGGAATATTTGATGTATAGTGCCTTGACTAGAGATCATAATCAGCCATACCACATTTGTAGAGGTTTTACT

TGCTTTAAAAAACCTCCCACACCTCCCCCTGAACCTGAAACATAAAATGAATGCAATTGTTGTTGTTAACTTGTTTATTGC

AGCTTATAATGGTTACAAATAAAGCAATAGCATCACAAATTTCACAAATAAAGCATTTTTTTCACTGCATTCTAGTTGTGG

TTTGTCCAAACTCATCAATGTATCTTATCATGTCTG

The following three plasmids were generated by inserting the indicated sequences into the XhoI and NotI sites of **Hy_pMT EGFP SV40 pA Sense**, thereby replacing the EGFP ORF with the indicated sequences.

**Hy_pMT laccase2 Exons 1-3**

Previously described in Liang et al. 2017; https://www.addgene.org/91799/

**(Exon 1 in green, Exon 2 in yellow, Exon 3 in blue)**

CTTCAGTTTGGTGCTTAAACGCGTTGCTCGTACTTCATTAAGCCGTAATCAATTCAGGTGGTGTTGCTATTGCTTAGTACA
CATACTTTAAAAATATATAGTCTTATGTTGTGAACATCCACCCAAAGTATTGTTAAATAGCTTAAAGGAATTAATAAAAAT
ACAAGAAGAATAGTGAAAAATAAAATTATACGCCATTCATACAACTATGAAAGCCATGGATCTTAACGGATTTTACCAAAC
TTTTATTTCGTTCCTGATATGGGGTGCATTTTCCAATTTTCCTGAAGTATTTGCAGTACGTGTTGCCAACTCACAGCAAAA
TCAACCTAAGAAAGGTAAGGAAATCCCATCTTGGTAATTTTTTTCACCTAAGAAGTCTTGAAATATCATTTTGTGATTTGT
CGACAGTACATATTCCTTTCATTAAAATGATTATTTAAGTGCAATACGTATATACTCGTATACATCTAAACATGACTATAT
TTCCAAGGGGTGTTCCAAAGATTTGGGGGATCTTTTGGGCTCGAGCATTGAGAAATGACTGAGTTCCGGTGCTCTCAAGTC
ATTGATCTTTGTCGACTTTTATTTGGTCTCTGTAATAACGACTTCAAAAACATTAAATTCTGTTGCGAAGCCAGTAAGCTA
CAAAAAGAAAAAACAAGAGAGAATGCTATAGTCGTATAGTATAGTTTCCCGACTATCTGATACCCATTACTTATCTAGGGG
GAATGCGAACCCAAAATTTTATCAGTTTTCTCGGATATCGATAGATATTGGGGAATAAATTTAAATAAATAAATTTTGGGC
GGGTTTAGGGCGTGGCAAAAAGTTTTTTGGCAAATCGCTAGAAATTTACAAGACTTATAAAATTATGAAAAAATACAACAA
AATTTTAAACACGTGGGCGTGACAGTTTTGGACGGTTTTAGGGCGTTAGAGTAGGCGAGGACAGGGTTACATCGACTAGGC
TTTGATCCTGATCAAGAATATATATACTTTATACCGCTTCCTTCTACATGTTACCTATTTTTCAACGAATCTAGTATACCT
TTTTACTGTACGATTTATGGGTATAATAATAAGCTAAATCGAGACTAAGTTTTATTGTTATATATATTTTTTTTATTTTAT
GCAGAAAGGTACCAAAATCAACCAGATCTTACACCAATGGGGTCCTTCTGGCAGCAACATGTGGCCACTCCTAGTAGTTCA
ACTCCATTCGATTCACCATTTTTTTCGGCAACACACGGCCTTGTGCAGACTCACCCGTCTATAGGTGGAAGCCACTCTGGG
CCCGCTCCATTAAATGGCAATAGCCTTGGGTCTGGAGCTCCATCCTCAATCCTAGCTGGTACTAAGCCAGCCCTAACCGCT
GGAAGCGGTTACCTAAACTCACGCGGGAGTCTACCGACCTCTGCACGTTATCCAGCTAATAAGATCACAGGAACCGTCGTG
GAACCCAACCCAAAATCTCCATTCCGCCACTTAGATTTCTCTACTAGTGCAACTGCCGAGCTGCGGCGCAATCCTGCGCTA
TCTGCTCCTGATGAATGTGCACGCGCTTGTCGCGAAGGCGAGCCTCCGAGAATTTGCTACTATCATTTTACGTTGGAATAC
TACACAGTATTGGGAGCCCCGGGGAGCTCAGCGTAAGTATTCAAAATTCCAAAATTTTTTACTAGAAATATTCGATTTTTT
AATAGGCAGTTTCTATACTATTGTATACTATTGTAGATTCGTTGAAAAGTATGTAACAGGAAGAATAAAGCATTTCCGACC
ATGTAAAGTATATATATATTCTTAATAAGGATCAATAGCCGAGTCGATCTCGCCATGTCCGTCTGTCTTATTATTTTATTACC
GCCGAGACATCAGGAACTATAAAAGCTAGAAGGATGAGTTTTAGCATACAGATTCTAGAGACAAGGACGCAGAGCAAGTTT
GTTGATCCATGCTGCCACGCTTTAACTTTCTCAAATTGCCCAAAACTGCCATGCCCACATTTTTGAACTATTTTCGAAATT
TTTTCATAATTGTATTACTCGTGTAAATTTCCATCAATTTGCCAAAAAACTTTTTGTCACGCGTTAACGCCCTAAAGCCGC
CAATTTGGTCACGCCCACACTATTGAACAATTATCAAATTTTTTCTCATTTTATTCCCCAATATCTATCGATATCCCCGAT
TATGAAATTATTAAATTTCGCGTTCGCATTCACACTAGCTGAGTAACGAGTATCTGATAGTTGGGGAAATCGACTTATTTT

TTATATACAATGAAAATGAATTTAATCATATGAATATCGATTATAGCTTTTTATTTAATATGAATATTTATTTGGGCTTAA

GGTGTAACCTCCTCGACATAAGACTCACATGGCGCAGGCACATTGAAGACAAAAATACTCATTGTCGGGTCTCGCACCCTC

CAGCAGCACCTAAAATTATGTCTTCAATTATTGCCAACATTGGAGACACAATTAGTCTGTGGCACCTCAGGCGGCCGCCAG

AAACCCTGGTCACGCCCCCTTTCACCTACACTCCCAAGATAGACACACAAACACTGTTAAACAATTTTTAAATATTTTTTC

ATTTTATTCACCAATATTGGCTACACTTAATATGTCTTTCCTTACAATTGAGCAAGTTTAAAATAAATAAATCAAATTAAT

ATATTTTTATCGGATTGACCTAATTATAATTATAATTATTTACACACAGTGCGTGTCAAGTATGTACTCCAAACGCAACGA

ATACGGTTTGGAGCCATTGTCAGTGTGTCTTAGCTGATGGAGTTGAACGTGGAATTCTAACTGCGAATCGTATGATACCCG

GGCCAAGCATTCAAGTGTGTGAAAACGACAAAGTCGTTATTGATGTGGAGAACCATATGGAGGGAATGGAAGTAACTATAC

ACTGGCACGGTATATGGCAACGTGGATCGCAGTATTATGATGGGGTGCCCTTCGTTACTCAGTGCCCCATTCAGCAGGGTA

ATACCTTCCGTTATCAGTGGACAGGAAACGCTGGGACACATTTTTGGCATGCTCATACCGGTCTACAGAAACTAGATGGAC

TCTACGGCAGCGTCGTGGTTCGACAGCCACCATCAAGGGACCCTAACTCCCATTTATATGATTTTGATTTAACAACACATA

TAATGCTTATCAGTGATTGGTTGCATGAAGATGCAGCAGAGAGATATCCTGGACGCTTGGCTGTTAACACAGGCCAAGATC

CGGAGTCTATGTTGATTAACGGAAAAGGCCAGTTTCGTGACCCGAACACTGGATTTATGACTAATACTCCGTTGGAAATAT

TCACAATTACGCCAGGACGTCGTTACCGCTTTCGAATGATAAATGCATTTGCTTCAGTCTGTCCAGCACAGGTAACAATTG

AGGGTCACGGCATGACGGTTATAGCAACCGATGGTGAGCCTGTGCATCCTGTTGATGTAAATACAAT

**Hy_pMT dati Exons 1-3**

Previously described in Huang et al. 2018; https://www.addgene.org/110113/

**(Exon 1 in green, Exon 2 in yellow, Exon 3 in blue)**

AGTTTTTCTATGACTCGCGGGCCGCCAGGTTATCTGTAGTTGTTTGCTGGGAGCGACTTCCAAGTCTCAGCCGTAATCGTA

ACCGTAATCGAGGCCGTGCATATAGACAGTCACTGTATTCTTCCAACAAAAAAAACCTCGTAAAGAGGTAAAAGCAACCGA

AATACGCACCAAACCACCAATTATACAAATGGCAGCAAGATAACATTTCGCTCCTTTTCGAACACTTGCATACGCGACACA

TTGCTTATTATTGGTTTTATTCACAAATTTTGGTTTTTTTATGCTTTGAACTTTAGTTTCTTTTCTTTAAAAAGTGATTAA

AACAGTTTGGTGACTATGTTCCATCCCCAATAATAACAAACAATTTATAAGTGTCCATGTGCGACGCTAGGATATTCAAGA

ATAAGTTTCCTAATTGACCTGTGTGTTTTATTTATTTCGACGCAACTACGTCTACGAGGAAATATATTAATTTTTCAAAAC

CGATGCCAACATATATGTGGCTTGGTAACTTAAGTCGTCCAGAAACTGGCTTATACAAGAAACCGTATTCTTATATATTAA

GGCTCTACCTTCGATTGCTTTTAAATCAAATTGTCTATAAATTGAATCAGAAACGTTAAATTCTGGGACAAAAGCAATTTT

GGTAGACTGACTTTTTTAAACAGAATGACCGATATATTAGCTATGGTGTTTATATATATATTTTAAATATATATCTTAAGA

GACGTATGCGTATCTCACGTTTTTAATATATACAAAATACGGAAGTAATTTAATAATTCTTAATTTGATTTATTAATCGAA

AGAATACGTGTAAAAAAAAGTTAGATCATTATACACTTTTTTACTTCATGGTTTTACTTCCATGGTTTGGCTTAAAAAAAA

AGCACTCCAATTTTATATCCATTATTTTAGAGGAAATCATCCTGAGGCACTAGAGAAATGTTGTTATTTCTTAATGATTTA

ATTGATAGGTATACTGCTTCCTCGAGCCTCTGCCATGACTATCGTAGAGTAAAAAGGGTTAAGCATTTCCGACCATATAAA

GTATAAATACTCTTGATCAGAATCAATAGCCGAGTCGAACTGGCCATGTCCGCCTCAACCTATGAGGATTGAAACAAGCAT

TCATTCTGAATACATAGACGCAGCGCAAGTTTGTTGACTCATATTGTCACACTCACTCTAACGCCCACAAAGCGCCTTAAC

CTGCCACGGACACCTTTTTGAAATTCGAAATATTTCGTTATCTTTTCATATTTTTATTAGTTTTGTAAATTTATATCGAGT

TGCAAAAAAAACTATTCCACGCCAACTATAACTCCCACAAACCGCCCAAAAATGCCACACCCACTCTTTTAAGAAATGATT

CAATTTTGTTGGATTAAATAAGTCGTTTTCCTTATTTCAATCTATATGCCGAAAACATATTGACCACGCCCACTCTGACCC

CTAAAACCGCCCAATTTTAAAAATTCAAAAAACCCTTTTACGCTTAACAACTTTTTTTGTTTTACAGGCAATAAAATGATA

TGAAATTACGATGCTTTAACCAGGCCCAAACTTCGCACAGTTGGCACCCCAGATCCGTGTTCAGGGTCGTAAGCAGTTGTC

CTCCCGATCGAAAAAAACGGCCAAGCCCAGCGAGCTGGCAAATCTGTATTGGCCATCTCCTAACAAATTATCTTGGAATGT

TTAAAATGGACTCCGCAGACTTCTGGCAGCAAGCGCGTGCTCCGTTTGGTCTGCAAACTGCGCTTCACCAATACTCTTCCC

CCCCGAATCAACAGCCAATATCCCACCACGCCCTGCACCACTCCGTTCCGCAGGAGAACGAGCAGTTGGCCGGAGTTCAAC

CAGAAGGTCCAGCAGCAGGATCAGGCGTAGTGGGCAACAACAGTTCCATGGCAATTTCCGGCACCAATTCAACTGTGGGCA

GCAAGGCGGAGTCCAGTCACATTCCGCAGCAGCACTCAGAGCAGCAGTCATATGGAAGCGACTCCTTCCGAGGAACGCAGT

CGCCCCAGCTGAGTAGCCACCACCTGTTGTTTAACGCCGCTGCAGCCGCTGCTGCGGCGGTCCACCTCAAGTCCACGGCGA

TGCAGAATAACCTCTCGCCAATAGGCGATCAGGTGCAAAATAACCTTCGCAACTACGGACAAGGATCCTTAAACGCCTTAT

GTGGGATCAAGCCCAAGCAGGAGATGGATGCGAAGACGCCCCTACAGCCGTTGGACGAGTGTCCCCACCCTCTAGTTCAGG

CGCAGGCCCAGTCCCAGTACGGCGGAGACTACTACGACGTGGCCGACCCACAGGCTAGAGAAATCTCGGAAGGTCGAGCAC

TGATAATCGGACTCGGAGCACCCAGTACGGTGTCCACCGACGATGCACAGTCCAGTGCGCCGTCTCATCAGCTGGCAGGGA

CTGGCCGAGCATTGCAGACTCATCAGTGCAAGCCAATGTCCCCTGGAACAGTTGGTAGCTCGAATTTGGGCGCAGGACGAA

GATCCGCACCCACAACAATAAGCAAGACCTTTTCGCAGGGTCAGCAGTCCCCTCAGCACTCTACAACGCCATCCGGAGGCA

GCACGACGCCCGACATCAAATACAATAACGACAAAATGGCCAATGAAATTCAGGTATTGTTTTGAAGATTTTTCTGCTTAT

CTATATACTTTAAAGGGAACCTGGTAATCGAATATTCGATGCAGTCCAAACAAGAGTAGTAAAAAATCAACAGAGAACGCT

ATTCGAGTTTTTAGACTAGACTATATGTACACCCGATATTTAGCTGTTACGAGCGCAACAATGAACTTTCAAAATTGTGTG

ACTGTTTTGGACGTTTTGCAGGTGTAAGTAGGGGCGTGGCCAAAGTGTTTTTGGTATGTCAATAAAAATTGGCAAGACAAT

GAAAACGAAGATTAATCAAAACATTTTTTGTGGGGGCGTGGCAAACAGTTTTTTGGCAAATCGATTTTATTTTATCGATGA

AATAATATGAATACATTTTGCAAGAGCGTGGGAGTGGCAGTTCTGGACGCTTTGTGGGCGTTAGAGGATGCGTAGCAACAA

ACTTGCGCTGCGATCTATGCTTAATCTCAACTTTCTAGCTTTTAGTTAGATAGTTTCTGAAATCTCGACGTTCATACAGAC

GGATATGGCCAGTTCGACTCGTCTTTTGATGCTAATCAAGAATATATGTAGTTTATATGGTCGGAAACGCTTCCTTCTACC

TGTTACATACCTTTAAACAAATCTAGTATACACTTTCCCTCTACGAGTAATGGGTATGAAAACAAAAATCAACACGCATCA

ACGCATTTTGGAAAGTTGAAGGCGTGATAGTGCGGCCGCGGAGCGGACGTATATGTGAGTTTAAATATATATGTGTGAATG

GCGATAGAAAGTGGATATTACAAAAAATCTGAAAGTGGACAAATTTTGTGGGCGTAACAGCATTTTGCATCTTGTCGGCGT

CAGAGTGGGTGTGGGCAATATATAATATTGGCAACACAAATAATGAAAAAGAAAGGCGTGAAAATTTCGGGCGTTACTAGA

ATCTTCCCGCTTATCGTAACTTTCCAGCAATAAGTTTCCGACTCCAGGTTCCAGATTCTGATCCAGATCCAGATTTCAAAT

ATACATATATAATTTGTAGGGTGTGAAACACTTCTTCAGGTAGCAAGACGAATTTAGTATAAAAAATATAACTATTTCTAC

ATATATACATTAAAATTTAATTAAATGTGGGCTCCACGCTGTAGAAATCAATTGACTATATCAAACTATATATAACTATAA

CTATAACTATACATAACAAAATGGCAAAAACAAAGTTTGGAGAGAAAAATACCATTTTTAGCAAGGGAACAGGTATCATTC

TTGCAGGCGTTATTTCAATTTTAAAATTTTTCTAAGCTACACCTAATAGTGGTAAAAAGGCAACCTCGTTATACATATATG

GCATAGCTTATCACTCCTTGGGTGAATAGATGTGTGAGCCTGAGCACCGCTATTATTACCCCCTCATGAAAGAAAATGATG

TATTTTTTGTTTGCTTTTCAGCTTCAGCTATCCCGTTCGAGCAGCGCAGCGGCGATCAGTGAACGCACCCTCGAAGAATGT

TGGTCAACCCTACAACGA

**Hy_pMT Flag MCS SV40** **Note: This plasmid was used for subsequent cloning**

Previously described in Huang et al. 2018.

ACCATGGACTACAAAGACCATGACGGTGATTATAAAGATCATGACATCGATTACAAGGATGACGATGACAAGACCGGTGGT

ACCGCTAGCTTAATTAAGAGCTCCCCGGG

The following plasmid was generated by inserting the indicated sequences into the XhoI and AgeI sites of **Hy_pMT EGFP SV40 pA Sense**, thereby replacing the EGFP ORF with the indicated sequences.

**Hy_pMT V5 MCS SV40** **Note: This plasmid was used for subsequent cloning**

ACCATGGGTAAGCCTATCCCTAACCCTCTCCTCGGTCTCGATTCTACG

The following seven plasmids were generated by inserting the indicated sequences into the XmaI and NotI sites of **Hy_pMT Flag MCS SV40**.

**Hy_pMT Flag_GW182_WT MCS SV40**

ATGCGTGAAGCCCTTTTTTCCCAAGATGGCTGGGGCTGTCAGCATGTTAACCAGGATACTAATTGGGAAGTTCCCAGTTCG

CCAGAACCAGCCAATAAGGATGCACCCGGTCCACCAATGTGGAAGCCAAGCATTAACAATGGTACTGATCTTTGGGAGTCC

AATTTGAGAAACGGAGGTCAGCCGGCCGCACAGCAAGTTCCAAAGCCGTCGTGGGGTCATACACCATCCTCTAACTTAGGT

GGAACATGGGGTGAGGACGACGATGGCGCCGATAGTAGTAGTGTGTGGACTGGAGGAGCTGTTAGCAACGCGGGATCCGGA

GCTGCAGTGGGAGTAAACCAAGCCGGAGTTAATGTCGGTCCAGGCGGTGTTGTTTCGTCTGGCGGACCTCAGTGGGGACAA

GGTGTCGTTGGCGTCGGACTTGGATCAACTGGAGGTAACGGGTCAAGCAATATAACTGGATCGTCTGGAGTCGCAACAGGT

AGTAGCGGAAACTCCAGCAACGCTGGTAACGGTTGGGGAGACCCTCGTGAAATACGCCCTTTGGGAGTTGGTGGCTCCATG

GATATTCGAAATGTTGAACATCGCGGCGGTAACGGTTCTGGAGCAACTTCGAGCGATCCACGAGACATTCGCATGATCGAT

CCGCGTGACCCTATTCGAGGAGATCCCCGTGGAATATCTGGTCGTCTTAATGGGACCTCTGAAATGTGGGGTCATCATCCA

CAAATGTCCCATAACCAGTTGCAAGGTATCAACAAAATGGTTGGTCAAAGTGTAGCAACTGCCAGCACCAGTGTCGGAACA

TCTGGCTCGGGCATCGGTCCTGGAGGTCCCGGTCCTAGTACAGTATCAGGCAATATCCCAACACAGTGGGGGCCTGCTCAA

CCGGTAAGCGTTGGTGTAAGTGGTCCCAAAGACATGTCAAAACAGATAAGTGGATGGGAGGAACCATCACCACCGCCTCAG

CGTCGCAGTATTCCTAACTACGATGATGGTACATCGTTGTGGGGTCAGCAAACTCGTGTTCCCGCTGCAAGCGGTCACTGG

AAAGACATGACTGATTCGATAGGTCGTAGTAGTCATCTCATGCGTGGCCAAAGCCAAACGGGAGGTATAGGAATAGCCGGC

GTTGGAAATAGCAATGTTCCAGTGGGAGCCAATCCAAGTAATCCTATAAGCAGTGTAGTTGGACCTCAAGCCCGGATTCCA

TCTGTGGGCGGCGTACAACACAAACCAGACGGCGGCGCTATGTGGGTGCATTCCGGCAATGTAGGTGGCAGAAATAATGTT

GCTGCTGTTACTACTTGGGGAGATGACACTCATAGCGTTAATGTCGGCGCTCCCAGCAGTGGCAGTGTATCCAGCAACAAT

TGGGTTGATGACAAGTCCAACTCAACCTTGGCACAAAACTCTTGGAGCGACCCGGCCCCTGTTGGAGTTAGTTGGGGCAAT

AAGCAAAGCAAACCGCCAAGCAATAGTGCTTCATCAGGTTGGAGCACTGCTGCGGGCGTGGTGGATGGGGTTGATCTAGGA

TCTGAGTGGAACACGCACGGGGGGATTATTGGAAAATCTCAGCAACAACAAAAACTAGCGGGACTTAACGTGGGAATGGTG

AACGTAATTAACGCGGAGATCATTAAGCAAAGCAAGCAATACAGGATCCTTGTCGAGAACGGCTTTAAAAAGGAAGATGTA

GAGCGGGCATTAGTGATTGCTAATATGAACATCGAAGAGGCAGCCGATATGCTCCGTGCCAACTCATCCCTATCAATGGAT

GGTTGGCGTCGACATGATGAGTCCCTTGGATCTTATGCCGACCACAATAGTTCAACAAGCAGCGGTGGATTTGCTGGTCGT

TACCCGGTCAACAGTGGACAACCTTCAATGTCCTTTCCTCATAATAACCTTATGAATAACATGGGAGGTACCGCTGTTACT

GGAGGTAACAACAATACAAACATGACAGCTTTACAGGTGCAAAAGTATTTAAATCAAGGGCAACATGGTGTCGCTGTTGGA

CCGCAAGCCGTTGGTAATTCTTCAGCAGTATCTGTCGGATTTGGTCAGAACACGTCTAACGCAGCAGTGGCAGGAGCAGCC

TCTGTAAATATAGCAGCAAATACAAACAACCAACCGTCTGGTCAGCAAATTCGCATGCTAGGCCAGCAAATTCAGTTGGCC

ATTCATAGTGGTTTCATATCTAGTCAGATATTGACTCAACCGCTAACTCAAACAACCCTTAACCTTTTAAACCAACTTCTT

AGCAATATTAAGCATCTCCAGGCTGCGCAGCAATCCCTTACCCGCGGGGGAAATGTCAATCCAATGGCAGTGAATGTGGCT

ATATCTAAATACAAGCAGCAAATCCAGAATTTACAGAACCAGATAAATGCACAACAGGCTGTGTATGTAAAACAGCAAAAT

ATGCAACCAACTTCACAACAACAACAGCCCCAACAACAGCAACTTCCTTCTGTTCATCTAAGTAACTCAGGCAACGACTAT

TTAAGAGGTCACGATGCAATAAATAATTTGCAAAGCAACTTTTCTGAGCTCAATATTAATAAGCCAAGTGGATATCAAGGA

GCGTCCAATCAACAATCCCGATTAAATCAGTGGAAGCTTCCAGTATTAGATAAGGAGATCAACTCTGACAGTACGGAATTT

TCTCGTGCCCCAGGTGCAACGAAACAAAATTTGACGGCCAACACAAGCAACATAAACTCTTTGGGTCTTCAAAACGATAGT

ACATGGTCAACTGGACGCAGTATTGGTGACGGTTGGCCTGATCCCTCATCTGATAACGAGAATAAAGACTGGTCTGTTGCT

CAGCCAACTTCAGCAGCAACTGCTTACACTGATCTGGTCCAAGAGTTTGAGCCAGGCAAGCCATGGAAGGGTTCACAGATC

AAAAGCATAGAAGATGATCCCAGCATTACACCAGGAAGCGTTGCTAGATCTCCATTGTCTATTAATTCGACGCCAAAAGAT

GCTGACATATTTGCCAATACCGGTAAAAATTCACCGACTGATTTACCGCCACTAAGTTTATCGTCGTCTACATGGAGTTTT

AATCCAAACCAAAATTATCCGAGTCACAGTTGGTCTGACAATAGTCAACAATGTACCGCCACTTCGGAGCTTTGGACAAGC

CCGCTAAATAAATCATCGTCTCGAGGTCCCCCGCCAGGATTGACTGCCAATTCAAATAAATCTGCAAATAGTAATGCGTCA

ACGCCAACAACTATTACCGGAGGTGCGAATGGATGGTTACAGCCTCGAAGTGGCGGTGTTCAAACCACAAACACTAATTGG

ACAGGTGGTAACACCACTTGGGGCTCCAGTTGGTTGCTTTTGAAAAATCTAACAGCACAGATTGATGGTCCTACTTTGCGT

ACACTGTGTATGCAGCATGGGCCCCTTGTCAGCTTTCACCCGTATTTGAACCAAGGAATTGCCTTATGTAAATATACTACT

CGTGAGGAGGCGAACAAGGCGCAAATGGCGTTAAACAACTGTGTCCTCGCCAACACCACAATATTTGCTGAATCTCCCAGC

GAGAACGAGGTGCAAAGCATTATGCAGCACTTACCACAAACTCCTTCCTCTACAAGCTCTAGTGGAACTAGTGGTGGCAAC

GTCGGAGGCGTCGGCACTTCAGCCAATAATGCAAACAGTGGTTCTGCAGCTTGTCTGTCCGGAAACAATAGCGGCAACGGA

AACGGCAGCGCGAGCGGCGCCGGCAGCGGCAACAATGGCAACAGTAGCTGCAACAACAGTGCCGCCGGGGGGGGCAGCAGC

AGCAACAACACGATTACCACTGTAGCAAATTCGAATCTTGTTGGTTCTAGTGGCTCTGTCTCAAATTCCTCTGGCGTTACT

GCTAACTCTAGTACTGTTTCTGTAGTTAGTTGTACAGCGAGTGGGAATTCCATAAATGGGGCAGGTACTGCAAACAGTTCT

GGTTCAAAGAGTAGTGCAAACAATTTAGCTAGCGGCCAGTCTAGCGCTTCTAACTTAACTAATAGCACCAATTCAACATGG

CGACAAACTAGCCAAAACCAAGCTCTTCAAAGTCAAAGCAGGCCATCAGGCAGAGAAGCTGACTTTGATTATATATCTCTC

GTTTATTCCATTGTTGATGATTAA

**Hy_pMT Flag_GW182_ΔABD MCS SV40**

**(GW182 is depleted of Ago-binding domain.)**

GTGAACGTAATTAACGCGGAGATCATTAAGCAAAGCAAGCAATACAGGATCCTTGTCGAGAACGGCTTTAAAAAGGAAGAT

GTAGAGCGGGCATTAGTGATTGCTAATATGAACATCGAAGAGGCAGCCGATATGCTCCGTGCCAACTCATCCCTATCAATG

GATGGTTGGCGTCGACATGATGAGTCCCTTGGATCTTATGCCGACCACAATAGTTCAACAAGCAGCGGTGGATTTGCTGGT

CGTTACCCGGTCAACAGTGGACAACCTTCAATGTCCTTTCCTCATAATAACCTTATGAATAACATGGGAGGTACCGCTGTT

ACTGGAGGTAACAACAATACAAACATGACAGCTTTACAGGTGCAAAAGTATTTAAATCAAGGGCAACATGGTGTCGCTGTT

GGACCGCAAGCCGTTGGTAATTCTTCAGCAGTATCTGTCGGATTTGGTCAGAACACGTCTAACGCAGCAGTGGCAGGAGCA

GCCTCTGTAAATATAGCAGCAAATACAAACAACCAACCGTCTGGTCAGCAAATTCGCATGCTAGGCCAGCAAATTCAGTTG

GCCATTCATAGTGGTTTCATATCTAGTCAGATATTGACTCAACCGCTAACTCAAACAACCCTTAACCTTTTAAACCAACTT

CTTAGCAATATTAAGCATCTCCAGGCTGCGCAGCAATCCCTTACCCGCGGGGGAAATGTCAATCCAATGGCAGTGAATGTG

GCTATATCTAAATACAAGCAGCAAATCCAGAATTTACAGAACCAGATAAATGCACAACAGGCTGTGTATGTAAAACAGCAA

AATATGCAACCAACTTCACAACAACAACAGCCCCAACAACAGCAACTTCCTTCTGTTCATCTAAGTAACTCAGGCAACGAC

TATTTAAGAGGTCACGATGCAATAAATAATTTGCAAAGCAACTTTTCTGAGCTCAATATTAATAAGCCAAGTGGATATCAA

GGAGCGTCCAATCAACAATCCCGATTAAATCAGTGGAAGCTTCCAGTATTAGATAAGGAGATCAACTCTGACAGTACGGAA

TTTTCTCGTGCCCCAGGTGCAACGAAACAAAATTTGACGGCCAACACAAGCAACATAAACTCTTTGGGTCTTCAAAACGAT

AGTACATGGTCAACTGGACGCAGTATTGGTGACGGTTGGCCTGATCCCTCATCTGATAACGAGAATAAAGACTGGTCTGTT

GCTCAGCCAACTTCAGCAGCAACTGCTTACACTGATCTGGTCCAAGAGTTTGAGCCAGGCAAGCCATGGAAGGGTTCACAG

ATCAAAAGCATAGAAGATGATCCCAGCATTACACCAGGAAGCGTTGCTAGATCTCCATTGTCTATTAATTCGACGCCAAAA

GATGCTGACATATTTGCCAATACCGGTAAAAATTCACCGACTGATTTACCGCCACTAAGTTTATCGTCGTCTACATGGAGT

TTTAATCCAAACCAAAATTATCCGAGTCACAGTTGGTCTGACAATAGTCAACAATGTACCGCCACTTCGGAGCTTTGGACA

AGCCCGCTAAATAAATCATCGTCTCGAGGTCCCCCGCCAGGATTGACTGCCAATTCAAATAAATCTGCAAATAGTAATGCG

TCAACGCCAACAACTATTACCGGAGGTGCGAATGGATGGTTACAGCCTCGAAGTGGCGGTGTTCAAACCACAAACACTAAT

TGGACAGGTGGTAACACCACTTGGGGCTCCAGTTGGTTGCTTTTGAAAAATCTAACAGCACAGATTGATGGTCCTACTTTG

CGTACACTGTGTATGCAGCATGGGCCCCTTGTCAGCTTTCACCCGTATTTGAACCAAGGAATTGCCTTATGTAAATATACT

ACTCGTGAGGAGGCGAACAAGGCGCAAATGGCGTTAAACAACTGTGTCCTCGCCAACACCACAATATTTGCTGAATCTCCC

AGCGAGAACGAGGTGCAAAGCATTATGCAGCACTTACCACAAACTCCTTCCTCTACAAGCTCTAGTGGAACTAGTGGTGGC

AACGTCGGAGGCGTCGGCACTTCAGCCAATAATGCAAACAGTGGTTCTGCAGCTTGTCTGTCCGGAAACAATAGCGGCAAC

GGAAACGGCAGCGCGAGCGGCGCCGGCAGCGGCAACAATGGCAACAGTAGCTGCAACAACAGTGCCGCCGGGGGGGGCAGC

AGCAGCAACAACACGATTACCACTGTAGCAAATTCGAATCTTGTTGGTTCTAGTGGCTCTGTCTCAAATTCCTCTGGCGTT

ACTGCTAACTCTAGTACTGTTTCTGTAGTTAGTTGTACAGCGAGTGGGAATTCCATAAATGGGGCAGGTACTGCAAACAGT

TCTGGTTCAAAGAGTAGTGCAAACAATTTAGCTAGCGGCCAGTCTAGCGCTTCTAACTTAACTAATAGCACCAATTCAACA

TGGCGACAAACTAGCCAAAACCAAGCTCTTCAAAGTCAAAGCAGGCCATCAGGCAGAGAAGCTGACTTTGATTATATATCT

CTCGTTTATTCCATTGTTGATGATTAA

**Hy_pMT Flag_GW182_ΔUAB MCS SV40**

**(GW182 is depleted of ubiquitin-associated domain.)**

ATGCGTGAAGCCCTTTTTTCCCAAGATGGCTGGGGCTGTCAGCATGTTAACCAGGATACTAATTGGGAAGTTCCCAGTTCG

CCAGAACCAGCCAATAAGGATGCACCCGGTCCACCAATGTGGAAGCCAAGCATTAACAATGGTACTGATCTTTGGGAGTCC

AATTTGAGAAACGGAGGTCAGCCGGCCGCACAGCAAGTTCCAAAGCCGTCGTGGGGTCATACACCATCCTCTAACTTAGGT

GGAACATGGGGTGAGGACGACGATGGCGCCGATAGTAGTAGTGTGTGGACTGGAGGAGCTGTTAGCAACGCGGGATCCGGA

GCTGCAGTGGGAGTAAACCAAGCCGGAGTTAATGTCGGTCCAGGCGGTGTTGTTTCGTCTGGCGGACCTCAGTGGGGACAA

GGTGTCGTTGGCGTCGGACTTGGATCAACTGGAGGTAACGGGTCAAGCAATATAACTGGATCGTCTGGAGTCGCAACAGGT

AGTAGCGGAAACTCCAGCAACGCTGGTAACGGTTGGGGAGACCCTCGTGAAATACGCCCTTTGGGAGTTGGTGGCTCCATG

GATATTCGAAATGTTGAACATCGCGGCGGTAACGGTTCTGGAGCAACTTCGAGCGATCCACGAGACATTCGCATGATCGAT

CCGCGTGACCCTATTCGAGGAGATCCCCGTGGAATATCTGGTCGTCTTAATGGGACCTCTGAAATGTGGGGTCATCATCCA

CAAATGTCCCATAACCAGTTGCAAGGTATCAACAAAATGGTTGGTCAAAGTGTAGCAACTGCCAGCACCAGTGTCGGAACA

TCTGGCTCGGGCATCGGTCCTGGAGGTCCCGGTCCTAGTACAGTATCAGGCAATATCCCAACACAGTGGGGGCCTGCTCAA

CCGGTAAGCGTTGGTGTAAGTGGTCCCAAAGACATGTCAAAACAGATAAGTGGATGGGAGGAACCATCACCACCGCCTCAG

CGTCGCAGTATTCCTAACTACGATGATGGTACATCGTTGTGGGGTCAGCAAACTCGTGTTCCCGCTGCAAGCGGTCACTGG

AAAGACATGACTGATTCGATAGGTCGTAGTAGTCATCTCATGCGTGGCCAAAGCCAAACGGGAGGTATAGGAATAGCCGGC

GTTGGAAATAGCAATGTTCCAGTGGGAGCCAATCCAAGTAATCCTATAAGCAGTGTAGTTGGACCTCAAGCCCGGATTCCA

TCTGTGGGCGGCGTACAACACAAACCAGACGGCGGCGCTATGTGGGTGCATTCCGGCAATGTAGGTGGCAGAAATAATGTT

GCTGCTGTTACTACTTGGGGAGATGACACTCATAGCGTTAATGTCGGCGCTCCCAGCAGTGGCAGTGTATCCAGCAACAAT

TGGGTTGATGACAAGTCCAACTCAACCTTGGCACAAAACTCTTGGAGCGACCCGGCCCCTGTTGGAGTTAGTTGGGGCAAT

AAGCAAAGCAAACCGCCAAGCAATAGTGCTTCATCAGGTTGGAGCACTGCTGCGGGCGTGGTGGATGGGGTTGATCTAGGA

TCTGAGTGGAACACGCACGGGGGGATTATTGGAAAATCTCAGCAACAACAAAAACTAGCGGGACTTAACGTGGGAATGTCT

TATGCCGACCACAATAGTTCAACAAGCAGCGGTGGATTTGCTGGTCGTTACCCGGTCAACAGTGGACAACCTTCAATGTCC

TTTCCTCATAATAACCTTATGAATAACATGGGAGGTACCGCTGTTACTGGAGGTAACAACAATACAAACATGACAGCTTTA

CAGGTGCAAAAGTATTTAAATCAAGGGCAACATGGTGTCGCTGTTGGACCGCAAGCCGTTGGTAATTCTTCAGCAGTATCT

GTCGGATTTGGTCAGAACACGTCTAACGCAGCAGTGGCAGGAGCAGCCTCTGTAAATATAGCAGCAAATACAAACAACCAA

CCGTCTGGTCAGCAAATTCGCATGCTAGGCCAGCAAATTCAGTTGGCCATTCATAGTGGTTTCATATCTAGTCAGATATTG

ACTCAACCGCTAACTCAAACAACCCTTAACCTTTTAAACCAACTTCTTAGCAATATTAAGCATCTCCAGGCTGCGCAGCAA

TCCCTTACCCGCGGGGGAAATGTCAATCCAATGGCAGTGAATGTGGCTATATCTAAATACAAGCAGCAAATCCAGAATTTA

CAGAACCAGATAAATGCACAACAGGCTGTGTATGTAAAACAGCAAAATATGCAACCAACTTCACAACAACAACAGCCCCAA

CAACAGCAACTTCCTTCTGTTCATCTAAGTAACTCAGGCAACGACTATTTAAGAGGTCACGATGCAATAAATAATTTGCAA

AGCAACTTTTCTGAGCTCAATATTAATAAGCCAAGTGGATATCAAGGAGCGTCCAATCAACAATCCCGATTAAATCAGTGG

AAGCTTCCAGTATTAGATAAGGAGATCAACTCTGACAGTACGGAATTTTCTCGTGCCCCAGGTGCAACGAAACAAAATTTG

ACGGCCAACACAAGCAACATAAACTCTTTGGGTCTTCAAAACGATAGTACATGGTCAACTGGACGCAGTATTGGTGACGGT

TGGCCTGATCCCTCATCTGATAACGAGAATAAAGACTGGTCTGTTGCTCAGCCAACTTCAGCAGCAACTGCTTACACTGAT

CTGGTCCAAGAGTTTGAGCCAGGCAAGCCATGGAAGGGTTCACAGATCAAAAGCATAGAAGATGATCCCAGCATTACACCA

GGAAGCGTTGCTAGATCTCCATTGTCTATTAATTCGACGCCAAAAGATGCTGACATATTTGCCAATACCGGTAAAAATTCA

CCGACTGATTTACCGCCACTAAGTTTATCGTCGTCTACATGGAGTTTTAATCCAAACCAAAATTATCCGAGTCACAGTTGG

TCTGACAATAGTCAACAATGTACCGCCACTTCGGAGCTTTGGACAAGCCCGCTAAATAAATCATCGTCTCGAGGTCCCCCG

CCAGGATTGACTGCCAATTCAAATAAATCTGCAAATAGTAATGCGTCAACGCCAACAACTATTACCGGAGGTGCGAATGGA

TGGTTACAGCCTCGAAGTGGCGGTGTTCAAACCACAAACACTAATTGGACAGGTGGTAACACCACTTGGGGCTCCAGTTGG

TTGCTTTTGAAAAATCTAACAGCACAGATTGATGGTCCTACTTTGCGTACACTGTGTATGCAGCATGGGCCCCTTGTCAGC

TTTCACCCGTATTTGAACCAAGGAATTGCCTTATGTAAATATACTACTCGTGAGGAGGCGAACAAGGCGCAAATGGCGTTA

AACAACTGTGTCCTCGCCAACACCACAATATTTGCTGAATCTCCCAGCGAGAACGAGGTGCAAAGCATTATGCAGCACTTA
CCACAAACTCCTTCCTCTACAAGCTCTAGTGGAACTAGTGGTGGCAACGTCGGAGGCGTCGGCACTTCAGCCAATAATGCA
AACAGTGGTTCTGCAGCTTGTCTGTCCGGAAACAATAGCGGCAACGGAAACGGCAGCGCGAGCGGCGCCGGCAGCGGCAAC
AATGGCAACAGTAGCTGCAACAACAGTGCCGCCGGGGGGGGCAGCAGCAGCAACAACACGATTACCACTGTAGCAAATTCG
AATCTTGTTGGTTCTAGTGGCTCTGTCTCAAATTCCTCTGGCGTTACTGCTAACTCTAGTACTGTTTCTGTAGTTAGTTGT
ACAGCGAGTGGGAATTCCATAAATGGGGCAGGTACTGCAAACAGTTCTGGTTCAAAGAGTAGTGCAAACAATTTAGCTAGC
GGCCAGTCTAGCGCTTCTAACTTAACTAATAGCACCAATTCAACATGGCGACAAACTAGCCAAAACCAAGCTCTTCAAAGT
CAAAGCAGGCCATCAGGCAGAGAAGCTGACTTTGATTATATATCTCTCGTTTATTCCATTGTTGATGATTAA

**Hy_pMT Flag_GW182_ΔQ-rich MCS SV40**

**(GW182 is depleted of glutamine-rich region.)**

ATGCGTGAAGCCCTTTTTTCCCAAGATGGCTGGGGCTGTCAGCATGTTAACCAGGATACTAATTGGGAAGTTCCCAGTTCG
CCAGAACCAGCCAATAAGGATGCACCCGGTCCACCAATGTGGAAGCCAAGCATTAACAATGGTACTGATCTTTGGGAGTCC
AATTTGAGAAACGGAGGTCAGCCGGCCGCACAGCAAGTTCCAAAGCCGTCGTGGGGTCATACACCATCCTCTAACTTAGGT
GGAACATGGGGTGAGGACGACGATGGCGCCGATAGTAGTAGTGTGTGGACTGGAGGAGCTGTTAGCAACGCGGGATCCGGA
GCTGCAGTGGGAGTAAACCAAGCCGGAGTTAATGTCGGTCCAGGCGGTGTTGTTTCGTCTGGCGGACCTCAGTGGGGACAA
GGTGTCGTTGGCGTCGGACTTGGATCAACTGGAGGTAACGGGTCAAGCAATATAACTGGATCGTCTGGAGTCGCAACAGGT
AGTAGCGGAAACTCCAGCAACGCTGGTAACGGTTGGGGAGACCCTCGTGAAATACGCCCTTTGGGAGTTGGTGGCTCCATG
GATATTCGAAATGTTGAACATCGCGGCGGTAACGGTTCTGGAGCAACTTCGAGCGATCCACGAGACATTCGCATGATCGAT
CCGCGTGACCCTATTCGAGGAGATCCCCGTGGAATATCTGGTCGTCTTAATGGGACCTCTGAAATGTGGGGTCATCATCCA
CAAATGTCCCATAACCAGTTGCAAGGTATCAACAAAATGGTTGGTCAAAGTGTAGCAACTGCCAGCACCAGTGTCGGAACA
TCTGGCTCGGGCATCGGTCCTGGAGGTCCCGGTCCTAGTACAGTATCAGGCAATATCCCAACACAGTGGGGGCCTGCTCAA
CCGGTAAGCGTTGGTGTAAGTGGTCCCAAAGACATGTCAAAACAGATAAGTGGATGGGAGGAACCATCACCACCGCCTCAG
CGTCGCAGTATTCCTAACTACGATGATGGTACATCGTTGTGGGGTCAGCAAACTCGTGTTCCCGCTGCAAGCGGTCACTGG
AAAGACATGACTGATTCGATAGGTCGTAGTAGTCATCTCATGCGTGGCCAAAGCCAAACGGGAGGTATAGGAATAGCCGGC
GTTGGAAATAGCAATGTTCCAGTGGGAGCCAATCCAAGTAATCCTATAAGCAGTGTAGTTGGACCTCAAGCCCGGATTCCA
TCTGTGGGCGGCGTACAACACAAACCAGACGGCGGCGCTATGTGGGTGCATTCCGGCAATGTAGGTGGCAGAAATAATGTT
GCTGCTGTTACTACTTGGGGAGATGACACTCATAGCGTTAATGTCGGCGCTCCCAGCAGTGGCAGTGTATCCAGCAACAAT
TGGGTTGATGACAAGTCCAACTCAACCTTGGCACAAAACTCTTGGAGCGACCCGGCCCCTGTTGGAGTTAGTTGGGGCAAT
AAGCAAAGCAAACCGCCAAGCAATAGTGCTTCATCAGGTTGGAGCACTGCTGCGGGCGTGGTGGATGGGGTTGATCTAGGA
TCTGAGTGGAACACGCACGGGGGGATTATTGGAAAATCTCAGCAACAACAAAAACTAGCGGGACTTAACGTGGGAATGGTG
AACGTAATTAACGCGGAGATCATTAAGCAAAGCAAGCAATACAGGATCCTTGTCGAGAACGGCTTTAAAAAGGAAGATGTA
GAGCGGGCATTAGTGATTGCTAATATGAACATCGAAGAGGCAGCCGATATGCTCCGTGCCAACTCATCCCTATCAATGGAT

GGTTGGCGTCGACATGATGAGTCCCTTGGATCTTATGCCGACCACAATAGTTCAACAAGCAGCGGTGGATTTGCTGGTCGT

TACCCGGTCAACAGTGGACAACCTTCAATGTCCTTTCCTCATTATCAAGGAGCGTCCAATCAACAATCCCGATTAAATCAG

TGGAAGCTTCCAGTATTAGATAAGGAGATCAACTCTGACAGTACGGAATTTTCTCGTGCCCCAGGTGCAACGAAACAAAAT

TTGACGGCCAACACAAGCAACATAAACTCTTTGGGTCTTCAAAACGATAGTACATGGTCAACTGGACGCAGTATTGGTGAC

GGTTGGCCTGATCCCTCATCTGATAACGAGAATAAAGACTGGTCTGTTGCTCAGCCAACTTCAGCAGCAACTGCTTACACT

GATCTGGTCCAAGAGTTTGAGCCAGGCAAGCCATGGAAGGGTTCACAGATCAAAAGCATAGAAGATGATCCCAGCATTACA

CCAGGAAGCGTTGCTAGATCTCCATTGTCTATTAATTCGACGCCAAAAGATGCTGACATATTTGCCAATACCGGTAAAAAT

TCACCGACTGATTTACCGCCACTAAGTTTATCGTCGTCTACATGGAGTTTTAATCCAAACCAAAATTATCCGAGTCACAGT

TGGTCTGACAATAGTCAACAATGTACCGCCACTTCGGAGCTTTGGACAAGCCCGCTAAATAAATCATCGTCTCGAGGTCCC

CCGCCAGGATTGACTGCCAATTCAAATAAATCTGCAAATAGTAATGCGTCAACGCCAACAACTATTACCGGAGGTGCGAAT

GGATGGTTACAGCCTCGAAGTGGCGGTGTTCAAACCACAAACACTAATTGGACAGGTGGTAACACCACTTGGGGCTCCAGT

TGGTTGCTTTTGAAAAATCTAACAGCACAGATTGATGGTCCTACTTTGCGTACACTGTGTATGCAGCATGGGCCCCTTGTC

AGCTTTCACCCGTATTTGAACCAAGGAATTGCCTTATGTAAATATACTACTCGTGAGGAGGCGAACAAGGCGCAAATGGCG

TTAAACAACTGTGTCCTCGCCAACACCACAATATTTGCTGAATCTCCCAGCGAGAACGAGGTGCAAAGCATTATGCAGCAC

TTACCACAAACTCCTTCCTCTACAAGCTCTAGTGGAACTAGTGGTGGCAACGTCGGAGGCGTCGGCACTTCAGCCAATAAT

GCAAACAGTGGTTCTGCAGCTTGTCTGTCCGGAAACAATAGCGGCAACGGAAACGGCAGCGCGAGCGGCGCCGGCAGCGGC

AACAATGGCAACAGTAGCTGCAACAACAGTGCCGCCGGGGGGGGCAGCAGCAGCAACAACACGATTACCACTGTAGCAAAT

TCGAATCTTGTTGGTTCTAGTGGCTCTGTCTCAAATTCCTCTGGCGTTACTGCTAACTCTAGTACTGTTTCTGTAGTTAGT

TGTACAGCGAGTGGGAATTCCATAAATGGGGCAGGTACTGCAAACAGTTCTGGTTCAAAGAGTAGTGCAAACAATTTAGCT

AGCGGCCAGTCTAGCGCTTCTAACTTAACTAATAGCACCAATTCAACATGGCGACAAACTAGCCAAAACCAAGCTCTTCAA

AGTCAAAGCAGGCCATCAGGCAGAGAAGCTGACTTTGATTATATATCTCTCGTTTATTCCATTGTTGATGATTAA

**Hy_pMT Flag_GW182_ΔMid MCS SV40**

**(GW182 is depleted of middle region.)**

ATGCGTGAAGCCCTTTTTTCCCAAGATGGCTGGGGCTGTCAGCATGTTAACCAGGATACTAATTGGGAAGTTCCCAGTTCG

CCAGAACCAGCCAATAAGGATGCACCCGGTCCACCAATGTGGAAGCCAAGCATTAACAATGGTACTGATCTTTGGGAGTCC

AATTTGAGAAACGGAGGTCAGCCGGCCGCACAGCAAGTTCCAAAGCCGTCGTGGGGTCATACACCATCCTCTAACTTAGGT

GGAACATGGGGTGAGGACGACGATGGCGCCGATAGTAGTAGTGTGTGGACTGGAGGAGCTGTTAGCAACGCGGGATCCGGA

GCTGCAGTGGGAGTAAACCAAGCCGGAGTTAATGTCGGTCCAGGCGGTGTTGTTTCGTCTGGCGGACCTCAGTGGGGACAA

GGTGTCGTTGGCGTCGGACTTGGATCAACTGGAGGTAACGGGTCAAGCAATATAACTGGATCGTCTGGAGTCGCAACAGGT

AGTAGCGGAAACTCCAGCAACGCTGGTAACGGTTGGGGAGACCCTCGTGAAATACGCCCTTTGGGAGTTGGTGGCTCCATG

GATATTCGAAATGTTGAACATCGCGGCGGTAACGGTTCTGGAGCAACTTCGAGCGATCCACGAGACATTCGCATGATCGAT

CCGCGTGACCCTATTCGAGGAGATCCCCGTGGAATATCTGGTCGTCTTAATGGGACCTCTGAAATGTGGGGTCATCATCCA

CAAATGTCCCATAACCAGTTGCAAGGTATCAACAAAATGGTTGGTCAAAGTGTAGCAACTGCCAGCACCAGTGTCGGAACA
TCTGGCTCGGGCATCGGTCCTGGAGGTCCCGGTCCTAGTACAGTATCAGGCAATATCCCAACACAGTGGGGGCCTGCTCAA
CCGGTAAGCGTTGGTGTAAGTGGTCCCAAAGACATGTCAAAACAGATAAGTGGATGGGAGGAACCATCACCACCGCCTCAG
CGTCGCAGTATTCCTAACTACGATGATGGTACATCGTTGTGGGGTCAGCAAACTCGTGTTCCCGCTGCAAGCGGTCACTGG
AAAGACATGACTGATTCGATAGGTCGTAGTAGTCATCTCATGCGTGGCCAAAGCCAAACGGGAGGTATAGGAATAGCCGGC
GTTGGAAATAGCAATGTTCCAGTGGGAGCCAATCCAAGTAATCCTATAAGCAGTGTAGTTGGACCTCAAGCCCGGATTCCA
TCTGTGGGCGGCGTACAACACAAACCAGACGGCGGCGCTATGTGGGTGCATTCCGGCAATGTAGGTGGCAGAAATAATGTT
GCTGCTGTTACTACTTGGGGAGATGACACTCATAGCGTTAATGTCGGCGCTCCCAGCAGTGGCAGTGTATCCAGCAACAAT
TGGGTTGATGACAAGTCCAACTCAACCTTGGCACAAAACTCTTGGAGCGACCCGGCCCCTGTTGGAGTTAGTTGGGGCAAT
AAGCAAAGCAAACCGCCAAGCAATAGTGCTTCATCAGGTTGGAGCACTGCTGCGGGCGTGGTGGATGGGGTTGATCTAGGA
TCTGAGTGGAACACGCACGGGGGGATTATTGGAAAATCTCAGCAACAACAAAAACTAGCGGGACTTAACGTGGGAATGGTG
AACGTAATTAACGCGGAGATCATTAAGCAAAGCAAGCAATACAGGATCCTTGTCGAGAACGGCTTTAAAAAGGAAGATGTA
GAGCGGGCATTAGTGATTGCTAATATGAACATCGAAGAGGCAGCCGATATGCTCCGTGCCAACTCATCCCTATCAATGGAT
GGTTGGCGTCGACATGATGAGTCCCTTGGATCTTATGCCGACCACAATAGTTCAACAAGCAGCGGTGGATTTGCTGGTCGT
TACCCGGTCAACAGTGGACAACCTTCAATGTCCTTTCCTCATAATAACCTTATGAATAACATGGGAGGTACCGCTGTTACT
GGAGGTAACAACAATACAAACATGACAGCTTTACAGGTGCAAAAGTATTTAAATCAAGGGCAACATGGTGTCGCTGTTGGA
CCGCAAGCCGTTGGTAATTCTTCAGCAGTATCTGTCGGATTTGGTCAGAACACGTCTAACGCAGCAGTGGCAGGAGCAGCC
TCTGTAAATATAGCAGCAAATACAAACAACCAACCGTCTGGTCAGCAAATTCGCATGCTAGGCCAGCAAATTCAGTTGGCC
ATTCATAGTGGTTTCATATCTAGTCAGATATTGACTCAACCGCTAACTCAAACAACCCTTAACCTTTTAAACCAACTTCTT
AGCAATATTAAGCATCTCCAGGCTGCGCAGCAATCCCTTACCCGCGGGGGAAATGTCAATCCAATGGCAGTGAATGTGGCT
ATATCTAAATACAAGCAGCAAATCCAGAATTTACAGAACCAGATAAATGCACAACAGGCTGTGTATGTAAAACAGCAAAAT
ATGCAACCAACTTCACAACAACAACAGCCCCAACAACAGCAACTTCCTTCTGTTCATCTAAGTAACTCAGGCAACGACTAT
TTAAGAGGTCACGATGCAATAAATAATTTGCAAAGCAACTTTTCTGAGCTCAATATTAATAAGCCAAGTGGAAGTTGGTTG
CTTTTGAAAAATCTAACAGCACAGATTGATGGTCCTACTTTGCGTACACTGTGTATGCAGCATGGGCCCCTTGTCAGCTTT
CACCCGTATTTGAACCAAGGAATTGCCTTATGTAAATATACTACTCGTGAGGAGGCGAACAAGGCGCAAATGGCGTTAAAC
AACTGTGTCCTCGCCAACACCACAATATTTGCTGAATCTCCCAGCGAGAACGAGGTGCAAAGCATTATGCAGCACTTACCA
CAAACTCCTTCCTCTACAAGCTCTAGTGGAACTAGTGGTGGCAACGTCGGAGGCGTCGGCACTTCAGCCAATAATGCAAAC
AGTGGTTCTGCAGCTTGTCTGTCCGGAAACAATAGCGGCAACGGAAACGGCAGCGCGAGCGGCGCCGGCAGCGGCAACAAT
GGCAACAGTAGCTGCAACAACAGTGCCGCCGGGGGGGGCAGCAGCAGCAACAACACGATTACCACTGTAGCAAATTCGAAT
CTTGTTGGTTCTAGTGGCTCTGTCTCAAATTCCTCTGGCGTTACTGCTAACTCTAGTACTGTTTCTGTAGTTAGTTGTACA
GCGAGTGGGAATTCCATAAATGGGGCAGGTACTGCAAACAGTTCTGGTTCAAAGAGTAGTGCAAACAATTTAGCTAGCGGC
CAGTCTAGCGCTTCTAACTTAACTAATAGCACCAATTCAACATGGCGACAAACTAGCCAAAACCAAGCTCTTCAAAGTCAA
AGCAGGCCATCAGGCAGAGAAGCTGACTTTGATTATATATCTCTCGTTTATTCCATTGTTGATGATTAA

**Hy_pMT Flag_GW182_ΔRRM MCS SV40**

**(GW182 is depleted of RNA recognition motif.)**

ATGCGTGAAGCCCTTTTTTCCCAAGATGGCTGGGGCTGTCAGCATGTTAACCAGGATACTAATTGGGAAGTTCCCAGTTCG

CCAGAACCAGCCAATAAGGATGCACCCGGTCCACCAATGTGGAAGCCAAGCATTAACAATGGTACTGATCTTTGGGAGTCC

AATTTGAGAAACGGAGGTCAGCCGGCCGCACAGCAAGTTCCAAAGCCGTCGTGGGGTCATACACCATCCTCTAACTTAGGT

GGAACATGGGGTGAGGACGACGATGGCGCCGATAGTAGTAGTGTGTGGACTGGAGGAGCTGTTAGCAACGCGGGATCCGGA

GCTGCAGTGGGAGTAAACCAAGCCGGAGTTAATGTCGGTCCAGGCGGTGTTGTTTCGTCTGGCGGACCTCAGTGGGGACAA

GGTGTCGTTGGCGTCGGACTTGGATCAACTGGAGGTAACGGGTCAAGCAATATAACTGGATCGTCTGGAGTCGCAACAGGT

AGTAGCGGAAACTCCAGCAACGCTGGTAACGGTTGGGGAGACCCTCGTGAAATACGCCCTTTGGGAGTTGGTGGCTCCATG

GATATTCGAAATGTTGAACATCGCGGCGGTAACGGTTCTGGAGCAACTTCGAGCGATCCACGAGACATTCGCATGATCGAT

CCGCGTGACCCTATTCGAGGAGATCCCCGTGGAATATCTGGTCGTCTTAATGGGACCTCTGAAATGTGGGGTCATCATCCA

CAAATGTCCCATAACCAGTTGCAAGGTATCAACAAAATGGTTGGTCAAAGTGTAGCAACTGCCAGCACCAGTGTCGGAACA

TCTGGCTCGGGCATCGGTCCTGGAGGTCCCGGTCCTAGTACAGTATCAGGCAATATCCCAACACAGTGGGGGCCTGCTCAA

CCGGTAAGCGTTGGTGTAAGTGGTCCCAAAGACATGTCAAAACAGATAAGTGGATGGGAGGAACCATCACCACCGCCTCAG

CGTCGCAGTATTCCTAACTACGATGATGGTACATCGTTGTGGGGTCAGCAAACTCGTGTTCCCGCTGCAAGCGGTCACTGG

AAAGACATGACTGATTCGATAGGTCGTAGTAGTCATCTCATGCGTGGCCAAAGCCAAACGGGAGGTATAGGAATAGCCGGC

GTTGGAAATAGCAATGTTCCAGTGGGAGCCAATCCAAGTAATCCTATAAGCAGTGTAGTTGGACCTCAAGCCCGGATTCCA

TCTGTGGGCGGCGTACAACACAAACCAGACGGCGGCGCTATGTGGGTGCATTCCGGCAATGTAGGTGGCAGAAATAATGTT

GCTGCTGTTACTACTTGGGGAGATGACACTCATAGCGTTAATGTCGGCGCTCCCAGCAGTGGCAGTGTATCCAGCAACAAT

TGGGTTGATGACAAGTCCAACTCAACCTTGGCACAAAACTCTTGGAGCGACCCGGCCCCTGTTGGAGTTAGTTGGGGCAAT

AAGCAAAGCAAACCGCCAAGCAATAGTGCTTCATCAGGTTGGAGCACTGCTGCGGGCGTGGTGGATGGGGTTGATCTAGGA

TCTGAGTGGAACACGCACGGGGGGATTATTGGAAAATCTCAGCAACAACAAAAACTAGCGGGACTTAACGTGGGAATGGTG

AACGTAATTAACGCGGAGATCATTAAGCAAAGCAAGCAATACAGGATCCTTGTCGAGAACGGCTTTAAAAAGGAAGATGTA

GAGCGGGCATTAGTGATTGCTAATATGAACATCGAAGAGGCAGCCGATATGCTCCGTGCCAACTCATCCCTATCAATGGAT

GGTTGGCGTCGACATGATGAGTCCCTTGGATCTTATGCCGACCACAATAGTTCAACAAGCAGCGGTGGATTTGCTGGTCGT

TACCCGGTCAACAGTGGACAACCTTCAATGTCCTTTCCTCATAATAACCTTATGAATAACATGGGAGGTACCGCTGTTACT

GGAGGTAACAACAATACAAACATGACAGCTTTACAGGTGCAAAAGTATTTAAATCAAGGGCAACATGGTGTCGCTGTTGGA

CCGCAAGCCGTTGGTAATTCTTCAGCAGTATCTGTCGGATTTGGTCAGAACACGTCTAACGCAGCAGTGGCAGGAGCAGCC

TCTGTAAATATAGCAGCAAATACAAACAACCAACCGTCTGGTCAGCAAATTCGCATGCTAGGCCAGCAAATTCAGTTGGCC

ATTCATAGTGGTTTCATATCTAGTCAGATATTGACTCAACCGCTAACTCAAACAACCCTTAACCTTTTAAACCAACTTCTT

AGCAATATTAAGCATCTCCAGGCTGCGCAGCAATCCCTTACCCGCGGGGGAAATGTCAATCCAATGGCAGTGAATGTGGCT

ATATCTAAATACAAGCAGCAAATCCAGAATTTACAGAACCAGATAAATGCACAACAGGCTGTGTATGTAAAACAGCAAAAT

ATGCAACCAACTTCACAACAACAACAGCCCCAACAACAGCAACTTCCTTCTGTTCATCTAAGTAACTCAGGCAACGACTAT

TTAAGAGGTCACGATGCAATAAATAATTTGCAAAGCAACTTTTCTGAGCTCAATATTAATAAGCCAAGTGGATATCAAGGA

GCGTCCAATCAACAATCCCGATTAAATCAGTGGAAGCTTCCAGTATTAGATAAGGAGATCAACTCTGACAGTACGGAATTT

TCTCGTGCCCCAGGTGCAACGAAACAAAATTTGACGGCCAACACAAGCAACATAAACTCTTTGGGTCTTCAAAACGATAGT

ACATGGTCAACTGGACGCAGTATTGGTGACGGTTGGCCTGATCCCTCATCTGATAACGAGAATAAAGACTGGTCTGTTGCT

CAGCCAACTTCAGCAGCAACTGCTTACACTGATCTGGTCCAAGAGTTTGAGCCAGGCAAGCCATGGAAGGGTTCACAGATC

AAAAGCATAGAAGATGATCCCAGCATTACACCAGGAAGCGTTGCTAGATCTCCATTGTCTATTAATTCGACGCCAAAAGAT

GCTGACATATTTGCCAATACCGGTAAAAATTCACCGACTGATTTACCGCCACTAAGTTTATCGTCGTCTACATGGAGTTTT

AATCCAAACCAAAATTATCCGAGTCACAGTTGGTCTGACAATAGTCAACAATGTACCGCCACTTCGGAGCTTTGGACAAGC

CCGCTAAATAAATCATCGTCTCGAGGTCCCCCGCCAGGATTGACTGCCAATTCAAATAAATCTGCAAATAGTAATGCGTCA

ACGCCAACAACTATTACCGGAGGTGCGAATGGATGGTTACAGCCTCGAAGTGGCGGTGTTCAAACCACAAACACTAATTGG

ACAGGTGGTAACACCACTTGGGGCTCCTTACCACAAACTCCTTCCTCTACAAGCTCTAGTGGAACTAGTGGTGGCAACGTC

GGAGGCGTCGGCACTTCAGCCAATAATGCAAACAGTGGTTCTGCAGCTTGTCTGTCCGGAAACAATAGCGGCAACGGAAAC

GGCAGCGCGAGCGGCGCCGGCAGCGGCAACAATGGCAACAGTAGCTGCAACAACAGTGCCGCCGGGGGGGGCAGCAGCAGC

AACAACACGATTACCACTGTAGCAAATTCGAATCTTGTTGGTTCTAGTGGCTCTGTCTCAAATTCCTCTGGCGTTACTGCT

AACTCTAGTACTGTTTCTGTAGTTAGTTGTACAGCGAGTGGGAATTCCATAAATGGGGCAGGTACTGCAAACAGTTCTGGT

TCAAAGAGTAGTGCAAACAATTTAGCTAGCGGCCAGTCTAGCGCTTCTAACTTAACTAATAGCACCAATTCAACATGGCGA

CAAACTAGCCAAAACCAAGCTCTTCAAAGTCAAAGCAGGCCATCAGGCAGAGAAGCTGACTTTGATTATATATCTCTCGTT

TATTCCATTGTTGATGATTAA

**Hy_pMT Flag_GW182_ΔC-term MCS SV40**

**(GW182 is depleted of C-terminal region.)**

ATGCGTGAAGCCCTTTTTTCCCAAGATGGCTGGGGCTGTCAGCATGTTAACCAGGATACTAATTGGGAAGTTCCCAGTTCG

CCAGAACCAGCCAATAAGGATGCACCCGGTCCACCAATGTGGAAGCCAAGCATTAACAATGGTACTGATCTTTGGGAGTCC

AATTTGAGAAACGGAGGTCAGCCGGCCGCACAGCAAGTTCCAAAGCCGTCGTGGGGTCATACACCATCCTCTAACTTAGGT

GGAACATGGGGTGAGGACGACGATGGCGCCGATAGTAGTAGTGTGTGGACTGGAGGAGCTGTTAGCAACGCGGGATCCGGA

GCTGCAGTGGGAGTAAACCAAGCCGGAGTTAATGTCGGTCCAGGCGGTGTTGTTTCGTCTGGCGGACCTCAGTGGGGACAA

GGTGTCGTTGGCGTCGGACTTGGATCAACTGGAGGTAACGGGTCAAGCAATATAACTGGATCGTCTGGAGTCGCAACAGGT

AGTAGCGGAAACTCCAGCAACGCTGGTAACGGTTGGGGAGACCCTCGTGAAATACGCCCTTTGGGAGTTGGTGGCTCCATG

GATATTCGAAATGTTGAACATCGCGGCGGTAACGGTTCTGGAGCAACTTCGAGCGATCCACGAGACATTCGCATGATCGAT

CCGCGTGACCCTATTCGAGGAGATCCCCGTGGAATATCTGGTCGTCTTAATGGGACCTCTGAAATGTGGGGTCATCATCCA

CAAATGTCCCATAACCAGTTGCAAGGTATCAACAAAATGGTTGGTCAAAGTGTAGCAACTGCCAGCACCAGTGTCGGAACA

TCTGGCTCGGGCATCGGTCCTGGAGGTCCCGGTCCTAGTACAGTATCAGGCAATATCCCAACACAGTGGGGGCCTGCTCAA

CCGGTAAGCGTTGGTGTAAGTGGTCCCAAAGACATGTCAAAACAGATAAGTGGATGGGAGGAACCATCACCACCGCCTCAG

CGTCGCAGTATTCCTAACTACGATGATGGTACATCGTTGTGGGGTCAGCAAACTCGTGTTCCCGCTGCAAGCGGTCACTGG

AAAGACATGACTGATTCGATAGGTCGTAGTAGTCATCTCATGCGTGGCCAAAGCCAAACGGGAGGTATAGGAATAGCCGGC
GTTGGAAATAGCAATGTTCCAGTGGGAGCCAATCCAAGTAATCCTATAAGCAGTGTAGTTGGACCTCAAGCCCGGATTCCA
TCTGTGGGCGGCGTACAACACAAACCAGACGGCGGCGCTATGTGGGTGCATTCCGGCAATGTAGGTGGCAGAAATAATGTT
GCTGCTGTTACTACTTGGGGAGATGACACTCATAGCGTTAATGTCGGCGCTCCCAGCAGTGGCAGTGTATCCAGCAACAAT
TGGGTTGATGACAAGTCCAACTCAACCTTGGCACAAAACTCTTGGAGCGACCCGGCCCCTGTTGGAGTTAGTTGGGGCAAT
AAGCAAAGCAAACCGCCAAGCAATAGTGCTTCATCAGGTTGGAGCACTGCTGCGGGCGTGGTGGATGGGGTTGATCTAGGA
TCTGAGTGGAACACGCACGGGGGGATTATTGGAAAATCTCAGCAACAACAAAAACTAGCGGGACTTAACGTGGGAATGGTG
AACGTAATTAACGCGGAGATCATTAAGCAAAGCAAGCAATACAGGATCCTTGTCGAGAACGGCTTTAAAAAGGAAGATGTA
GAGCGGGCATTAGTGATTGCTAATATGAACATCGAAGAGGCAGCCGATATGCTCCGTGCCAACTCATCCCTATCAATGGAT
GGTTGGCGTCGACATGATGAGTCCCTTGGATCTTATGCCGACCACAATAGTTCAACAAGCAGCGGTGGATTTGCTGGTCGT
TACCCGGTCAACAGTGGACAACCTTCAATGTCCTTTCCTCATAATAACCTTATGAATAACATGGGAGGTACCGCTGTTACT
GGAGGTAACAACAATACAAACATGACAGCTTTACAGGTGCAAAAGTATTTAAATCAAGGGCAACATGGTGTCGCTGTTGGA
CCGCAAGCCGTTGGTAATTCTTCAGCAGTATCTGTCGGATTTGGTCAGAACACGTCTAACGCAGCAGTGGCAGGAGCAGCC
TCTGTAAATATAGCAGCAAATACAAACAACCAACCGTCTGGTCAGCAAATTCGCATGCTAGGCCAGCAAATTCAGTTGGCC
ATTCATAGTGGTTTCATATCTAGTCAGATATTGACTCAACCGCTAACTCAAACAACCCTTAACCTTTTAAACCAACTTCTT
AGCAATATTAAGCATCTCCAGGCTGCGCAGCAATCCCTTACCCGCGGGGGAAATGTCAATCCAATGGCAGTGAATGTGGCT
ATATCTAAATACAAGCAGCAAATCCAGAATTTACAGAACCAGATAAATGCACAACAGGCTGTGTATGTAAAACAGCAAAAT
ATGCAACCAACTTCACAACAACAACAGCCCCAACAACAGCAACTTCCTTCTGTTCATCTAAGTAACTCAGGCAACGACTAT
TTAAGAGGTCACGATGCAATAAATAATTTGCAAAGCAACTTTTCTGAGCTCAATATTAATAAGCCAAGTGGATATCAAGGA
GCGTCCAATCAACAATCCCGATTAAATCAGTGGAAGCTTCCAGTATTAGATAAGGAGATCAACTCTGACAGTACGGAATTT
TCTCGTGCCCCAGGTGCAACGAAACAAAATTTGACGGCCAACACAAGCAACATAAACTCTTTGGGTCTTCAAAACGATAGT
ACATGGTCAACTGGACGCAGTATTGGTGACGGTTGGCCTGATCCCTCATCTGATAACGAGAATAAAGACTGGTCTGTTGCT
CAGCCAACTTCAGCAGCAACTGCTTACACTGATCTGGTCCAAGAGTTTGAGCCAGGCAAGCCATGGAAGGGTTCACAGATC
AAAAGCATAGAAGATGATCCCAGCATTACACCAGGAAGCGTTGCTAGATCTCCATTGTCTATTAATTCGACGCCAAAAGAT
GCTGACATATTTGCCAATACCGGTAAAAATTCACCGACTGATTTACCGCCACTAAGTTTATCGTCGTCTACATGGAGTTTT
AATCCAAACCAAAATTATCCGAGTCACAGTTGGTCTGACAATAGTCAACAATGTACCGCCACTTCGGAGCTTTGGACAAGC
CCGCTAAATAAATCATCGTCTCGAGGTCCCCCGCCAGGATTGACTGCCAATTCAAATAAATCTGCAAATAGTAATGCGTCA
ACGCCAACAACTATTACCGGAGGTGCGAATGGATGGTTACAGCCTCGAAGTGGCGGTGTTCAAACCACAAACACTAATTGG
ACAGGTGGTAACACCACTTGGGGCTCCAGTTGGTTGCTTTTGAAAAATCTAACAGCACAGATTGATGGTCCTACTTTGCGT
ACACTGTGTATGCAGCATGGGCCCCTTGTCAGCTTTCACCCGTATTTGAACCAAGGAATTGCCTTATGTAAATATACTACT
CGTGAGGAGGCGAACAAGGCGCAAATGGCGTTAAACAACTGTGTCCTCGCCAACACCACAATATTTGCTGAATCTCCCAGC
GAGAACGAGGTGCAAAGCATTATGCAGCACTAA

**Hy_pMT Flag_GW182_Mid MCS SV40**

**(Mid domain alone)**

TATCAAGGAGCGTCCAATCAACAATCCCGATTAAATCAGTGGAAGCTTCCAGTATTAGATAAGGAGATCAACTCTGACAGT

ACGGAATTTTCTCGTGCCCCAGGTGCAACGAAACAAAATTTGACGGCCAACACAAGCAACATAAACTCTTTGGGTCTTCAA

AACGATAGTACATGGTCAACTGGACGCAGTATTGGTGACGGTTGGCCTGATCCCTCATCTGATAACGAGAATAAAGACTGG

TCTGTTGCTCAGCCAACTTCAGCAGCAACTGCTTACACTGATCTGGTCCAAGAGTTTGAGCCAGGCAAGCCATGGAAGGGT

TCACAGATCAAAAGCATAGAAGATGATCCCAGCATTACACCAGGAAGCGTTGCTAGATCTCCATTGTCTATTAATTCGACG

CCAAAAGATGCTGACATATTTGCCAATACCGGTAAAAATTCACCGACTGATTTACCGCCACTAAGTTTATCGTCGTCTACA

TGGAGTTTTAATCCAAACCAAAATTATCCGAGTCACAGTTGGTCTGACAATAGTCAACAATGTACCGCCACTTCGGAGCTT

TGGACAAGCCCGCTAAATAAATCATCGTCTCGAGGTCCCCCGCCAGGATTGACTGCCAATTCAAATAAATCTGCAAATAGT

AATGCGTCAACGCCAACAACTATTACCGGAGGTGCGAATGGATGGTTACAGCCTCGAAGTGGCGGTGTTCAAACCACAAAC

ACTAATTGGACAGGTGGTAACACCACTTGGGGCTCCTAA

The following plasmid was generated by inserting the indicated sequences into the KpnI and NotI sites of **Hy_pMT V5 MCS SV40**.

### Hy_pMT V5_DCP2 MCS SV40

ATGGAGCTAAACAATCTAATACGTATGTGTTTCCAAATCGAACTGGCCCATTGGTTCTATTTGGATTTCTTTTGTGCCCCC

GAATCTGGCGAGGATGGCGAGACCCCGAAGTGCGTGCAACGAAAGCTGCCATCGGTGGGCATCAAGCAGTTTGCCATGCAG

TTGTTCCAACACATTCCCTTCCTGAACAAACATTTTGGCACCGTGGATCAAATCCTGGACGAGTGGAAGAACTACAAGCTA

TCGGTGCCCACTTATGGCGCTATTCTGGTGTCCGAGGATCACAATCACTGCTTGCTAGTGCAGTCCTACTTCGCGCGCAAC

TCCTGGGGATTTCCCAAGGGCAAGATCAATGAGAACGAAGACCCAGCCCATTGTGCAACGAGAGAGGTTTATGAGGAGACC

GGGTTCGATATCACGGACCTAATCGATGCCAACGACTACATCGAGGCGTTCATCAACTACCAGTACACGCGGCTGTACGTG

GTGCGAAACATACCGATGGACACGCAGTTCGCGCCCCGCACTCGCAACGAGATCAAGTGCTGCGACTGGTTCCGTATCGAT

GCGCTGCCGGTGAACAAGAACGATGCCATATCAAAGGCCAAGTTGGGCAAGACCTCCAACTCCTTCTTCATGATTATGCCG

TTTGTGAAGCGCCTGAAGAAGTGGGTGAACGATCGGAAGGCTGGAATCGAGCCGCGTCGCCGCAAATTCTCTGGTCAGCAA

TCGCCAAAAGCGGCCTCGCCCACCAATATGAACGGAAATTGCAAGAAGGACGTCACAGGAGTGCGAAATGAATCGCAACGA

CAGAGGCACAAGTCCATGGGCGACTTGGATGGCGTCAAATTGAATAATCTCAATGGCAAGGGGACCGTTGGAACGGGAGCG

GGATCGGCAGCTTCCGCCTCCGCCACCGCTGCCATCAACTCGATGAACATATGCAATAGCAATGGGAAACGCAATAAGCAA

AATGCGGCTGCGGGCAGCAATCAGGCAACGCCAACTACAATAGCAACGCCAATGACGTCAAATGGCGCCGTCGTAGGAGGT

ACTGTATCCTCGAAGCGTCAACTGTTCCATAGCCAAAGTCAGAACGATGCGCAGCAATCGGCAAGCAATGAGAAGATTGTC

AGCTCCTTCGATTTAATACGCAAGGAGAAGCAGCAACAACTTAAGGCAGCTAAGGCGCAAAAGCAGCAGCAACAACAGCAG

CAACGATCGCGCACCAAGTCGCAGAGTGATAAGCAGTACAATCCACAGCAGCCAGTCAAAATCCTGGGACAACAGCAGCCC

ACAACGGGCATGGATTTGATAGCCATGCTATCGGCAGCGGCGGCAGCTCAAAAGACACCAAACAACGAGCAGCAAACACAA

GAGAAGCAGCAGCAGCAACAACGACCACGACCTGCTTCAGTGTCGCTTAACCTTGGAGCAGGCAGTGCCACAGGAGCAACA

ACATCAGCTCCGGATGCCCAGTACATGCACAGGCTGCAGCGCATGGCATCGGGCACAAATTTGAGGATTCTGAAGCGGGCA

CAGTCGCAGCAGCCGCAGCAGCACCATCAGCAACAGTTGCAACTAGAACAGCAACAACAGCAGCAGCAGCAGCTGGGAATC

GACTTTACGCCAAATTTGAATTCGTGGACGAATTTCTCCTTTACCAAGAACTTCATAGCAAATGTGTTTTGCTAA
